# Largest genome assembly in Brassicaceae: retrotransposon‐driven genome expansion and karyotype evolution in *Matthiola incana*

**DOI:** 10.1111/pbi.70193

**Published:** 2025-06-26

**Authors:** Daozong Chen, Taihua Yang, Haidong Chen, Xiaohan Zhang, Fan Huang, Shubei Wan, Zhanjun Lu, Chao Liu, Yong Lei, Huifang Jiang, Boshou Liao, Graham J. King, Martin A. Lysak, Chen Tan, Xianhong Ge

**Affiliations:** ^1^ College of Life Sciences, Ganzhou Key Laboratory of Greenhouse Vegetable Gannan Normal University Ganzhou China; ^2^ National Key Laboratory of Crop Genetic Improvement, National Research Center of Rapeseed Engineering and Technology Huazhong Agricultural University Wuhan China; ^3^ Key Laboratory of Biology and Genetic Improvement of Oil Crops, Ministry of Agriculture and Rural Afairs Oil Crops Research Institute of Chinese Academy of Agricultural Sciences (OCRI‐CAAS) Wuhan China; ^4^ Recombics Alstonville 2477 New South Wales Australia; ^5^ Central European Institute of Technology (CEITEC) and Department of Experimental Botany, Faculty of Science Masaryk University Brno Czech Republic

**Keywords:** genome assembly, genome obesity, Lineage III, Hesperodae, Cruciferae, retrotransposons

## Abstract

*Matthiola incana*, commonly known as stock and gillyflower, is a widely grown ornamental plant whose genome is significantly larger than that of other species in the mustard family. However, the evolutionary history behind such a large genome (~2 Gb) is still unknown. Here, we have succeeded in obtaining a high‐quality chromosome‐scale genome assembly of *M. incana* by integrating PacBio HiFi reads, Illumina short reads and Hi‐C data. The resulting genome consists of seven pseudochromosomes with a length of 1965 Mb and 38 245 gene models. Phylogenetic analysis indicates that *M. incana* and other taxa of the supertribe Hesperodae represent an early‐diverging lineage in the evolutionary history of the Brassicaceae. Through a comparative analysis, we revisited the ancestral Hesperodae karyotype (AHK, *n* = 7) and found several differences from the well‐established ancestral crucifer karyotype (ACK, *n* = 8) model, including extensive inter‐ and intra‐chromosomal rearrangements. Our results suggest that the primary reason for genome obesity in *M. incana* is the massive expansion of long terminal repeat retrotransposons (LTR‐RTs), particularly from the Angela, Athila and Retand families. CHG methylation modification is obviously reduced in the regions where the highest density of *Copia*‐type LTR‐RTs and the lowest density of *Gypsy*‐type LTR‐RTs overlap, corresponding to the putative centromeres. Based on insertion times and methylation profiling, recently inserted LTR‐RTs were found to have a significantly different methylation pattern compared to older ones.

## Introduction

The Brassicaceae family (formerly known as Cruciferae) is one of the most diverse and successful families in the plant kingdom, encompassing over 350 genera and over 4100 species (Franzke *et al*., 2011; Nikolov *et al*., 2019; Walden *et al*., 2020). This family includes the model plant *Arabidopsis thaliana* as well as ornamental plants, important agricultural and condiment crops, and medicinal plants (Wu *et al*., 2022). The Brassicaceae family has the highest number of species with sequenced genomes, providing unique opportunities to trace evolutionary history and advance comparative plant genomics (Sun *et al*., 2022b). Based on the analysis of multiple molecular markers, the core Brassicaceae species were initially classified into three major phylogenetic lineages (Lineage I, II and III) (Bailey *et al*., 2006; Beilstein *et al*., 2006, 2008; Franzke *et al*., 2011) and later expanded to five clades (Clade A to E) (Huang *et al*., 2016; Liu *et al*., 2021; Mabry *et al*., 2020; Nikolov *et al*., 2019). Recently, the most comprehensive genus‐level phylogenetic analysis was performed for the family using over 1000 nuclear genes and 60 plastome genes (Hendriks *et al*., 2023). This study led to the description of two subfamilies (Aethionemoideae and Brassicoideae), the latter one comprising five supertribes: Camelinodae (corresponding to Lineage I and Clade A mentioned above), Brassicodae (Lineage II, Clade B), Hesperodae (Lineage III, Clade E), Arabodae (Clade D) and Heliophilodae (Clade C) (German *et al*., 2023; Hendriks *et al*., 2023). Hendriks *et al*. (2023) also uncovered discrepancies in phylogenetic relationships inferred from nuclear and plastome gene data, particularly the position of the Hesperodae. The phylogenetic tree based on nuclear genes indicates that the Hesperodae are sister to the remaining four supertribes (Hendriks *et al*., 2023; Huang *et al*., 2016; Nikolov *et al*., 2019), whereas plastome‐based phylogenetic analyses retrieved the Hesperodae to be sister to the Brassicodae, Heliophilodae and Arabodae, with the Camelinodae being sister to all four supertribes (Guo *et al*., 2017; Hendriks *et al*., 2023).

Initial comparative genomic and cytogenetic analyses of Brassicaceae species proposed the ancestral crucifer karyotype (ACK), consisting of 8 chromosomes divided into 24 genomic blocks (GBs, A‐X). This has greatly facilitated genomic analyses in Brassicaceae and aided in resolving the evolutionary history of karyotype divergence (Schranz *et al*., 2006). Subsequent research integrated the K and L GBs into one GB (K‐L, separated only in *Arabidopsis thaliana*) and the M and N blocks into one GB (M‐N, separated only in *Brassica* species), reducing the total number of GBs to 22 (Lysak *et al*., 2016). In the Hesperodae, the results of comparative cytogenetic mapping indicated that the inferred ancestral karyotype of clade E (CEK) with 7 chromosomes originated from an older genome with 8 chromosome pairs (Mandáková *et al*., 2017a). Recently, genomic studies of Hesperodae species such as *Euclidium syriacum* (*n* = 7; Jiao *et al*., 2017), which lacks a chromosome‐level genome assembly, and *Tetracme quadricornis* (*n* = 7; Liu *et al*., 2024), which have a high‐quality genome assembly at the chromosome level, both attempted to reconstruct the most ancestral Brassicaceae karyotype (CBK) with 9 pseudochromosomes (Liu *et al*., 2024; Walden and Schranz, 2023). However, this analysis was limited by the availability of only one high‐quality Hesperodae genome, emphasizing the need for additional genome assemblies for more species of the supertribe.

The genome sizes of Brassicaceae species vary considerably, ranging from about 157 Mb in *A. thaliana* to about 8120 Mb in the tetraploid *Hesperis matronalis*, reflecting a difference of nearly 52‐fold (Hloušková *et al*., 2019; Kiefer *et al*., 2014). In general, Brassicaceae species have relatively small genomes, with a mean 1C‐value of approximately 610 Mb (Lysak *et al*., 2009). However, analysis of genome size variation in the Brassicaceae shows that some species within the Hesperodae genera have large genomes, for example *Matthiola* (1.56 to 2.87 Gb), *Bunias* (2.02 to 2.74 Gb), *Rhammatophyllum* (1.81 Gb), *Hesperis* (4.28 to 8.12 Gb) and *Clausia* (3.88 Gb) (Kiefer *et al*., 2014). The genome size variation reaches almost 30‐fold in the Hesperodae, with *Diptychocarpus strictus* being the smallest at around 265 Mb (Kiefer *et al*., 2014). *Ty1/Copia* and *Ty3/Gypsy*, the two primary superfamilies of long terminal repeat retrotransposons (LTR‐RTs), are the most widespread transposable elements in plant genomes (Bennetzen and Wang, 2014; Galindo‐González *et al*., 2017). These RTs can account for different proportions in different genomes, ranging from 6.8% in the compact genome of *A. thaliana* to 75% in the complex maize genome (Chen *et al*., 2023; Hou *et al*., 2022). In Brassicaceae, studies have shown that the amplification of specific LTR‐RTs, either in the pericentromeric regions or across entire chromosomes, is a major factor contributing to the increase in genome size, in addition to whole‐genome duplications (Hloušková *et al*., 2019; Lysak *et al*., 2009; Willing *et al*., 2015; Zhang *et al*., 2020).

*Matthiola incana* belongs to the tribe Anchonieae within the Hesperodae. *M. incana*, commonly known as stock or gillyflower, is native to the Mediterranean coast and has gained popularity as an ornamental plant throughout Europe due to its diverse flower colours and delightful fragrance. *M. incana* has many interesting biological characteristics, such as the development of a woody stem, strong cold hardiness, a strong floral scent, a long flowering period and a high content of linolenic acid in its seed oil (Irani and Arab, 2017; Nakatsuka and Koishi, 2018; Nuraini *et al*., 2020). The seeds of *M. incana* contain a high concentration of linolenic acid, accounting for 55–65% of the content, making *M. incana* an important genetic resource for the improvement of oil crops from the Brassicaceae family (Yaniv *et al*., 1999). Here we present a high‐quality chromosome‐level assembly of the ~2 Gb genome of *M. incana* obtained by multiple sequencing techniques. By integrating comparative genomic analyses and genome‐wide methylation data, we aim to address three main objectives: (1) to elucidate the evolutionary relationships and temporal dynamics of the Hesperodae during the early cladogenesis of the core Brassicoideae; (2) to propose a more accurate model of the ancestral genome of the Hesperodae; and (3) to explore the genomic basis of genome obesity in *M. incana* and other Hesperodae species.

## Results

### Genome assembly, assessment and annotation

**Figure 1:**
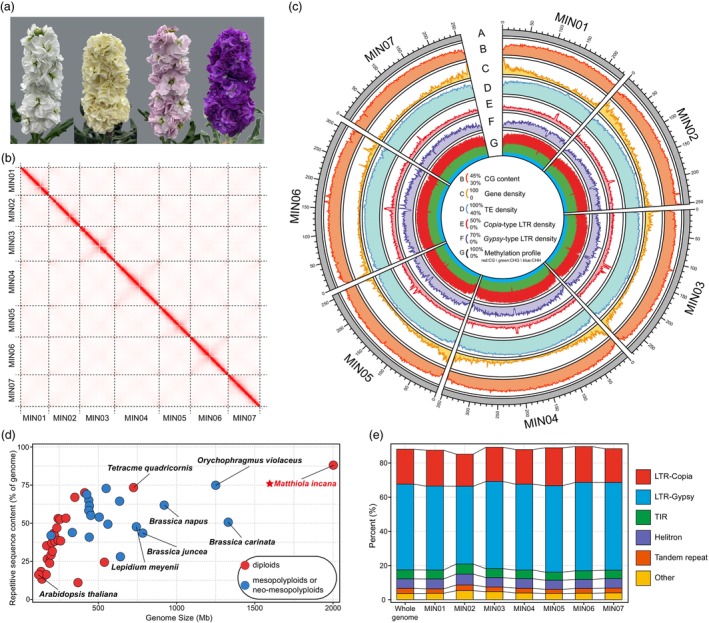
Overview of the *M. incana* genome assembly and its features. (a) Colour diversity in *M. incana*. (b) Genome‐wide chromatin interactions in the Hi‐C contact map. (c) Genomic features of the assembly. From outside to inside rings: chromosome size (A), GC content (B), gene density (C), TE density (D), *Copia*‐type LTR‐RT density (E), *Gypsy*‐type LTR‐RT density (F) and whole‐genome methylation profile (CG: red, CHG: green, CHH: blue). Non‐overlapping window size: 1 Mb. (d) Genome size and repeat content variation of the published Brassicaceae genomes. See Table S5 for details. Red star indicates the species analysed in this study.  (e) Proportion of different repeat types in the whole‐genome assembly and individual pseudochromosomes.

The genome assembly of *M. incana* was constructed by integrating PacBio HiFi reads (49.70 Gb, ~25.0× genome coverage), Illumina short reads (144.01 Gb, ~72.0× genome coverage) and Hi‐C data (415.83 Gb, ~207.5× genome coverage) (Figure S1; Table S1). After assembling with PacBio HiFi reads and polishing with Illumina reads, we obtained a preliminary draft assembly consisting of 791 contigs with a total size of ~1965 Mb, consistent with estimates obtained from 17‐*kmer* and flow cytometric analysis (Figures S2 and S3). The contig N50 was 11.97 Mb (Table S2). The Hi‐C data were used to construct chromosome‐level scaffolds. This resulted in anchoring 310 contigs on seven pseudochromosomes (MIN01 – MIN07), capturing 98.93% (1943.97 Mb) of the genome assembly (Figures 1b,c; Table 1; Tables S3 and S4). The genome assembly of *M. incana* now serves as the largest reference genome of the Brassicaceae family and represents an important milestone in crucifer genomics (Figure 1d; Table S5).

**Table 1:** Statistics of genome assembly and annotation of *Matthiola incana*

Assembly feature	*Matthiola incana*
Estimated genome size (*k* = 21)	2002 Mb
Genome assembly	1964.94 Mb
GC content	40.36%
No. of chromosomes	7
Contig N50	11.97 Mb (Longest: 47.94 Mb)
Scaffold N50	258.27 Mb (Longest: 351.36 Mb)
Repetitive sequence	1730.84 Mb (88.09%)
The number of gene model	38 245
BUSCO	99.6% (S: 96.6%, D: 3.0%)
LTR Assembly Index (LAI)	21.81 (Gold Reference)
Average gene CDS length	1332.55 bp
Average exon number per gene	4.77
Functional annotation	98.33%

Various mainstream methods were applied to assess the quality of the genome assembly. More than 99.94% of the resequencing reads and more than 97.50% of the RNA‐seq reads from the same plant were successfully aligned to the assembly. Additionally, the base error rate of the assembly was found to be <0.003%. The completeness of the genome assembly was evaluated using BUSCO, and 99.63% (1608/1614) of the core eukaryotic genes were detected in the assembly (Table S6). The LTR Assembly Index (LAI) was used to assess continuity and scored 21.81, which is equivalent to the gold quality reference (Ou *et al*., 2018; Figure S4; Table S7). These metrics show that we have successfully obtained a genome reference of *M. incana* with high continuity, high completeness and high base accuracy.

The *M. incana* genome is rich in repetitive sequences, containing 88.09% (1.73 Gb) repetitive sequences, including 71.53% retroelements and 12.88% transposons. Of these, LTR retrotransposons (LTR‐RTs) account for 71.46% of the genome and consist of two major superfamilies: *Copia*‐type LTR‐RTs (19.54%) and *Gypsy*‐type LTR‐RTs (49.84%) (Figures 1c,e; Table 1; Table S8). Compared to other sequenced genomes of Brassicaceae species, the *M. incana* genome has a significantly higher proportion of repetitive sequences, highlighting its unique genomic structure (Table S5; Hloušková *et al*., 2019; Liu *et al*., 2022; Yang *et al*., 2023; Liu *et al*., 2024). Through a combination of *ab initio* prediction, homology search and transcriptome analysis (including RNA‐seq and full‐length transcriptome data), we successfully annotated 38 245 protein‐coding genes, of which 98.95% were accurately anchored on the seven chromosomes. The coding sequence (CDS) of protein‐coding genes was on average 1332 base pairs long and comprised 4.77 exons (Table 1). Within the annotated gene sets, 29 076 (76.03%) high‐confidence gene models were supported by at least one RNA‐seq read. Moreover, 98.35% of all annotated gene models had significant functional annotation matches with various public protein databases (Figure S5; Tables S9 and S10). Furthermore, using full‐length transcriptome data (Iso‐seq), we identified 48 387 transcripts and 4953 (10.24%) gene models that display different splicing patterns. In particular, gene MIN06G2064 (the ortholog of *A. thaliana* AT1G29400), which is involved in the regulation of meiotic division, had 22 alternatively spliced transcripts (Figure S6; Table S11).

### Gene families, phylogenetic relationships and genome collinearity

**Figure 2:**
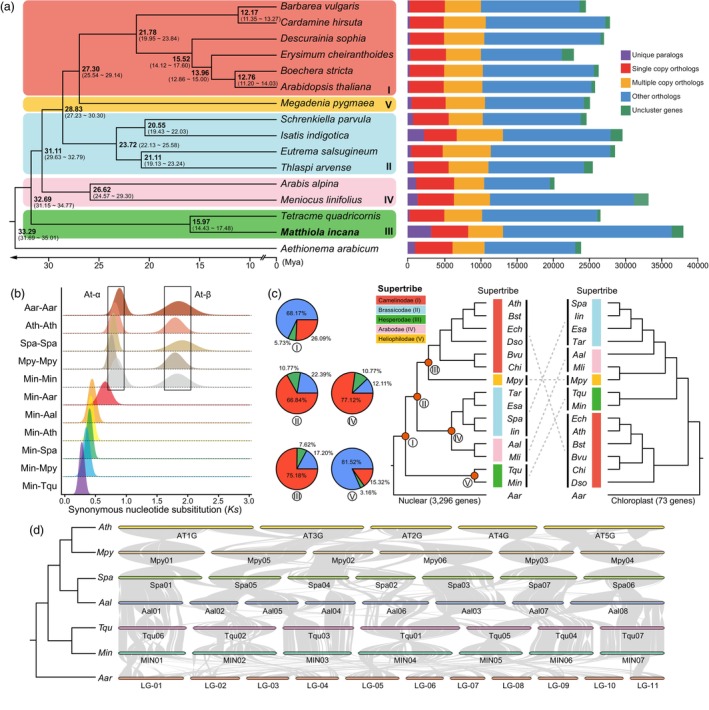
Evolutionary and comparative genomic analyses. (a) The phylogenetic tree with divergence time estimates and the distribution of gene classes in *M. incana* and other 15 Brassicaceae species (right panel). The Roman numerals (I to IV) stand for the supertribes Camelinodae, Brassicaceae, Hesperodae and Arabodae, respectively. (b) *Ks* between collinear genes of *M. incana* and six Brassicaceae species. (c) Extensive conflicts between plastid‐genome‐based and nuclear‐genome‐based species trees. The pie charts represent the supporting relationships between a gene tree and a species tree for the nodes (I – V) within the nuclear‐genome‐based tree (blue: concordant, green: the top alternative bipartition, red: all other alternative bipartitions). (d) Chromosome‐level synteny diagrams showing intergenomic collinearity between *M. incana* and other six Brassicaceae species. Aal, *Arabis alpina*; Aar, *Aethionema arabicum*; Ath, *Arabidopsis thaliana*; Bst, *Boechera stricta*; Bvu, *Barbarea vulgaris*; Chi, *Cardamine hirsuta*; Dso, *Descurainia sophia*; Ech, *Erysimum cheiranthoides*; Esa, *Eutrema salsugineum*; Iin, *Isatis indigotica*; Min, *Matthiola incana*; Mli, *Meniocus linifolius*; Mpy, *Megadenia pygmaea*; Tar, *Thlaspi arvense*; Spa, *Schrenkiella parvula*; Tqu, *Tetracme quadricornis*.

To elucidate the evolutionary relationships of *M. incana*, we conducted a comparative genomics analysis using 16 representative diploid Brassicaceae species (Table S12). A total of 24 683 gene families were identified in 16 Brassicaceae species, with 17 032 gene families detected in *M. incana*, including 687 unique gene families (Table S13). The GO and KEGG enrichment analyses revealed that these unique genes of *M. incana* are involved in diverse biological processes, including ‘regulation of lipid metabolic process (GO:0019216)’, ‘regulation of cell cycle (GO:0051726)’, ‘photorespiration (GO:0009853)’ and many metabolic pathways, such as ‘citrate cycle (TCA cycle)’, ‘pyruvate metabolism’, ‘starch and sucrose metabolism’ (Figures S7 and S8; Tables S14 and S15). A phylogenetic analysis based on 1926 shared single‐copy genes was used to construct a tree mapping the divergence times within the Brassicaceae family (Figure 2a). The results showed that *M. incana* belongs to the supertribe Hesperodae and diverged from *Tetracme quadricornis* about 15.97 million years ago (Mya, confidence interval 14.43–17.48 Mya). The most recent common ancestor of all five supertribes (Arabodae, Brassicodae, Camelinodae, Heliophilodae and Hesperodae) diverged from the Aethionemoideae (*Aethionema arabicum*) approximately 33.29 (31.69–35.01) Mya. The Hesperodae diverged from the common ancestor of Arabodae, Brassiccodae and Heliophilodae ~32.69 (31.15–34.77) Mya. Analysis of synonymous substitution rates (*Ks*) showed that *M. incana*, like the other diploid Brassicaceae species, has undergone two whole‐genome duplications (WGDs). The orthologous *Ks* peak values of *M. incana* compared to *Ae. arabicum*, *Arabis alpina*, *Arabidopsis thaliana*, *Thellungiella parvula*, *Megadenia pygmaea* and *T. quadricornis* are 0.767, 0.444, 0.424, 0.403, 0.352 and 0.278, respectively, confirmed the phylogenetic distances and divergence times inferred from our phylogenetic analysis (Figure 2b).

To further elucidate the evolutionary relationships among the 16 Brassicaceae species, we generated a collinear nuclear gene dataset comprising 3296 homoeologous genes (Figure 2c). Using these collinear genes, we constructed a phylogenetic tree by head‐to‐tail sequence concatenation, which generally corroborated the tree based on single‐copy genes (Figure 2a). However, discrepancies were observed between the gene trees and the species tree, suggesting complex evolutionary dynamics. For each of the 3296 collinear genes, we constructed individual gene trees focusing on key evolutionary nodes for different supertribes and between *M. incana* and *T. quadricornis* (Figure 2c). The congruence between the species tree and the gene tree of the two Hesperodae species, *M. incana* and *T. quadricornis*, was 81.52%, indicating a relatively high concordance. We then assessed the congruence between these gene trees and the species tree. Our results show that 68.17% of the gene trees support the Hesperodae as a sister to Camelinodae, Brassicodae and Arabodae (Figure 2c). Nuclear‐plastid conflict is a common phenomenon in phylogenetic reconstructions in the Brassicaceae (Hendriks *et al*., 2023). We constructed a species tree based on 73 chloroplast genes (Figure 2c). The result is consistent with previous studies. In the chloroplast species tree, the Camelinodae are sister to Arabodae, Brassicodae, Heliophilodae and Hesperodae, rather than the Hesperodae being the sister clade to Arabodae, Brassicodae Camelinodae and Heliophilodae, as shown by nuclear gene analysis. These results support the complex reticulate evolution within the Brassicoideae (Hendriks *et al*., 2023).

To determine the level of intergenomic collinearity between *M. incana* and other crucifer species, we mapped the protein sequences of *M. incana* to six genomes representing different supertribes (German *et al*., 2023) and ancestral karyotypes: *Ae. arabicum* (Aethionemoideae), *A. thaliana* (ACK, Camelinodae), *Ar. alpina* (KAA, Arabodae), *T. parvula* (PCK, Brassicacodae), *M. pygmaea* (Heliophilodae) and *T. quadricornis* (CEK, Hesperodae). The six genomes showed extensive collinearity with *M. incana*, with a 1:1 correspondence between 10 060 (*M. incana* to *Ar. alpina*) and 15 813 (*M. incana* to *T. quadricornis*) collinear gene pairs (Figure 2d; Figures S9–S14).

### Ancestral Hesperodae karyotype (AHK)

**Figure 3:**
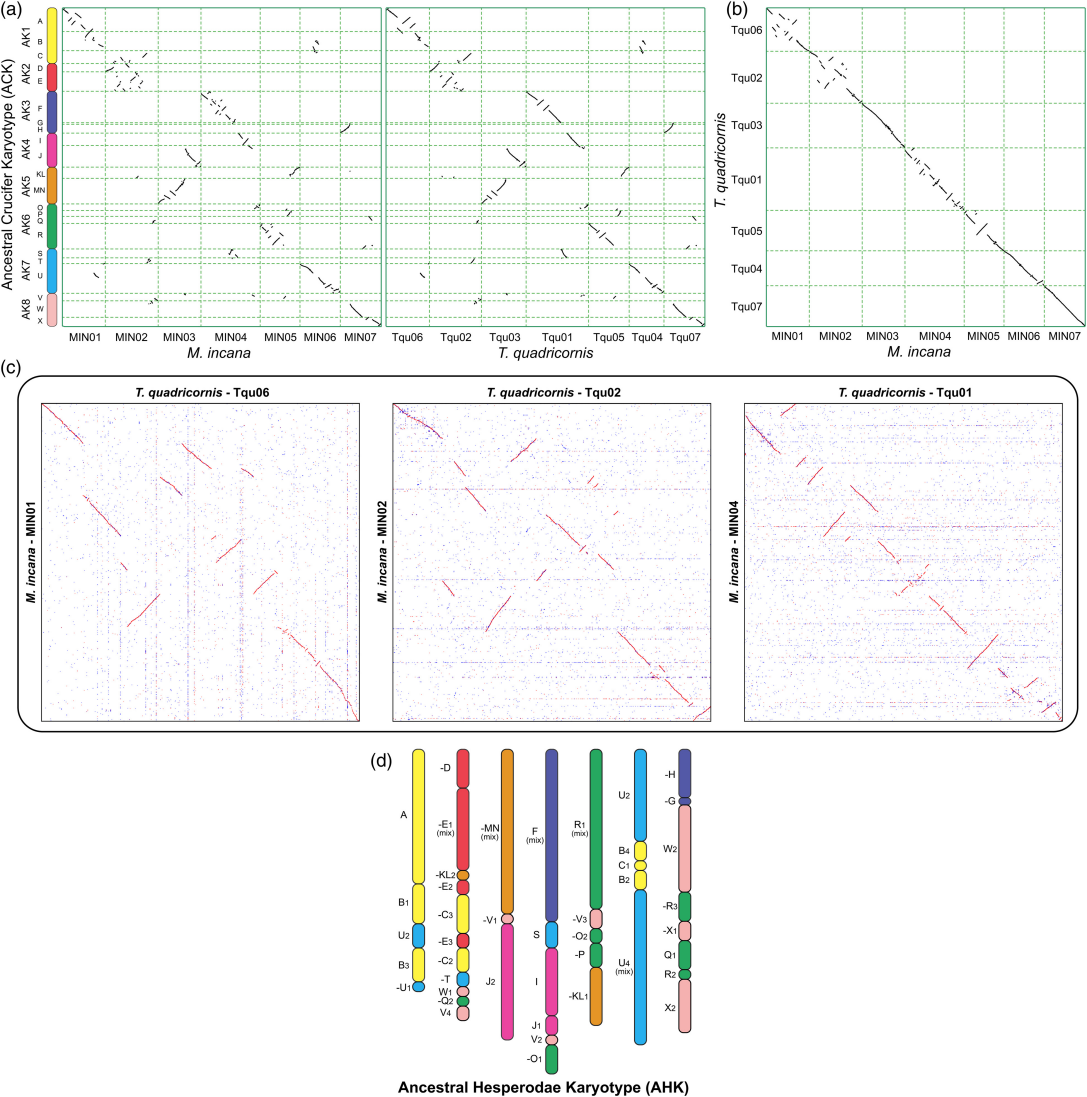
The inference of AHK. (a, b) The homeologous dot plot between *M. incana* and ACK (22 GBs, A‐X), between *T. quadricornis* and ACK (a) and between *M. incana* and *T. quadricornis* (b). (c) The zoom‐in of collinearity dot plots between homeologues MIN01‐Tque06, MIN02‐Tqu02 and MIN04‐Tqu01. (d) The comparative structure of AHK. Colour coding and capital letters correspond to the eight chromosomes and 22 genomic blocks (A‐X) of ACK (Lysak *et al*., 2016). Blocks split into two or more parts are indexed by numbers, and the numbers are related to the gene order within a given GB in ACK (*A. thaliana*). The ‘–’ means that the block in AHK is in the opposite direction to the GB in ACK, and the ‘mix’ means that there are a large number of rearrangements within the one contiguous block, such as the blocks E_1_, MN, F, R_1_ and U_4_.

We compared the genomes of *Aethionema arabicum*, *T. quadricornis* (Euclidieae) and *M. incana* using 22 genomic blocks (GBs, A‐X) of the ACK. First, we identified syntenic chromosomal blocks between *M. incana* and *A. thaliana* by an all‐against‐all BLASTP comparison. Subsequently, the 22 GBs were assigned to the chromosomes of *M. incana* and *T. quadricornis* (Figure 3a). Using a synteny‐based approach, we determined the syntenic blocks and breakpoints between the genomes of *M. incana*, *T. quadricornis* and *Ae. arabicum*. The comparison between *M. incana* and *T. quadricornis* showed a one‐to‐one synteny relationship between the seven chromosomes of both genomes without inter‐chromosomal rearrangements (Figure 3b). The synteny comparison of *M. incana* and *T. quadricornis* uncovered two different situations: (1) intact chromosomal synteny with a minimum of inversions (e.g. MIN03‐Tqu03, MIN06‐Tqu04 and MIN07‐Tqu07); (2) extensive intra‐chromosomal rearrangements (e.g. MIN01‐Tqu06, MIN02‐Tqu02, MIN04‐Tqu01) (Figure 3c). The comparative analysis revealed intra‐chromosomal translocations and inversions in both the MIN01‐Tqu06 and MIN02‐Tqu02 chromosome pairs. Notably, the MIN04‐Tqu01 pair exhibited a markedly higher frequency of inversion events compared to other homeologous chromosome pairs. To investigate the karyotypic alterations in these rearranged chromosomes, we performed detailed comparisons of breakpoints between *M. incana* and *T. quadricornis*, *T. quadricornis* and *Ae. arabicum*, and *M. incana* and *Ae. arabicum*. To recover the ancient continuous blocks in *Ae. arabicum*, we refined the order and orientation of the inferred ancestral chromosomes (Figure S15).

According to the MIN‐Tqu collinearity comparison, we used the breakpoint as the boundary and then compared the genomic segment with *Ae. arabicum* to determine which MIN/Tqu segment has a stronger collinear relationship with the more ancestral *Ae. arabicum* genome. As a result, we inferred the AHK, compared to the CEK previously inferred using cytogenetic analyses (Mandáková *et al*., 2017a), carefully defined the boundaries and associations between the 22 GBs, and identified block T, which was not previously placed (Figure 3d). The AHK differs from the ancestral genomes of the other Brassicaceae supertribes. Based on the comparison with the 22‐GB model, there are many rearrangements within some GBs, especially E, F, R and U. However, the current AHK may need more genomic evidence to be validated. Continued research and additional genomic data will be critical to validate and further refine the inferred ancestral genome.

### Burst of LTR‐RT insertions in the *M. incana* genome

**Figure 4:**
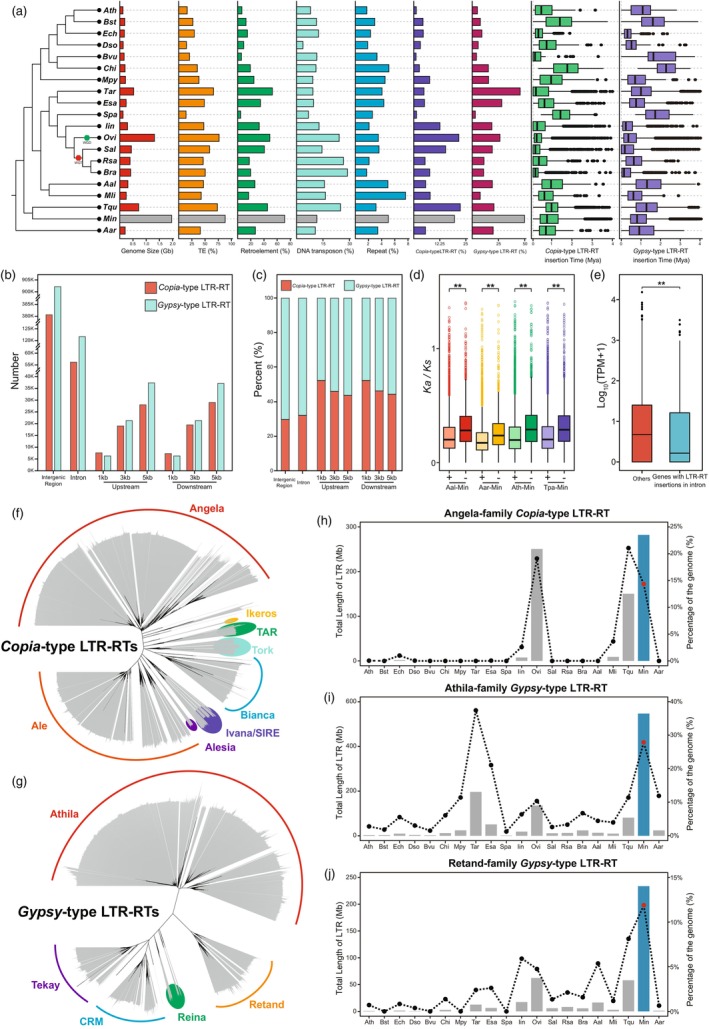
Comparative genomics analysis of LTR‐RTs in the *M. incana* genome. (a) Genome and TE information of *M. incana* and other 19 crucifer species (genome sizes, percentage of TEs, percentage of retroelements, percentage of DNA transposons, percentage of *Copia*‐type LTR‐RTs, percentage of *Gypsy*‐type LTR‐RTs and insertion times of LTR‐RTs). (b, c) The distribution of *Copia*‐ and *Gypsy*‐type LTR‐RTs in the *M. incana* genome. (b) count number; (c) proportion. (d) The *Ka/Ks* value of genes with and without LTR‐RT insertions in gene pairs of several related species. + and – indicates an insertion or its absence. (e) Gene expression levels of genes with and without LTR‐RT insertions in intron. (f, g) The phylogenetic relationships between *Copia*‐ and *Gypsy*‐type LTR‐RT families, respectively. (h–j) The content of different LTR‐RT families in the genome of *M. incana* and 13 other crucifer genomes. (h) Angela‐family (*Copia*); (i) Athila‐family (*Gypsy*); (j) Retand‐family (*Gypsy*). Aal, *Arabis alpina*; Aar, *Aethionema arabicum*; Ath, *Arabidopsis thaliana*; Bra, *Brassica rapa*; Bst, *Boechera stricta*; Bvu, *Barbarea vulgaris*; Chi, *Cardamine hirsuta*; Dso, *Descurainia sophia*; Ech, *Erysimum cheiranthoides*; Esa, *Eutrema salsugineum*; Iin, *Isatis indigotica*; Min, *Matthiola incana*; Mli, *Meniocus linifolius*; Mpy, *Megadenia pygmaea*; Ovi, *Orychophragmus violaceus*; Rsa, *Raphanus sativus*; Sal, *Sinapis alba*; Tar, *Thlaspi arvense*; Spa, *Schrenkiella parvula*; Tqu, *Tetracme quadricornis*.

In the Brassicaceae family, the genome size of most diploid species is <0.5 Gb (Figure 1d). In contrast, the genome size of *M. incana* is approximately 2 Gb, which is considerably larger than that of the other species of Camelinodae and Brassicodae (Lysak *et al*., 2009). To investigate the reasons for this genome expansion, we conducted a detailed comparative analysis including 20 Brassicaceae species (Figure 4a; Table S12). We found that *M. incana* has larger intronic and intergenic regions compared to the other 19 genomes. However, there are no significant differences in the total length of the CDS between these genomes (Figures S16 and S17; Table S16). Thus, the expansion of intronic and intergenic regions appears to be the major contributor to genome enlargement, explaining 23.43% and 73.04% of the size variation, respectively. This expansion is primarily due to the amplification or insertion of transposable elements (TEs), particularly retroelements (Figure 4a). Further analysis revealed an increase in *Copia*‐ and *Gypsy*‐type LTR‐RTs (Figure 4a; Table S17). The average proportion of *Copia* and *Gypsy* LTR‐RTs per Mb in the *M. incana* genome is higher than in other species (Figures S18 and S19). However, the length of intact LTR‐RTs in *M. incana* is not significantly different from those in other species (Figure S20).

Among the different types of TEs in the *M. incana* genome, the number and proportion of LTR‐RTs sequences (~220 million and 71.46%, respectively) are significantly higher than those of the other types of TEs (Figure 4a; Table S5). In *M. incana*, approximately 70% of the intact LTR‐RTs appear to have been amplified within the last one million years and ~95% of the LTR‐RTs within 2 million years (Figure 4a; Figure S21). Approximately 81% of LTR‐RTs are located in intergenic regions and 11% in introns, with ~30% of *Copia* types and ~70% of *Gypsy* types found in both locations. Furthermore, about 2.5% of the LTR‐RTs are located within 3 Kb upstream (5′ of the start codon) and downstream (3′ of the terminator) of the genes. Notably, the number of *Copia* and *Gypsy* increases with increasing distance from the gene (Figure 4b). Additionally, the proportion of *Gypsy* types increases while the proportion of *Copia* types decreases with increasing distance from the gene (Figure 4c). We compared the *Ka*/*Ks* values and expression levels of genes with and without LTR‐RT insertions. The genes with LTR‐RT insertions experienced stronger selection pressure, as evidenced by a smaller *Ka*/*Ks* value (Figure 4d). Additionally, the transcription of genes with LTR‐RT insertions in the intron is considerably lower than that of genes without LTR‐RT insertions (Figure 4e).

We have constructed an evolutionary phylogenetic tree of *Copia*‐ and *Gypsy*‐type LTR‐RTs based on their respective superfamily protein domains. The *Copia* types were divided into eight branches, with the Angela accounting for the largest proportion, 27% of the genome (Figures 4f,h; Figures S22 and S23; Table S18). The *Gypsy* types formed five branches, with the Athila and Retand families accounting for the highest and second highest proportions with 36% and 12% of the total genome, respectively (Figures 4g,j,i; Figures S22 and S23; Table S19). A detailed comparison of the families of intact *Copia* and *Gypsy* elements revealed significant expansions in specific families. Particularly notable was the substantial increase in Ale, Angela, Bianca and TAR families, with Angela showing the largest increase (Figure 4h). Among the *Gypsy* types, Athila and Retand elements underwent significant amplification, while Tekay elements were less amplified (Figure 4i,j).

### Extremely high level of DNA methylation in *M. incana*

**Figure 5:**
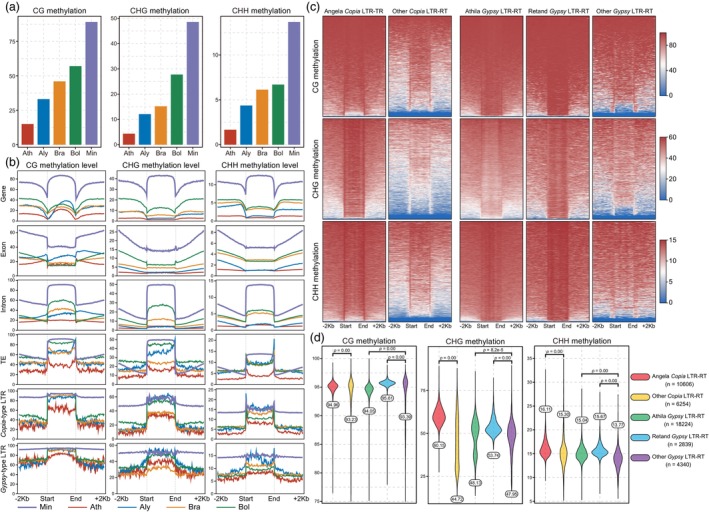
Genome‐wide DNA methylation analysis in *M. incana*. (a) Average DNA methylation levels (%) in CG, CHG and CHH sequence contexts in *M. incana* and other crucifer species. (b) CG, CHG and CHH methylation level (%) in gene, exon, intron, TE, *Copia*‐type and *Gypsy*‐type LTR‐RTs and their flanking regions (2 kb upstream and downstream) in *M. incana* and other crucifer species. (c) Heatmaps of DNA methylation level (%) (CG, CHG and CHH) of *Copia*‐ and *Gypsy*‐type LTR‐RTs. (d) CG, CHG and CHH methylation level (%) of *Copia*‐ and *Gypsy*‐type LTR‐RTs. The statistical analysis was performed using the two‐sided Wilcoxon test. Ath, *Arabidopsis thaliana*; Aly, *A. lyrata*; Bra, *Brassica rapa*; Bol, *B. oleracea*; Min, *M. incana*.

Whole‐genome bisulphite sequencing (WGBS) data were used to explore the levels of DNA methylation in the *M. incana* genome. Compared to the four crucifer species in two supertribes with variable genome size and available WGBS data (*A. thaliana*, *A. lyrata*, *B. rapa* and *B. oleracea*), the methylation levels in *M. incana* are exceptionally high: mCG 89.09%, mCHG 48.56% and mCHH 13.68% (Figure 5a). This increased 5mC is evenly distributed throughout the genome, in contrast to other species where methylation increases mainly in the pericentromeric region (Figure 1c; Figures S24–S28). In *M. incana*, this methylation pattern is related to the overall distribution of TEs across the chromosome length, as methylation often plays a role in silencing these elements, preventing their movement and the potential genomic instability they can cause. Interestingly, one or two regions with extremely low methylation levels were found on each chromosome (Figure 1c). A detailed analysis of these regions showed a significant reduction in CHG methylation, as opposed to CG and CHH methylation, which remained relatively stable (Figure S28). These regions exhibit the highest density of *Copia*‐type LTR‐RTs and the lowest density of *Gypsy*‐type LTR‐RTs (Figure 1c). As centromere‐specific H3‐like protein (CENH3) binds to sequences with reduced DNA methylation (Zhang *et al*., 2008), we hypothesized that the hypomethylated regions correspond to centromeres. Moreover, these regions correspond to putative centromeric regions with the signal intensity lower than the whole‐genome average in the HiC map (Figure 1b). When examining these candidate centromeric regions with notably weak Hi‐C interaction signals at 50‐kb resolution, we found an exceptionally high content of TEs, primarily consisting of expanded *Copia*‐type LTR retrotransposons (Figures S29–S35).

To further understand the impact of methylation on genome elements, we compared the methylation status of genes, exons, introns, TEs, *Copia*‐ and *Gypsy*‐type LTR‐RTs in *M. incana* and the four genomes analysed (Figure 5b). We show that *M. incana* has a higher degree of methylation modification in all regions compared to the other species, especially in genes including intron and exon regions. The average methylation level of TEs is also higher than in the other species, but the differences are not as great as in the gene regions (Figure 5b). This could be due to the fact that a large number of TEs were inserted into gene regions, or more precisely, that these TEs became part of introns. This is consistent with the presence of the most obvious hypermethylation modification in the intron regions. We compared the degree of methylation between the significantly expanded LTR‐RTs (Angela, Athila and Retand) and the remaining LTR‐RTs in *M. incana* (Figure 5c). The results showed that LTR‐RTs that expanded had a higher level of methylation modification than the remaining LTR‐RTs (Figure 5c,d). We found that methylation of LTR‐RTs, whether in the CG, CHG or CHH context, exhibited a progressive decrease in the amplitude of variation with insertion time and eventually stabilized around a constant value (Figure S36; Table S20). This fluctuation may reflect ongoing epigenetic regulation and adaptation processes in the genome.

### Molecular mechanism of colour diversity in *M. incana*

**Figure 6:**
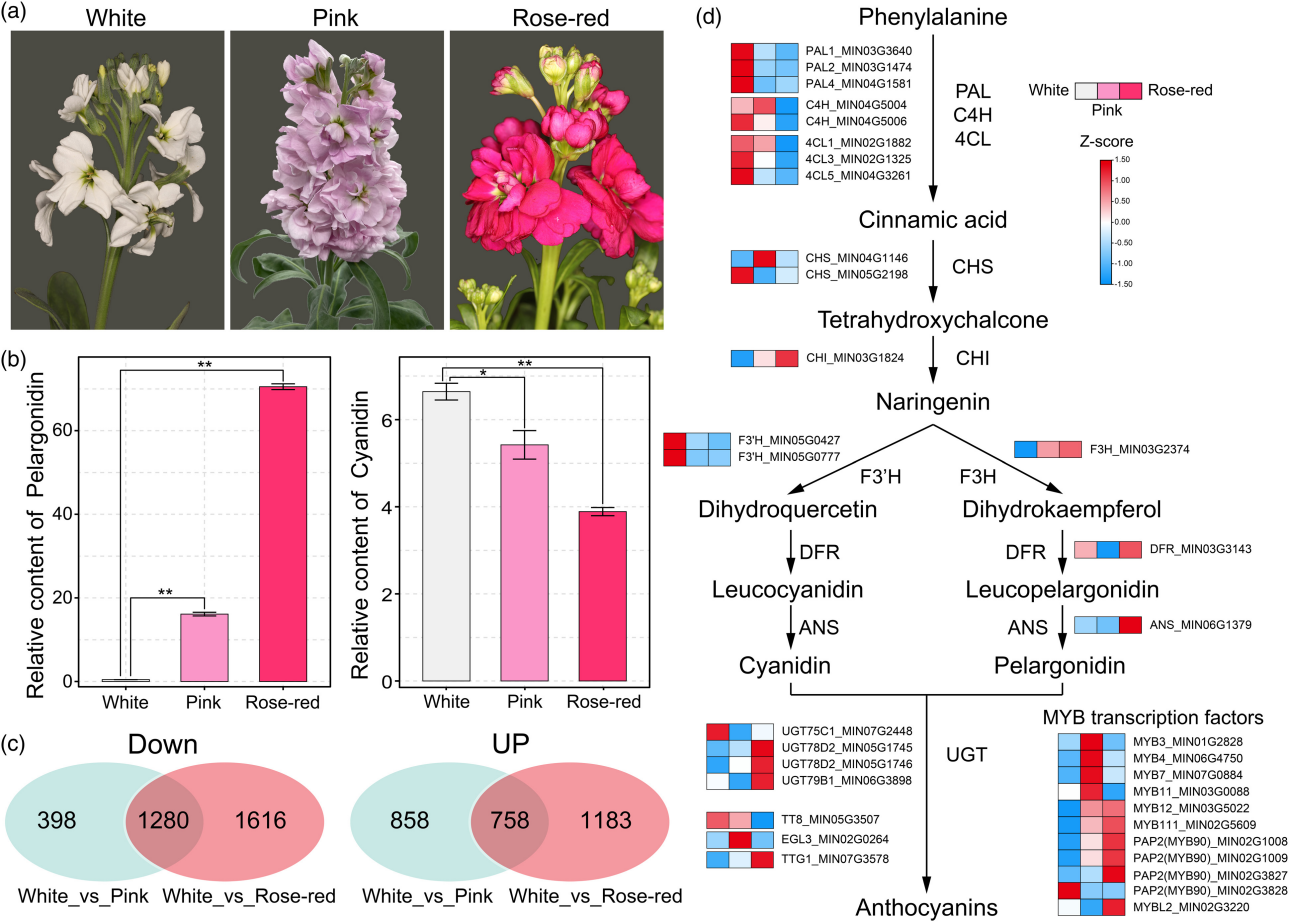
Transcriptome and metabolome analysis of the petals of *M. incana* cultivars with white, pink and rose‐red flowers. (a) Phenotypes of white, pink and rose‐red flower varieties. (b) The content of pelargonidin and cyanidin in white, pink and rose‐red flower varieties. (c) Differentially expressed genes in the transcriptomes of white, pink and rose‐red flower varieties. (d) Proposed synthesis pathway of anthocyanins in *M. incana*.

*M. incana* is typically a winter ornamental plant, with strong cold hardiness, rich colour and a long flowering period. The flowers of three varieties with different flower colour (white, pink and rose‐red) were selected for metabolome and transcriptome analysis, to explore the molecular mechanism of colour change (Figure 6a). The metabolome analysis showed that the colour change from white to pink and rose‐red was due to the increase in pelargonidin content (Figure 6b, Table S21). Comparative transcriptome analysis showed that 1280 genes were co‐down‐regulated and 758 co‐up‐regulated differentially genes in pink/rose‐red varieties compared to the white flower cultivar (Figure 6c, Table S22). The GO and KEGG results of these differentially expressed genes indicated that they were mainly enriched in biosynthetic processes and anthocyanin biosynthesis (Figures S37 and S38).

To further understand the molecular mechanism of pink and rose‐red petal formation in *M. incana*, we identified the genes related to the anthocyanin synthesis pathway in *M. incana* based on the anthocyanin biosynthesis pathway in *A. thaliana* (Table S23). In the transition from white to pink and rose‐red petals, structural genes such as *F3H*, *DFR*, *ANS*, *UGT* and transcriptional regulators such as *MYB11*, *MYB12*, *MYB111*, *MYB90*, *MYBL2* and *TTG1* were up‐regulated (Figure 6d). Combined transcriptome–metabolome analysis revealed that the pathway in which *F3H* catalyses the conversion of naringenin to dihydrokaempferol, subsequently leading to the production of pelargonidin, is the primary route for the accumulation of colourful pigments in *M. incana* (Figures 6b,d). At the same time, we observed that the expression levels of R2R3‐MYB transcription factors, including *MYB12*, *MYB111*, and three *MYB90*s, which can activate the expression of structural genes such as *F3H*, were considerably higher in pink and rose flowers compared with white flowers. These genes could be crucial to explain the colour differences between the three varieties.

## Discussion

Here, we developed a chromosome‐level genome assembly of *M. incana* by integrating long‐read sequences from PacBio HiFi reads with high‐precision short reads from Illumina sequencing. Additionally, we used Hi‐C data for super‐scaffolding to improve the quality of the assembly. This resource not only provides an important reference to study genome and karyotype evolution in the mustard family, but also facilitates the analysis of the genetic basis of interesting traits.

Our phylogenetic analysis confirmed that the Hesperodae are the first diverging clade within the core Brassicaceae. The result is consistent with the recent unparalleled Brassicaceae phylogeny, which revealed that the Hesperodae are sister to the rest of the core Brassicaceae, although relationships among the other major lineages varied depending on the sampling routine and/or phylogenetic approach used (Hendriks *et al*., 2023). However, in the chloroplast species tree (Hendriks *et al*., 2023), the Camelinodae (Clade A) are sister to the Brassicodae, Heliophilodae, Arabodae and Hesperodae (Clades B, C, D and E). Topological incongruence in the tree can result from various biological patterns, such as gene duplications, horizontal gene transfer, incomplete lineage sorting (ILS) or homoploid hybridization or introgression (Wendel and Doyle, 1998). Among these patterns, introgression and ILS are two widely documented mechanisms of phylogenetic discordance that are difficult to distinguish (Duan *et al*., 2023). Recently, Gardner *et al*. (2023) applied an approach to investigate localized gene tree incongruence by simplifying all rooted gene trees to triplets and counting the occurrence frequencies for each of the three possible topologies, indicating that most fig trees (*Ficus*, Moraceae) were demonstrably involved in introgression events. Similarly, the gene trees and species tree were constructed here with 3296 collinear genes and also show incongruence. This suggests that frequent hybridization and introgression events may have occurred during the early evolution and divergence of the Hesperodae and other supertribes.

Mandáková *et al*. (2017a) used comparative chromosome painting analysis on pachytene and mitotic chromosomes of seven Hesperodae species to identify 22 GBs and inferred an ancestral karyotype of clade E (CEK) with seven chromosomes. Through a *de novo* genome assembly, we identified all 22 GBs in the genome of *M. incana*, including the T block that was not detected by chromosome painting (Mandáková *et al*., 2017a). Comparative genomic analysis shows that several GBs were fragmented and rearranged in *M. incana*. The comparative analysis of *T. quadricornis* (Li *et al*., 2024) and *M. incana* revealed that the chromosomes of the two Hesperodae species maintain a very good 1:1 correspondence, especially chromosomes MIN03‐Tqu03, MIN06‐Tqu04 and MIN07‐Tqu07, with only a few inversions. Using these two high‐quality genomes and the *Aethionema arabicum* genome, we inferred a new ancestral karyotype of the Hesperodae (Figure 3d).

In meso‐ and neopolyploid crucifer genomes, chromosomal structural changes can occur due to genomic redundancy and recombination between homeologous sequences and repeats (Cheng *et al*., 2013, 2017; Mandáková *et al*., 2017b; Yang *et al*., 2023). However, cytogenetic analyses have shown that there is no additional whole‐genome duplication (WGD) in *M. incana*, similar to the genome of *A. thaliana* (Mandáková *et al*., 2017a). Here, we confirmed that although *M. incana* shares the At‐*β* and At‐*α* WGDs with all Brassicaceae species, it has not undergone subsequent meso‐ or neopolyploidization, so that the intra‐chromosomal rearrangements cannot be attributed to post‐polyploid diploidization. One of the obvious causes is the activity and proliferation of TEs, which provide a substrate for illegitimate recombination.

Although there is more than 30‐fold variation in genome size in the Brassicaceae, most species have very small genomes, with a mean genome size of 0.62 Gb (Lysak *et al*., 2009). Crucifer species with large genomes are typically either polyploids or diploidized mesopolyploids. However, some diploid Hesperodae species have the largest genomes and the smallest number of chromosomes (*n* = 5–7) (Hloušková *et al*., 2019; Lysak *et al*., 2009; Mandáková *et al*., 2017a). Amplification of repetitive elements is considered an important factor beyond the obesity of plant and animal genomes (Naville *et al*., 2019; Piegu *et al*., 2006). The study based on low‐pass genome sequencing showed that LTR‐RTs occupy 31 to 48% of Hesperodae genomes larger than 1.5 Gb and may play a key role in genome upsizing in the supertribe (Hloušková *et al*., 2019). The assembly of *M. incana* has covered 1.9 Gb of genome sequence, which to our knowledge makes it the largest sequenced genome of the Brassicaceae family. The genome size and proportion of TEs (85%) in *M. incana* are similar to maize (~2.3 Gb), which has 85% of TE sequences (Anderson *et al*., 2019; Haberer *et al*., 2020; Zhao *et al*., 2018).

LTR retrotransposons were found to account for 71.46% of the *M. incana* genome, which is significantly more than the 48.11% determined by low‐pass genome sequencing (Hloušková *et al*., 2019). Compared to other Brassicaceae species, *Gypsy*‐ and *Copia*‐type LTR‐RTs have expanded considerably in the *M. incana* genome over the last 2 million years. In *M. incana*, the proportion of the *Gypsy* retrotransposons (49.84%) is significantly higher than that of the *Copia* retrotransposons (19.54%). The result is consistent with the results of low‐pass genome sequencing in *M. incana* as well as in other six Hesperodae species, where Ty3/*Gypsy* elements were identified as the major repeatome component driving the observed genome expansions in all large‐genome species (Hloušková *et al*., 2019). In plants, the *Ty3*/*Gypsy* and *Ty1*/*Copia* retrotransposon superfamilies are typically the most prevalent repetitive elements. However, the prevalence of one type over the other varies between taxa (Pellicer *et al*., 2018). *Gypsy*‐like LTR‐RTs are also the major TE type in many other Brassicaceae species, such as in *Eutrema salsugineum* (Zhang *et al*., 2020) and *Brassica rapa* (Cai *et al*., 2022). However, in contrast to previous findings that attributed differences in genome size between Brassicaceae species to the expansion of pericentromeres (Hall *et al*., 2006; Willing *et al*., 2015), the *Ty3/Gypsy* elements that largely contributed to the genome size increase in *M. incana* accumulate uniformly across chromosomes (Hloušková *et al*., 2019). Interestingly, the *Ty1/Copia* elements showed substantial enrichment in the putative centromeric regions, while the density of *Ty3/Gypsy* elements was reduced in these regions. It is hypothesized that the predominance of large genomes in the Hesperodae is associated with adaptability to extreme habitats and the selection for biennial or perennial life histories (Hloušková *et al*., 2019).

We found an unusually high level of DNA methylation that was evenly distributed across the genome of *M. incana* and not concentrated in the pericentromeric regions as in crucifer species with smaller nuclear genomes. This could be due to the high proportion of LTR retrotransposons evenly distributed across the chromosomes, except for the most proximal regions (Hloušková *et al*., 2019). Remarkably, CHG methylation decreased in one or two regions on each chromosome, indicating the potential presence of centromeric regions, which is also supported by Hi‐C mapping (Figure 1b and Figure S28). These regions are also associated with lower gene density and higher repeat density. In *A. thaliana*, assembly of the pan‐centromeres revealed that all CENH3‐enriched regions were severely depleted of DNA methylation in the CHG context compared to the flanking pericentromeric heterochromatin and showed a more modest decrease in the CG context despite the structural diversity of the satellites (Wlodzimierz *et al*., 2023). Similarly, in *M. incana*, regions with decreased CHG methylation correspond to centromere regions. Interestingly, the regions with the highest density of *Copia*‐type LTR‐RTs and the lowest density of *Gypsy*‐type LTR‐RTs overlapped. This result is consistent with observations that the centromeres of *B. rapa* are enriched for *Copia* invasions, while pericentromeres show a higher enrichment for Gypsy LTR‐RTs (Zhang *et al*., 2023).

In conclusion, we have successfully assembled the genome of *M. incana*, the second and largest sequenced genome of the Hesperodae supertribe. Our results suggest that the genome obesity in *M. incana* is largely due to the accumulation of LTR retrotransposons. Furthermore, the accumulation of transposons may also be an important cause of the extensive karyotype changes that make up the modern *M. incana* genome.

## Materials and methods

### Plant materials and DNA/RNA extraction

The *M. incana* accession (Min‐w08) used for sequencing in this study were cultivated plants that were collected in the laboratory and self‐pollinated for 8 generations. S_8_ single‐seed descent *M. incana* was cultivated in a greenhouse located at Gannan Normal University in Ganzhou, China, during the 2020–2021 growing season. The young leaf tissue, stem, bud, root, flower and seed samples were collected from the same *M. incana* plant and immediately flash‐frozen in liquid nitrogen. For transcriptome analysis of flower colour diversity, petals were collected from freshly opened flowers exhibiting white, pink and rose‐red colour of different varieties of *M. incana*. Flash‐frozen samples were then stored at −80 °C in liquid nitrogen. High‐quality genomic DNA was extracted from leaves using a modified CTAB method (Allen *et al*., 2006). Total RNA was isolated using the TRIzol Reagent (Invitrogen Life Technologies, Carlsbad, California, USA). RNA purity and integrity were monitored using a NanoDrop 2000 spectrophotometer (NanoDrop Technologies, Wilmington, DE) and a Bioanalyzer 2100 system (Agilent Technologies, Santa Clara, Califofornia, USA). RNA contamination was assessed using a 1.5% agarose gel.

### Whole‐genome sequencing, Hi‐C sequencing and PacBio ISO‐seq sequencing

DNA extracted from young *M. incana* leaves was used to construct a library for sequencing on the Illumina paired‐end high‐throughput sequencing platform NovaSeq 6000 with a read length of 150 bp. The library was generated following the standard library building process.

For the construction of PacBio libraries, young leaf DNA samples were sheared using a Covaris ultrasonicator. Magnetic beads were utilized for the enrichment and purification of large DNA fragments. Stem‐loop sequencing connectors were added to both ends of DNA fragments, and exonucleases were used to remove the fragments that failed to connect. The constructed libraries were sequenced using the PacBio HiFi Sequel II platform with two SMRT cells.

Leaf tissues were collected and utilized for Hi‐C, as described in previous studies (Rao *et al*., 2014). First, the young leaves were immersed in MS buffer containing a 1% formaldehyde solution. Next, the DNA from the leaves was extracted and digested using the *Dpn*II restriction enzyme. An Illumina paired‐end sequencing library was constructed with an insert fragment size of 350 bp and sequenced using the Illumina HiSeq X‐Ten platform.

RNA extracted from leaves, stems, buds, roots, flowers and siliques was used to construct a single SMRTbell library. The SMRTbell template was annealed to a sequencing primer, bound to polymerase and sequenced on the PacBio Sequel platform using the Sequel Binding Kit 3.0 (Pacific Biosciences, Menlo Park, California, USA) with 20‐h movies. The sequences were clustered and refined using iso‐seq (v3.4.0) software (https://github.com/PacificBiosciences/IsoSeq).

### *De novo* genome assembly and contig anchoring

We utilized clean short‐reads to calculate K‐mers (length = 17) to verify the genome size and estimate it (G) using the formula: G = *k*‐mer number/*k*‐mer depth. Hifiasm (v0.14‐r312) (https://hifiasm.readthedocs.io/en/latest/index.html) (Cheng *et al*., 2021) and HiCanu (v2.0) (https://canu.readthedocs.io/en/latest/tutorial.html) (Nurk *et al*., 2020) software were applied to assemble the draft‐contig genome, respectively. To enhance the continuity of the genome, we utilized quickmerge (v0.3) software (https://github.com/mahulchak/quickmerge) (Jung *et al*., 2020) to generate the final genome by merging the genome assemblies obtained from Hifiasm and HiCanu. The assembly was polished using minimap2 (v2.17) (https://github.com/lh3/minimap2) (Li, 2018) and Racon (v1.4.19) (https://yiweiniu.github.io/blog/2018/03/Genome‐assembly‐pipeline‐miniasm‐Racon) (Vaser *et al*., 2017) software in three iterations utilizing long reads. Additionally, BWA (v0.7.17) (https://github.com/lh3/bwa) (Li and Durbin, 2009) and Pilon (v1.23) (https://github.com/broadinstitute/pilon) (Walker *et al*., 2014) software were used with short reads in three iterations.

A pseudo‐chromosome was constructed using Hi‐C data through the Juicer (v1.6) (https://github.com/aidenlab/juicer) (Durand *et al*., 2016) and 3D‐DNA (https://github.com/aidenlab/3d‐dna) (Dudchenko *et al*., 2017) pipeline. The default parameters were used for grouping, ordering and orienting the contigs. The results were polished using the Juicebox Assembly Tools (v1.11.08) (https://github.com/aidenlab/Juicebox) (Durand *et al*., 2016). Validated paired‐end reads were also utilized to calculate inter‐chromosomal interactions for analysing chromosome territories.

To examine the assembly integrity, the long reads were aligned to the assembly using minimap2 and the short reads were aligned to the assembly using BWA. RNA‐seq data were also aligned back to the genome to verify the quality of the genome assembly using Hisat2 (v2.1.0) (https://daehwankimlab.github.io/hisat2) (Kim *et al*., 2019). The quality of the assembled genome was assessed using Benchmarking Universal Single‐Copy Orthologs (BUSCO, v4.1.4) (https://busco.ezlab.org) (Simão *et al*., 2015) with Embryophyta OrthoDB v10 (Creation date: 2020‐09‐10, number of BUSCOs:1614). The LTR_FINDER (v1.07) (https://github.com/xzhub/LTR_Finder) (Xu and Wang, 2007) and LTRharvest (v1.5.10) (http://genometools.org/) (Ellinghaus *et al*., 2008) software packages were utilized to identify and annotate LTRs in the assembly. These results were integrated and used to calculate the LAI with LTR_retriever (v2.9.0) (https://github.com/oushujun/LTR_retriever) (Ou *et al*., 2018; Ou and Jiang, 2018).

### Annotation of repetitive sequences

Prior to gene prediction, annotation of the genome repeat sequences was performed using EDTA (v1.9.4) pipeline (https://github.com/oushujun/EDTA) (Ou *et al*., 2019). The first step involved conducting *de novo* identification of repetitive elements, where individual TE libraries were constructed with parameters ‘‐species others ‐‐sensitive 1 ‐‐anno 1 ‐‐evaluate 1’. These libraries served as crucial inputs for the subsequent step of structural annotation of the TEs. We utilized RepeatMasker (v4.1.1) software (http://repeatmasker.org) (Price *et al*., 2005) with parameters ‘‐e rmblast ‐s ‐gff ‐nolow ‐no_is ‐norna’ to mask the whole‐genome sequences using the TE library constructed by EDTA. Gene predictions were subsequently made based on the masked genomic sequences.

### Gene prediction and functional annotation

We predicted protein‐coding genes using three methods: *ab initio* gene prediction, homologous prediction and transcriptome‐assisted gene prediction. We utilized the Augustus (v3.3.3) software (https://github.com/Gaius‐Augustus/Augustus) (Stanke *et al*., 2004) to perform *ab initio* gene prediction. The models used for each gene predictor were trained using a set of high‐quality proteins generated from the ISO‐seq dataset. To predict homologous genes, we downloaded reference protein sequences of *Aethionema arabicum*, *Arabidopsis lyrata*, *A. thaliana*, *Brassica oleracea*, *B. rapa*, *Schrenkiella parvula* and *Isatis indigotica* (Table S11). We utilized Exonerate (v2.2.0) software (https://www.ebi.ac.uk/about/vertebrate‐genomics/software/exonerate) (Slater and Birney, 2005) for conducting homology‐based gene prediction. For transcriptome‐assisted gene prediction, we utilized the PASA (v2.4.1) pipeline (https://github.com/PASApipeline/PASApipeline) (Haas *et al*., 2008) to align the transcripts to the assembled genome, enabling ORF and gene prediction. A consensus gene set was generated by merging all predictions from the three methods using EvidenceModeler (v1.1.1) software (https://github.com/EVidenceModeler/EVidenceModeler) (Haas *et al*., 2008). To obtain the untranslated regions and alternatively spliced isoforms, we utilized the PASA pipeline to update the gff3 file and generate the final gene models.

Gene functions were inferred by aligning sequences to the National Center for Biotechnology Information (NCBI) Non‐Redundant (NR) and UniProt (Swiss‐Prot and TrEMBL) protein databases using Diamond (v2.0.7.145) (https://github.com/bbuchfink/diamond) (Buchfink *et al*., 2015) with an E‐value of 1e‐5. Predicted protein sequences were then uploaded to KAAS (https://www.genome.jp/tools/kaas/) (Moriya *et al*., 2007) to obtain the Kyoto Encyclopedia of Genes and Genomes (KEGG) annotation, and to EggNOG (http://eggnog‐mapper.embl.de/) (Huerta‐Cepas *et al*., 2019) to obtain additional information. The protein domains were annotated using HMMER (v3.3.1) (http://hmmer.org/) (Mistry *et al*., 2013) software based on Pfam databases with an E‐value of 1e‐5. Gene ontology (GO) annotations for each gene were obtained from InterProScan (v5.50–84.0) (https://github.com/ebi‐pf‐team/interproscan) (Zdobnov and Apweiler, 2001) software.

### LTR retrotransposon analysis

The EDTA pipeline was used to identify all transposable elements (TEs) in the genome of *M. incana* and 19 other related genomes. The results of TE divergence and LTR insertion time were obtained from the intermediate files of the EDTA pipeline. The DeepTE (version 3.7.1) software (https://github.com/LiLabAtVT/DeepTE) (Yan *et al*., 2020) was utilized to accurately identify the unknown‐type LTRs that were identified in the EDTA pipeline. This software was also used to capture the domains of intact *Copia*‐type and *Gypsy*‐type LTRs, respectively. The translated amino acid sequences of domains were used to construct phylogenetic trees. The amino acid sequences targeted from the *Copia* and *Gypsy* superfamilies were aligned using MUSCLE (v3.8.1551) software (http://www.drive5.com/muscle) (Edgar, 2004). Phylogenetic trees for *Copia*‐like and *Gypsy*‐like LTR‐RTs were constructed using IQtree (v2.0.3) software (http://www.iqtree.org/) (Chernomor *et al*., 2016). Furthermore, the undamaged LTRs of *Copia*‐type and *Gypsy*‐type were further classified into more specific clades using TEsorter (v1.3) software (https://github.com/zhangrengang/TEsorter/) (Zhang *et al*., 2022).

### Phylogenetic analysis and divergence time estimation

We used OrthoFinder (v2.5.2) (https://github.com/davidemms/OrthoFinder) (Emms and Kelly, 2019) along with Diamond software to identify common gene families among *Barbarea vulgaris*, *Cardamine hirsuta*, *Descurainia sophia*, *Erysimum cheiranthoides*, *Boechera stricta*, *Arabidopsis thaliana*, *Megadenia pygmaea*, *Schrenkiella parvula*, *Isatis indigotica*, *Eutrema salsugineum*, *Thlaspi arvense*, *Arabis alpina*, *Meniocus linifolius*, *Tetracme quadricornis*, *Aethionema arabicum* and *M. incana* (Table S11). Based on the protein sequences of 1926 single‐copy orthologous families, we used MUSCLE software to align the protein and corresponding coding DNA sequences. The phylogenetic relationship among these species was estimated using RAxML (v8.2.12) software (https://github.com/stamatak/standard‐RAxML) (Stamatakis, 2014). The maximum likelihood method was employed, and a bootstrap value of 1000 was used. Divergence times were estimated using the MCMCtree program, which is part of the PAML (v4.9) package developed by Yang (2007) (http://abacus.gene.ucl.ac.uk/software/paml.html). The time correction points were from the TimeTree (http://www.timetree.org/) website (*A. thaliana* – *B. stricta*: 13.8 ~ 17.0 Mya, *E. salsugineum* – *T. arvense*: 18.6 ~ 23.3 Mya, *Ar. alpina – M. linifolius*: 22.3 ~ 27.6 Mya, *S. parvula – I. indigotica*: 19.8 ~ 24.4 Mya, *C. hirsuta* – *B. vulgaris*: 11.5 ~ 14.2 Mya and *A. thaliana* – *Aa. arabicum*: 27.5 ~ 33.9 Mya). The operating parameters for MCMCTree were set as follows: burn‐in = 10,000, sample size = 50,000 and sample frequency = 10.

### Gene‐species trees conflict analysis

We used JCVI (https://github.com/tanghaibao/jcvi) (Tang *et al*., 2024) software to perform collinearity analysis between 15 genomes and *M. incana*, and obtained 3296 collinear gene sets. We used MUSCLE software to align the protein sequences. Then, the individual gene/orthologue alignments were concatenated to infer phylogenetic relationship among these species using RAxML software. To examine patterns of gene‐species trees conflict, we used the JAVA program PhyParts (v0.0.1) (https://bitbucket.org/blackrim/phyparts/src/master/) (Smith *et al*., 2015), which maps gene trees onto an input species tree and summarizes the number of gene trees in concordance or conflict with each bipartition. Then, we used the python script ‘phypartspiecharts.py’ (https://github.com/mossmatters/phyloscripts/tree/master/phypartspiecharts) to visualize the PhyParts results.

### Chloroplast assembly and phylogenetic reconstruction

To investigate maternal phylogenetic relationships, *de novo* assembly of the chloroplast was carried out using Oatk (v1.0) software (https://github.com/c‐zhou/oatk) with the cleaned PacBio HiFi reads. The chloroplast genome sequences of other species used in this study were downloaded from NCBI (National Center for Biotechnology Information, https://www.ncbi.nlm.nih.gov/) and CGIR (Chloroplast Genome Information Resource, https://ngdc.cncb.ac.cn/cgir/). The gene annotation of all chloroplast assemblies was performed using Geseq software (https://chlorobox.mpimp‐golm.mpg.de/geseq.html) (Tillich *et al*., 2017). We also used JCVI software to perform collinearity analysis between chloroplast genomes and obtained 73 collinear gene sets. Based on these 73 collinear gene sets, we used MUSCLE software to align the protein‐coding sequences (CDS). The phylogenetic relationship among these species was estimated using RAxML.

### Analysis of whole‐genome duplication and genome synteny

To investigate the occurrence of whole‐genome duplication (WGD) in *M. incana*, we conducted an all‐to‐all search in Diamond with an E‐value of 1e‐5 to extract all homologous proteins between *M. incana* and five representative species (*A. thaliana*, *Ae. arabicum*, *S. parvula*, *M. pygmaea* and *Ar. alpina*). We used the WGDI (version 0.4.7) software (https://wgdi.readthedocs.io/en/latest/index.html) (Sun *et al*., 2022a) to extract collinearity blocks, estimate the non‐synonymous (*Ka*) and synonymous (*Ks*) substitution rates and generate a *Ks* distribution map.

To investigate the degree of collinearity, we utilized the JCVI package. We identified syntenic blocks and gene pairs between the six aforementioned species using protein sequences. The collinearity dot plot and collinearity line plot were also generated using the JCVI package.

### RNA sequencing and transcriptome analysis

RNA‐seq libraries were prepared using the TruSeq RNA Sample Preparation Kit (Illumina, San Diego, CA). To select cDNA fragments that are approximately 200 bp in length, the library fragments underwent purification using the AMPure XP system (Beckman Coulter, Beverly, CA). DNA fragments with ligated adaptor molecules on both ends were selectively enriched using the Illumina PCR Primer Cocktail in a 15‐cycle PCR reaction. The products were purified using the AMPure XP system and quantified using the Agilent High Sensitivity DNA assay on a Bioanalyzer 2100 system (Agilent). The sequencing library was sequenced on the Illumina HiSeq NovaSeq 6000 platform.

We used Trimmomatic (v0.39) software (http://www.usadellab.org/cms/index.php?page=trimmomatic) (Bolger *et al*., 2014) to trim the paired‐end reads, removing adaptors and low‐quality reads. Additionally, we removed reads with a trimmed size of less than 100 bp. We aligned the clean reads to the *de novo* assembled genome using Hisat2 with the default settings. The StringTie (v2.1.5) software (https://github.com/gpertea/stringtie) (Kovaka *et al*., 2019) was used to calculate the read counts and gene expression levels in TPM (transcripts per million).

### Bisulphite‐treated DNA sequencing and DNA methylation analysis

Genomic DNA was extracted using a DNeasy plant mini kit according to the manufacturer's instructions (QIAGEN, 69104). Bisulphite conversion of DNA was conducted using the EZ DNA Methylation‐Gold™ kit (ZYMO, D5005), and the bisulphite‐treated DNA libraries were constructed using the Illumina TruSeq DNA sample prep kit, following the manufacturers' instructions. Briefly, 2 μg of DNA for each sample was fragmented into 200–400‐bp pieces by sonication in a Bioruptor (Diagenode). The fragmented DNA was processed by end‐repair, 3′‐end adenylation and adapter ligation. The adapter‐ligated DNA fragments were treated with bisulphite, followed by 8 cycles of PCR amplification. Finally, the bisulphite‐treated PCR products were purified using AMPure XP beads. The paired‐end sequencing of bisulphite‐treated DNA libraries was performed using an Illumina NovaSeq platform.

The sequence quality of the whole‐genome bisulphite sequencing (WGBS) libraries was evaluated using FastQC (v0.11.9) (https://www.bioinformatics.babraham.ac.uk/projects/fastqc/), and the adapter sequences and low‐quality reads were filtered using Trimmomatic. The cleaned reads were then mapped to the reference genomes using BatMeth2‐align (https://github.com/GuoliangLi‐HZAU/BatMeth2) (Zhou *et al*., 2019) with default parameters. DNA methylation calling was performed with BatMeth2‐calmeth with default parameters, and the SAM file was converted to the BAM format with SAMtools. Then we used BatMeth2‐Meth2BigWig to generate BigWig files for IGV visualization.

### Anthocyanin gene identification

The anthocyanin‐related genes were obtained by reciprocal blastp with *A. thaliana* proteins (https://www.arabidopsis.org/) with e‐value <1e‐10, coverage >30% and identity >50%.

### Metabolite extraction, qualitative and quantitative analyses

Petals were collected from freshly opened flowers with white, pink and rose‐red colour of different *M. incana* varieties and freeze‐dried by vacuum freeze‐dryer (Scientz‐100F). The freeze‐dried sample was crushed using a mixer mill (MM 400, Retsch) with a zirconia bead for 1.5 min at 30 Hz. Dissolve 100 mg of lyophilized powder with 1.2 mL 70% methanol solution, vortex 30 s every 30 min for 6 times in total, and place the sample in a refrigerator at 4 °C overnight. Following centrifugation at 12000 rpm for 10 min, the extracts were filtrated (SCAA‐104, 0.22 μm pore size; ANPEL, Shanghai, China) before UPLC‐MS/MS analysis. The sample extracts were analysed using a UPLC‐ESI‐MS/MS system (UPLC, SHIMADZU Nexera X2; MS, Applied Biosystems 4500 Q TRAP).

## Conflict of interest

The authors declare that the study was conducted in the absence of any commercial or financial relationships that could be envisaged and/or construed as a conflict of interest.

## Author contributions

DC, CT, XG and MAL conceived and designed the experiments. HC, SW, ZL, CL and CT prepared the materials, collected the samples and performed the experiments. TY, XZ and FH analysed all the genomic data. HJ, LY and BL provided valuable advice for the experimental design and optimization. TY, GK, MAL and XG wrote the manuscript. All authors approved the manuscript.

## Supporting information


**Figure S1** The diagram for length distribution of Circular Consensus Sequence (CCS) PacBio long reads for *M. incana*.
**Figure S2** The *K‐mer* (k = 17) frequency distribution curve of Illumina short reads (clean data) of *M. incana* genome.
**Figure S3** Flow cytometric genome size estimate in *M. incana* (red) with *Brassica napus* (black) as a reference genome (genome size of *B. napus*: ~1050 Mb).
**Figure S4** Whole‐ genome chromosome‐level LAI (LTR Assembly Index) score distribution in *M. incana*, representing the quality of whole‐ genome assembly.
**Figure S5** The number of genes annotated in *M. incana* using different databases (Swiss‐prot, GO, KEGG, Pfam and Ath_TAIR10).
**Figure S6** The structure of gene MIN06G2064 (an ortholog of AT1G29400) in *M. incana* with the most (22) alternative splicing transcripts.
**Figure S7** The GO enrichments results of unique genes in *M. incana*.
**Figure S8** The KEGG enrichment results of unique genes in *M. incana*.
**Figure S9** The gene level synteny relationship between *Ae. arabicum* and *M. incana* based on 12,773 gene pairs and 1:1 syntenic pattern.
**Figure S10** The gene level synteny relationship between *A. thaliana* and *M. incana* based on 16,001 gene pairs and 1:1 syntenic pattern.
**Figure S11** The gene level synteny relationship between *Ar. alpina* and *M. incana* based on 10,127 gene pairs and 1:1 syntenic pattern.
**Figure S12** The gene level synteny relationship between *T. parvula* and *M. incana* based on 14,597 gene pairs and 1:1 syntenic pattern.
**Figure S13** The gene level synteny relationship between *Megadenia pygmaea* and *M. incana* based on 14,040 gene pairs and 1:1 syntenic pattern.
**Figure S14** The gene level synteny relationship between *T. quadricornis* and *M. incana* based on 16,149 gene pairs and 1:1 syntenic pattern.
**Figure S15** The homologous dot‐plots of all seven chromosome pairs (MIN01‐Tqu06, MIN02‐Tqu02, MIN03‐Tqu03, MIN04‐Tqu01, MIN05‐Tqu05, MIN06‐Tqu04 and MIN07‐Tqu07) with major differences in genomic structure detected by collinearity analysis between *M. incana*, *T. quadricornis*, *Ae. arabicum* and ACK.
**Figure S16** Average length of exons, genes, introns and intergenic regions in *M. incana* and other 19 crucifer species.
**Figure S17** Total length of exons, genes, introns and intergenic regions in *M. incana* and other 19 crucifer species.
**Figure S18** The length of *Copia*‐ and *Gypsy*‐type LTR‐RTs per Mb in *M. incana* and other 19 species.
**Figure S19** The number of *Copia*‐ and *Gypsy*‐type LTR‐RTs per Mb in *M. incana* and other 19 species.
**Figure S20** The length of intact *Copia‐* and *Gypsy*‐type LTR‐RTs in *M. incana* and other 19 species.
**Figure S21** The insertion time of *Copia*‐ and *Gypsy*‐type LTR‐RTs.
**Figure S22** The number of clades of *Copia‐* and *Gypsy*‐type LTR‐RTs in *M. incana* and other 19 species.
**Figure S23** The length of clades of *Copia‐* and *Gypsy*‐type LTR‐RTs in *M. incana* and other 19 species.
**Figure S24** Whole genome DNA methylation (CG, CHG and CHH) in *A. thaliana*.
**Figure S25** Whole genome DNA methylation (CG, CHG and CHH) in *A. lyrata*.
**Figure S26** Whole genome DNA methylation (CG, CHG and CHH) in *B. rapa*.
**Figure S27** Whole genome DNA methylation (CG, CHG and CHH) in *B. oleracea*.
**Figure S28** Whole genome DNA methylation (CG, CHG and CHH) in *M. incana*.
**Figure S29** The chromatin interactions in the Hi‐C contact map and the distribution of TEs and tandem repeats (TR) on chromosome MIN01.
**Figure S30** The chromatin interactions in the Hi‐C contact map and the distribution of TEs and tandem repeats (TR) on chromosome MIN02.
**Figure S31** The chromatin interactions in the Hi‐C contact map and the distribution of TEs and tandem repeats (TR) on chromosome MIN03.
**Figure S32** The chromatin interactions in the Hi‐C contact map and the distribution of TEs and tandem repeats (TR) on chromosome MIN04.
**Figure S33** The chromatin interactions in the Hi‐C contact map and the distribution of TEs and tandem repeats (TR) on chromosome MIN05.
**Figure S34** The chromatin interactions in the Hi‐C contact map and the distribution of TEs and tandem repeats (TR) on chromosome MIN06.
**Figure S35** The chromatin interactions in the Hi‐C contact map and the distribution of TEs and tandem repeats (TR) on chromosome MIN07.
**Figure S36** The relationship between the LTR‐RT insertion time and methylation modifications (mCG, mCHG, mCHH) in *M. incana*.
**Figure S37** GO enrichments results of co‐DEGs.
**Figure S38** KEGG enrichment results of co‐DEGs.

**Table S1** The summary statistics of PacBio HiFi long‐read resequencing and HiC short reads for the final genome assembly in *M. incana*.
**Table S2** The summary statistics Contigs for the final genome assembly in *M. incana*.
**Table S3** The summary statistics Scaffolds for the final genome assembly in *M. incana*.
**Table S4** The statistics of chromosome size of *M. incana*.
**Table S5** The content of repeat sequences in 44 released genomes of species in Brassicaceae.
**Table S6** Evaluation of the genome assembly of *M. incana* using BUSCO.
**Table S7** Evaluation of the genome assembly of *M. incana* using LAI pipeline.
**Table S8** The summary of transposable elements in *M. incana* by EDTA pipeline.
**Table S9** The summary of protein‐coding gene annotation in *M. incana*.
**Table S10** The summary of protein‐coding gene annotation in *M. incana*.
**Table S11** The summary of different transcripts of protein‐coding genes in *M. incana*.
**Table S12** List of plant genome sequences used in the comparative genomic analysis.
**Table S13** Gene families clustered by OrthoFinder in 16 species. Genes used for OrthoFinder were proteins without splice variants.
**Table S14** GO enrichments results of unique genes in *M. incana*.
**Table S15** KEGG enrichments results of unique genes in *M. incana*.
**Table S16** Genomic information of *M. incana* and other 19 genomes.
**Table S17** The summary of length (bp) of transposable elements in *M. incana* and other 19 genomes by EDTA pipeline.
**Table S18** The content (intact count, intact length (Mb), all length (Mb) and percentage of whole genome) of clades of Copia‐type LTR‐RTs in *M. incana* and other 19 species.
**Table S19** The content (intact count, intact length (Mb), all length (Mb) and percentage of whole genome) of clades of Gypsy‐type LTR‐RTs in *M. incana* and other 19 species.
**Table S20** The relationship between the methylation degree and the insertion time of LTR‐RTs in *M. incana*.
**Table S21** The content of anthocyanin metabolites in *M. incana* (white, peach and rose).
**Table S22** The DEGs of White_vs_Peach and White_vs_Rose and gene expresion (TPM) in *M. incana*.
**Table S23** Expression of anthocyanin pathway related genes in *M. incana*.

## Data Availability

All the raw sequencing data generated during the current study are available in the NCBI BioProject under accession number PRJNA883049. The genome fasta and gff3 files were uploaded to Figshare (https://doi.org/10.6084/m9.figshare.21502197.v1). The BS‐seq data of *Arabidopsis thaliana*, *A. lyrata*, *Brassica rapa* and *B. oleracea* were downloaded from NCBI BioProject under accession number PRJNA360049 and PRJNA769222.
