# Supplementary material for: Largest genome assembly in Brassicaceae: retrotransposon‐driven genome expansion and karyotype evolution in Matthiola incana
Source: Plant Biotechnol J. 2025 Jun 26;23(9):4109–25. doi: 10.1111/pbi.70193 (PMC12392961; doi:10.1111/pbi.70193)
Supplement: Supplementary file 1 — Figure S1 The diagram for length distribution of Circular Consensus Sequence (CCS) PacBio long reads for M. incana. Figure S2 The K‐mer (k = 17) frequency distribution curve of Illumina short reads (clean data) of M. incana genome. Figure S3 Flow cytometric genome size estimate in M. incana (red) with Brassica napus (black) as a reference genome (genome size of B. napus: ~1050 Mb). Figure S4 Whole‐ genome chromosome‐level LAI (LTR Assembly Index) score distribution in M. incana, representing the quality of whole‐ genome assembly. Figure S5 The number of genes annotated in M. incana using different databases (Swiss‐prot, GO, KEGG, Pfam and Ath_TAIR10). Figure S6 The structure of gene MIN06G2064 (an ortholog of AT1G29400) in M. incana with the most (22) alternative splicing transcripts. Figure S7 The GO enrichments results of unique genes in M. incana. Figure S8 The KEGG enrichment results of unique genes in M. incana. Figure S9 The gene level synteny relationship between Ae. arabicum and M. incana based on 12,773 gene pairs and 1:1 syntenic pattern. Figure S10 The gene level synteny relationship between A. thaliana and M. incana based on 16,001 gene pairs and 1:1 syntenic pattern. Figure S11 The gene level synteny relationship between Ar. alpina and M. incana based on 10,127 gene pairs and 1:1 syntenic pattern. Figure S12 The gene level synteny relationship between T. parvula and M. incana based on 14,597 gene pairs and 1:1 syntenic pattern. Figure S13 The gene level synteny relationship between Megadenia pygmaea and M. incana based on 14,040 gene pairs and 1:1 syntenic pattern. Figure S14 The gene level synteny relationship between T. quadricornis and M. incana based on 16,149 gene pairs and 1:1 syntenic pattern. Figure S15 The homologous dot‐plots of all seven chromosome pairs (MIN01‐Tqu06, MIN02‐Tqu02, MIN03‐Tqu03, MIN04‐Tqu01, MIN05‐Tqu05, MIN06‐Tqu04 and MIN07‐Tqu07) with major differences in genomic structure detected by collinearity analysis between M. incana, [file PBI-23-4109-s002.pdf]

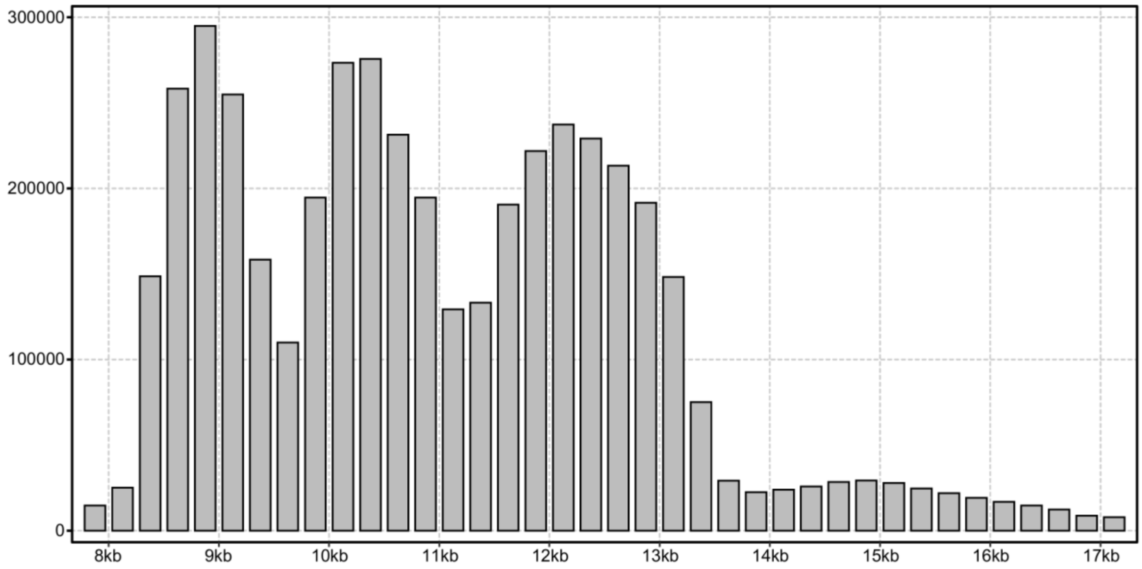

Supplementary Figure 1: The diagram for length distribution of Circular Consensus Sequence (CCS) Pacbio long reads for *M. incana*.

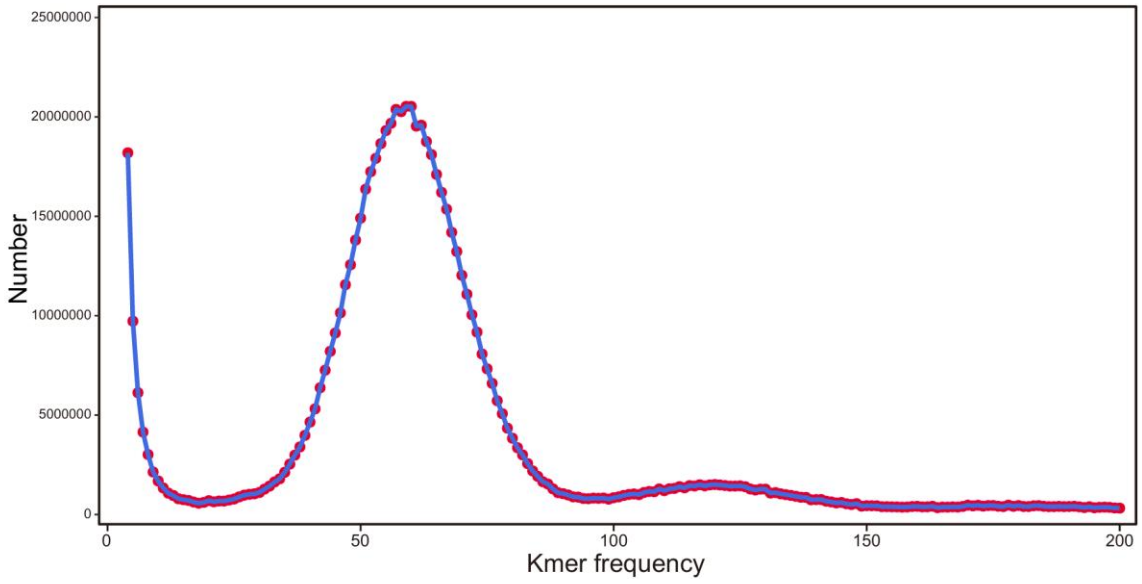

Supplementary Figure 2: The *K-mer* (k =17) frequency distribution curve of Illumina short-reads (clean data) of *M. incana* genome.

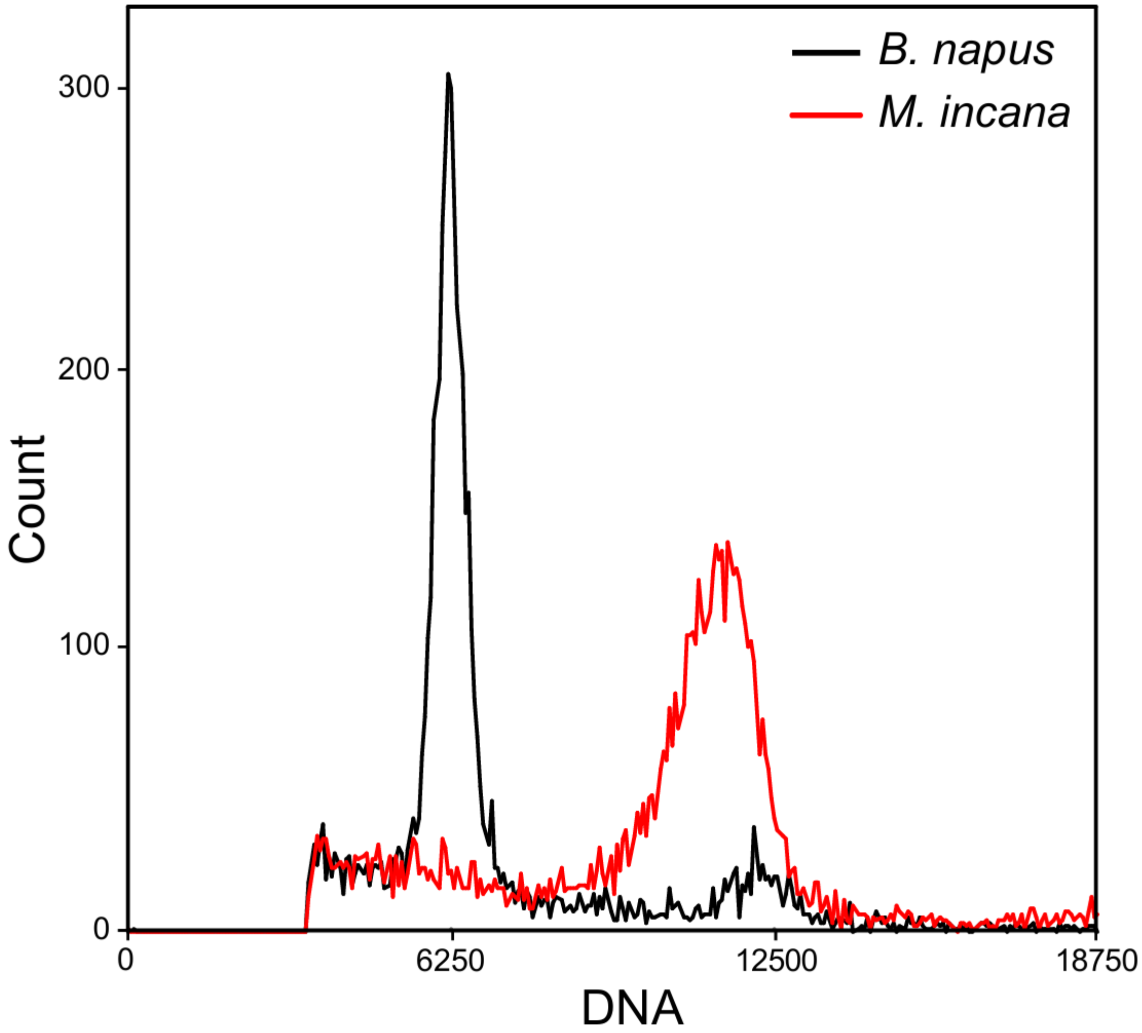

Supplementary Figure 3: Flow cytometric genome size estimate in *M. incana* (red) with *Brassica napus* (black) as a reference genome (genome size of *B. napus*: ~1,050 Mb).

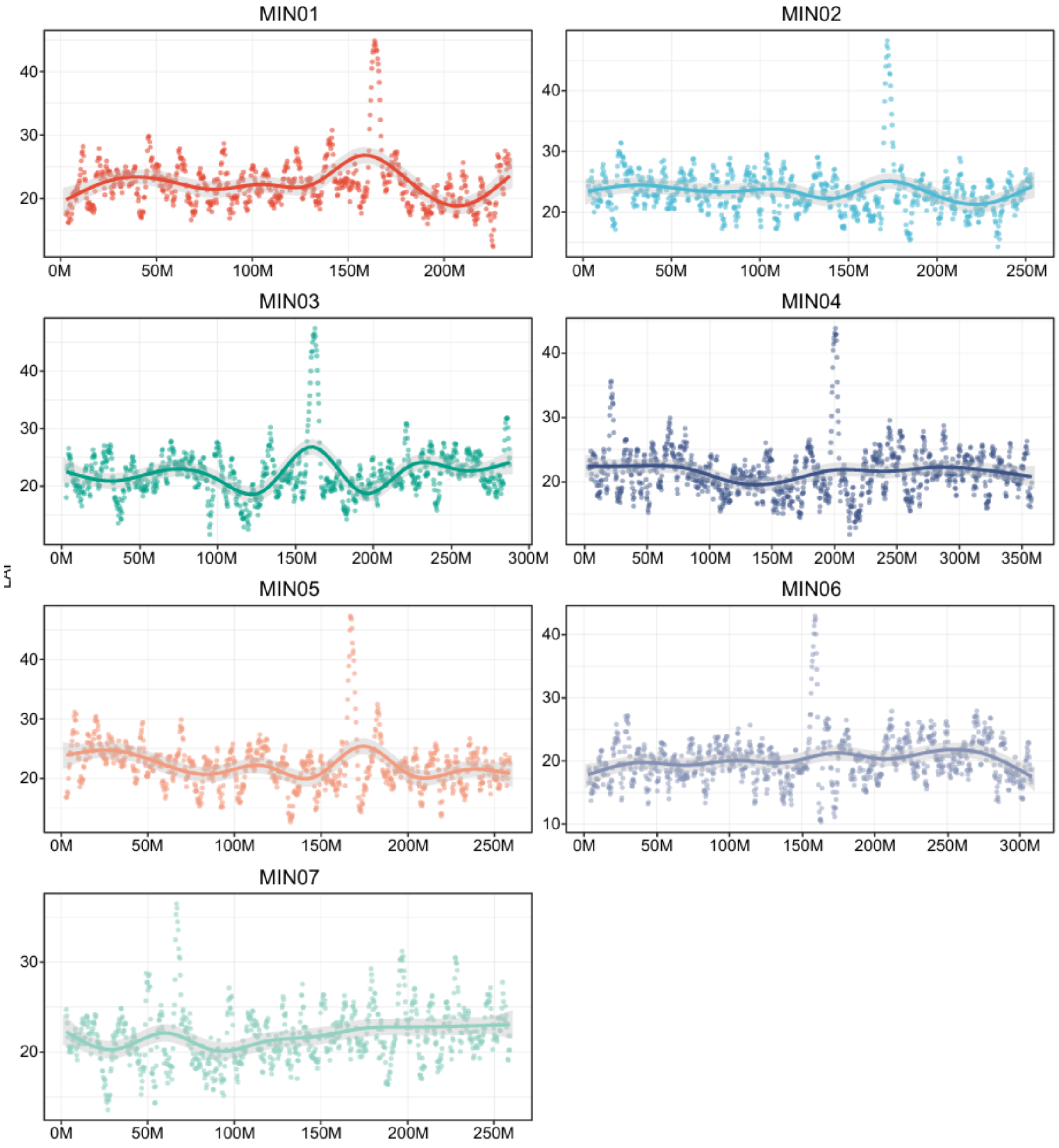

Supplementary Figure 4: Whole genome chromosome-level LAI (LTR Assembly Index) score distribution in *M. incana*, representing the quality of whole genome assembly.

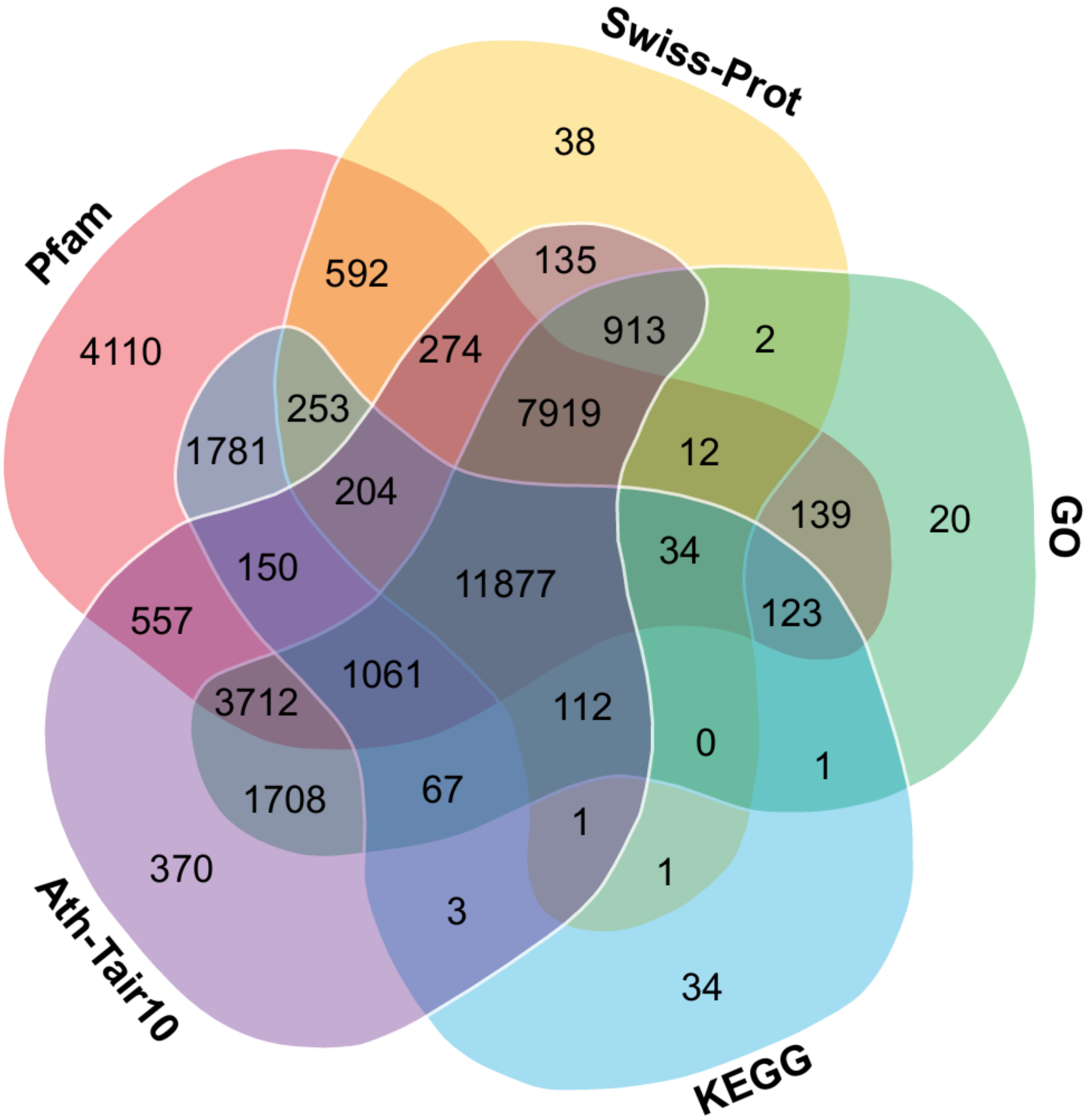

Supplementary Figure 5: The number of genes annotated in *M. incana* using different databases (Swiss-prot, GO, KEGG, Pfam and Ath_TAIR10).

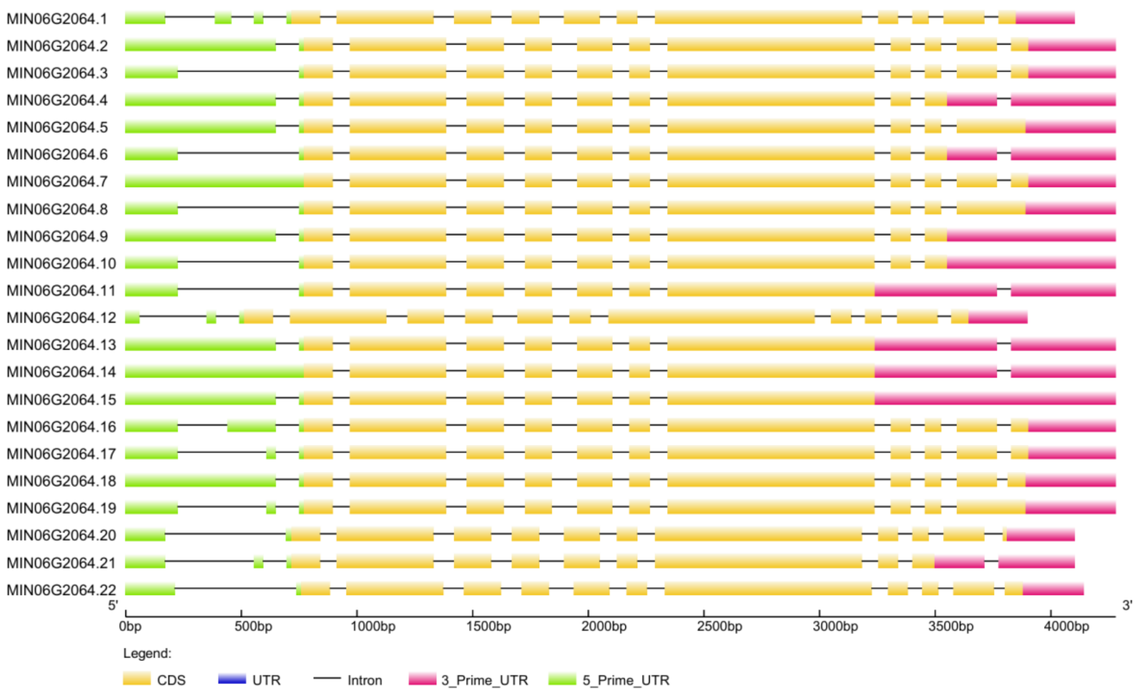

Supplementary Figure 6: The structure of gene MIN06G2064 (an ortholog of AT1G29400) in *M. incana* with the most (22) alternative splicing transcripts.

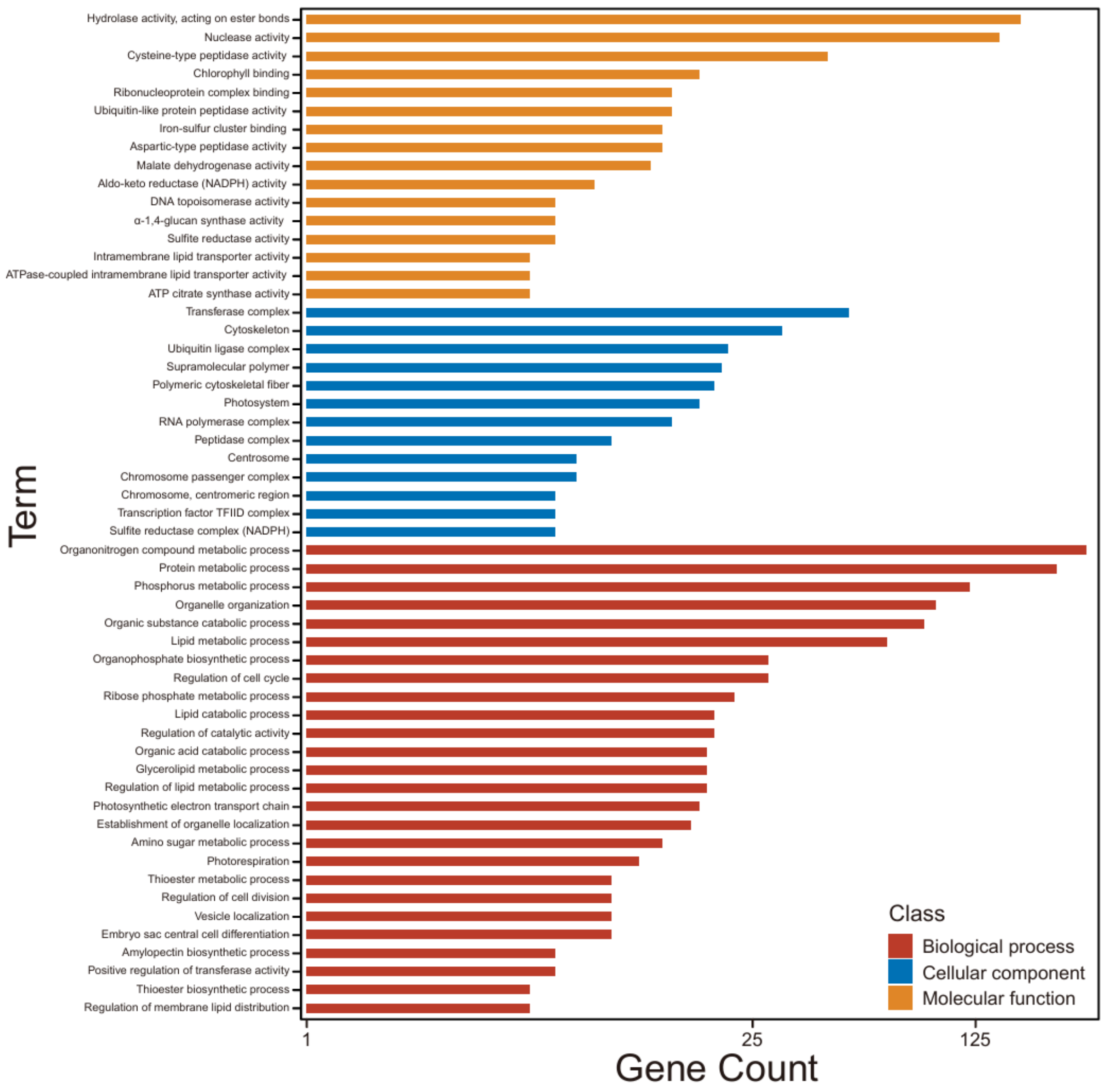

Supplementary Figure 7: The GO enrichments results of unique genes in *M. incana*.

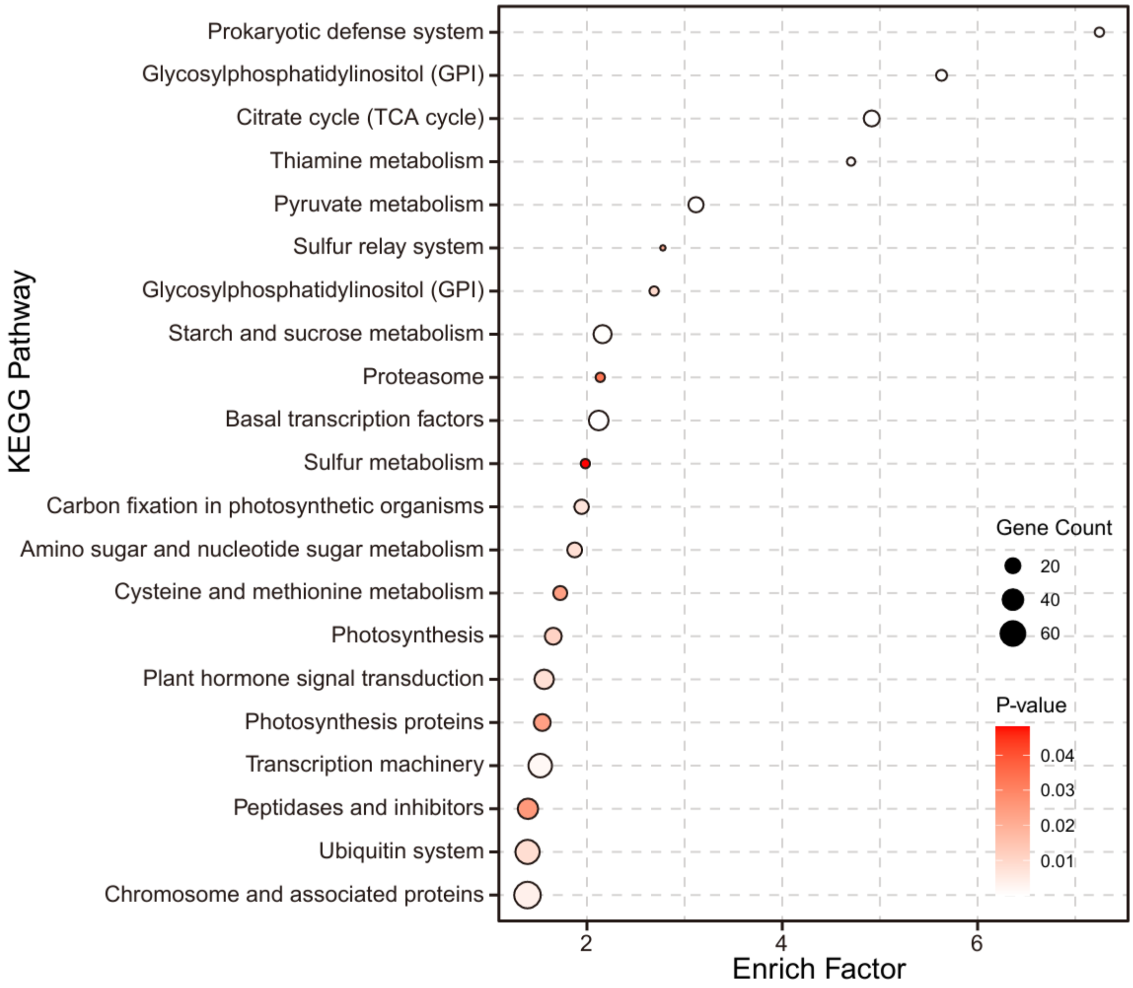

Supplementary Figure 8: The KEGG enrichment results of unique genes in *M. incana*

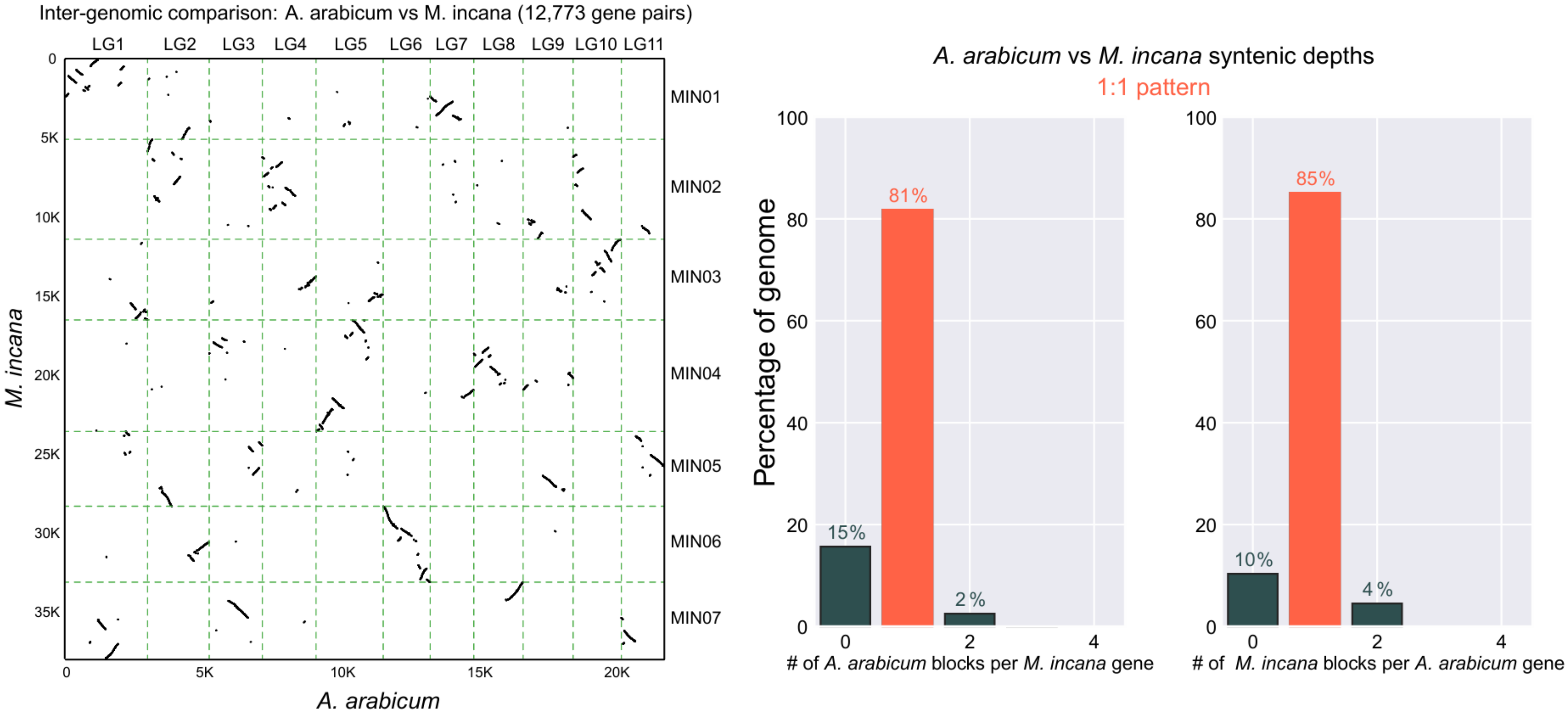

Supplementary Figure 9: The gene level synteny relationship between *Ae. arabicum* and *M. incana* based on 12,773 gene pairs and 1:1 syntenic pattern.

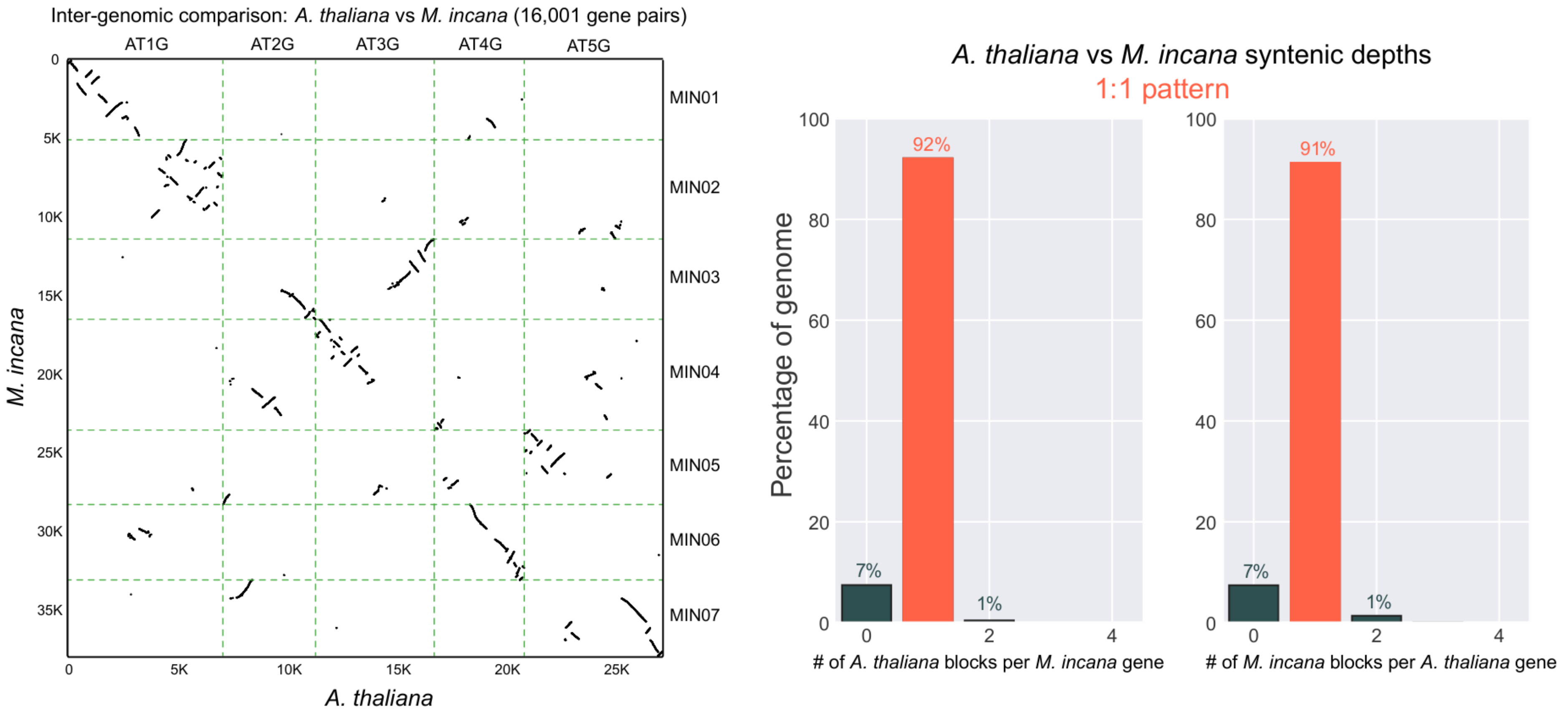

Supplementary Figure 10: The gene level synteny relationship between *A. thaliana* and *M. incana* based on 16,001 gene pairs and 1:1 syntenic pattern.

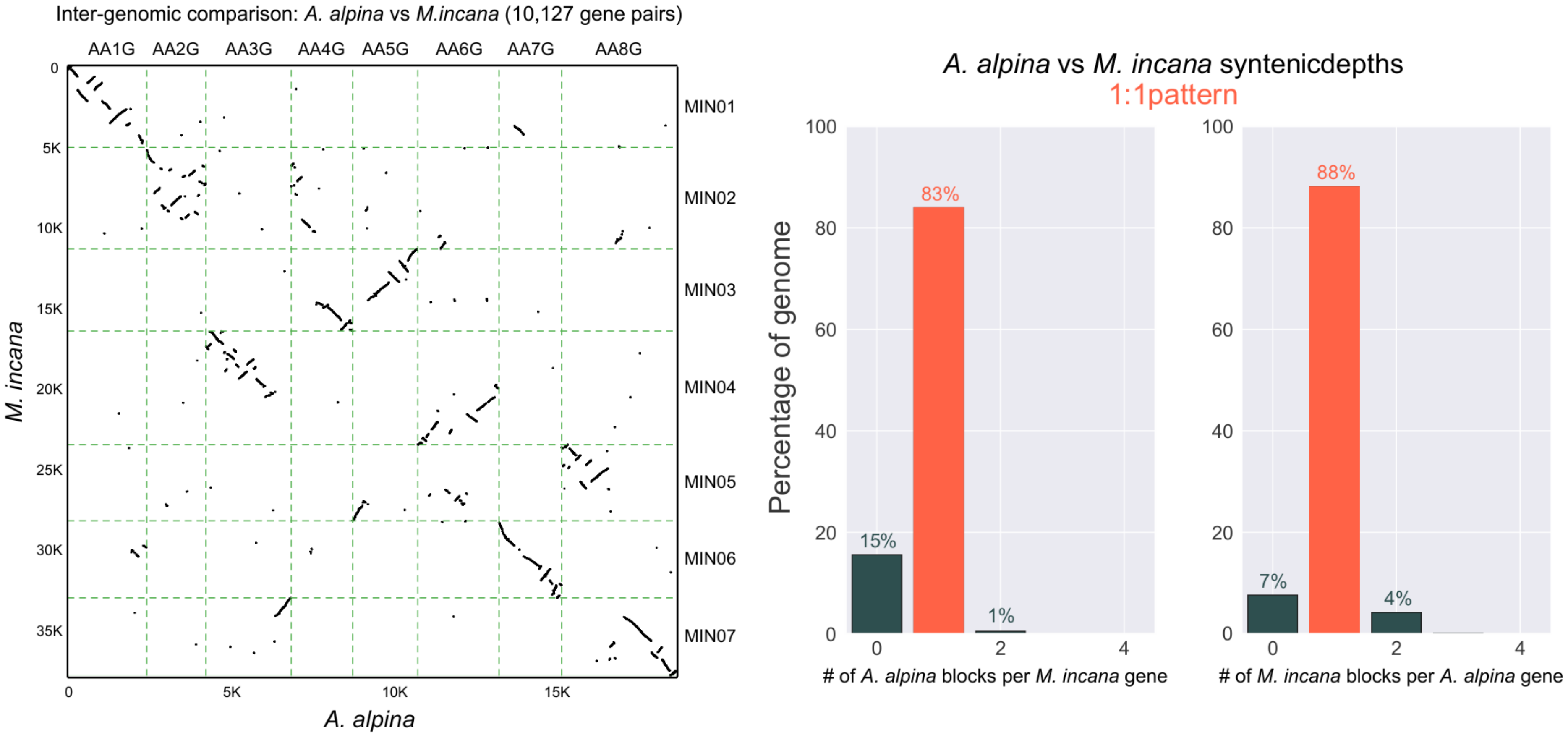

Supplementary Figure 11: The gene level synteny relationship between *Ar. alpina* and *M. incana* based on 10,127 gene pairs and 1:1 syntenic pattern.

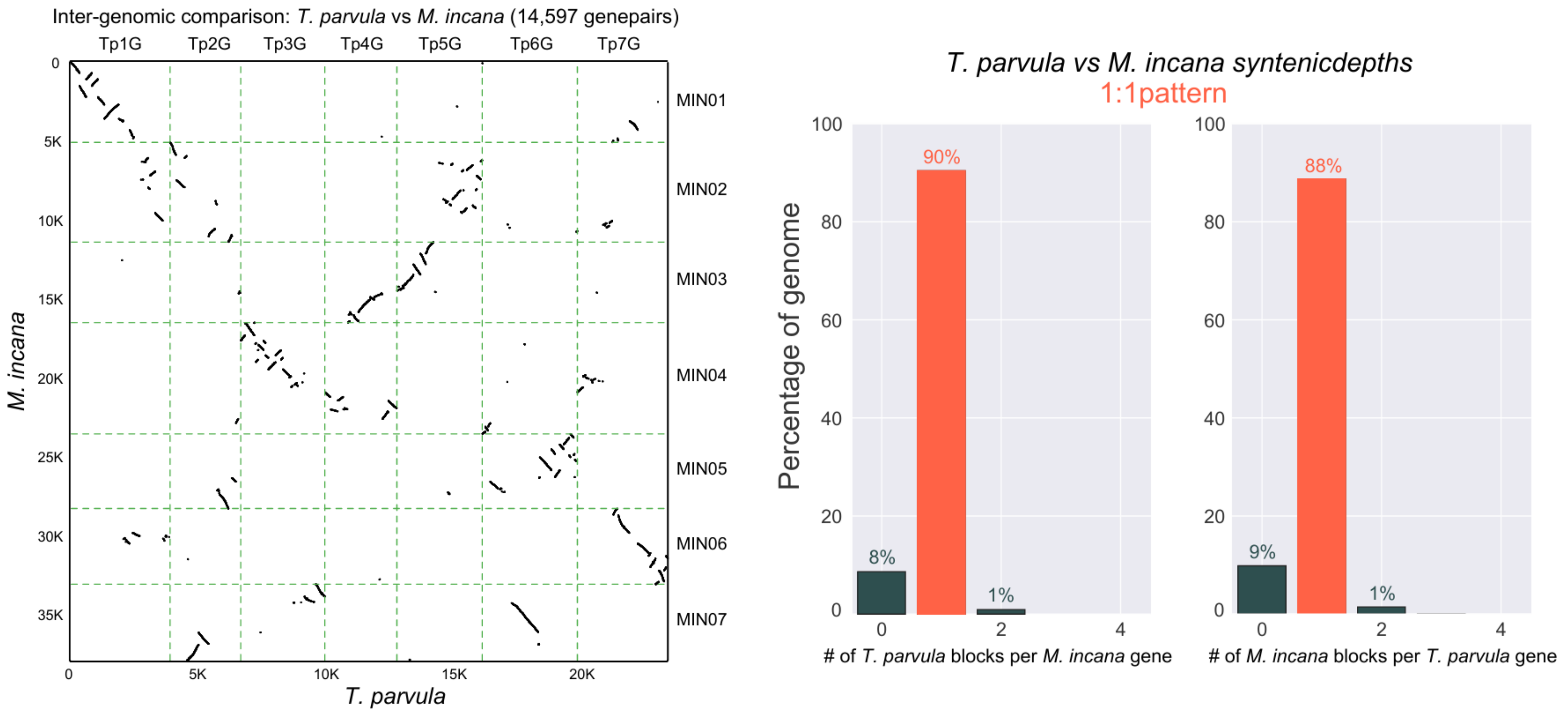

Supplementary Figure 12: The gene level synteny relationship between *T. parvula* and *M. incana* based on 14,597 gene pairs and 1:1 syntenic pattern.

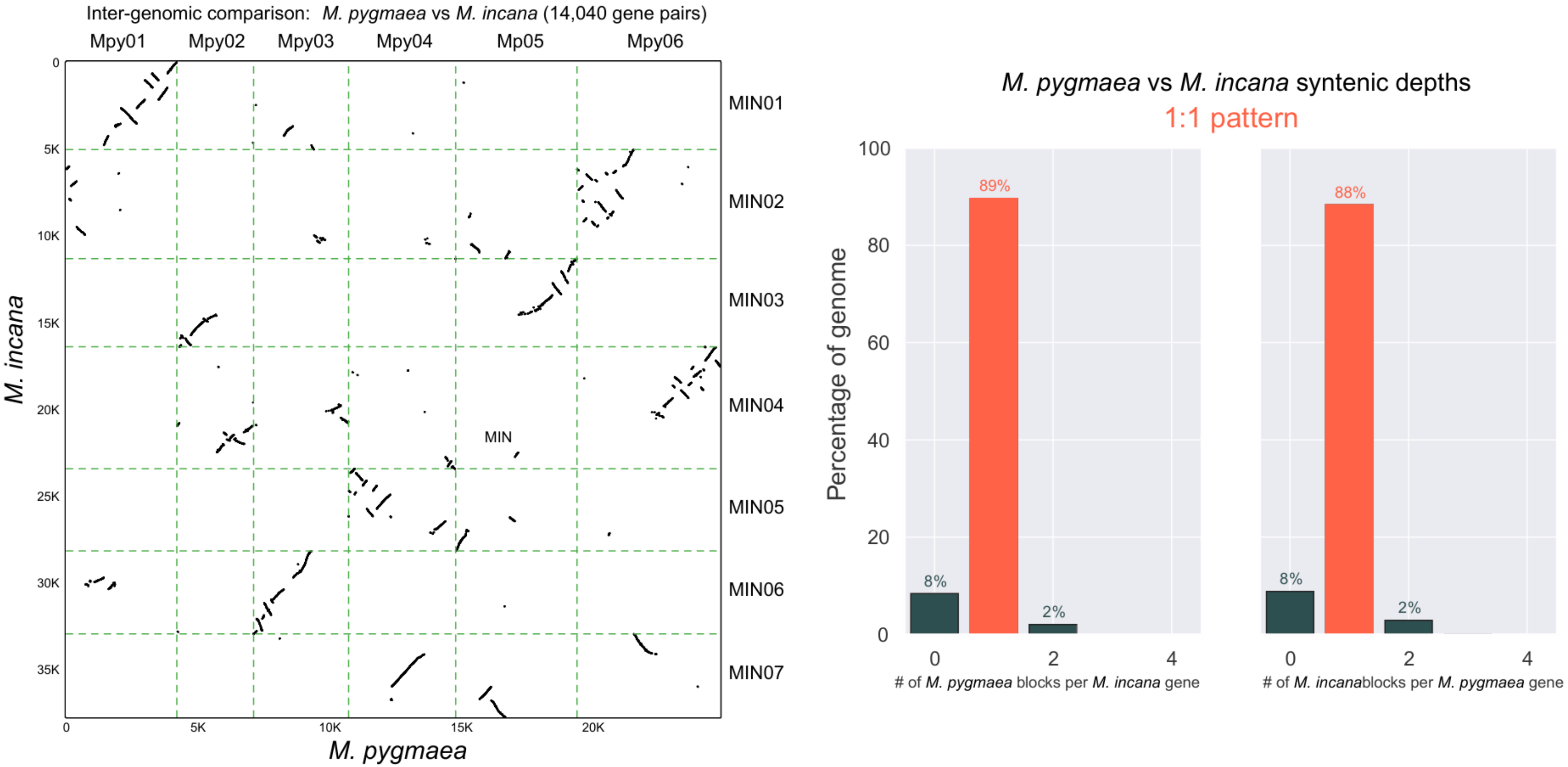

Supplementary Figure 13: The gene level synteny relationship between *Megadenia pygmaea* and *M. incana* based on 14,040 gene pairs and 1:1 syntenic pattern.

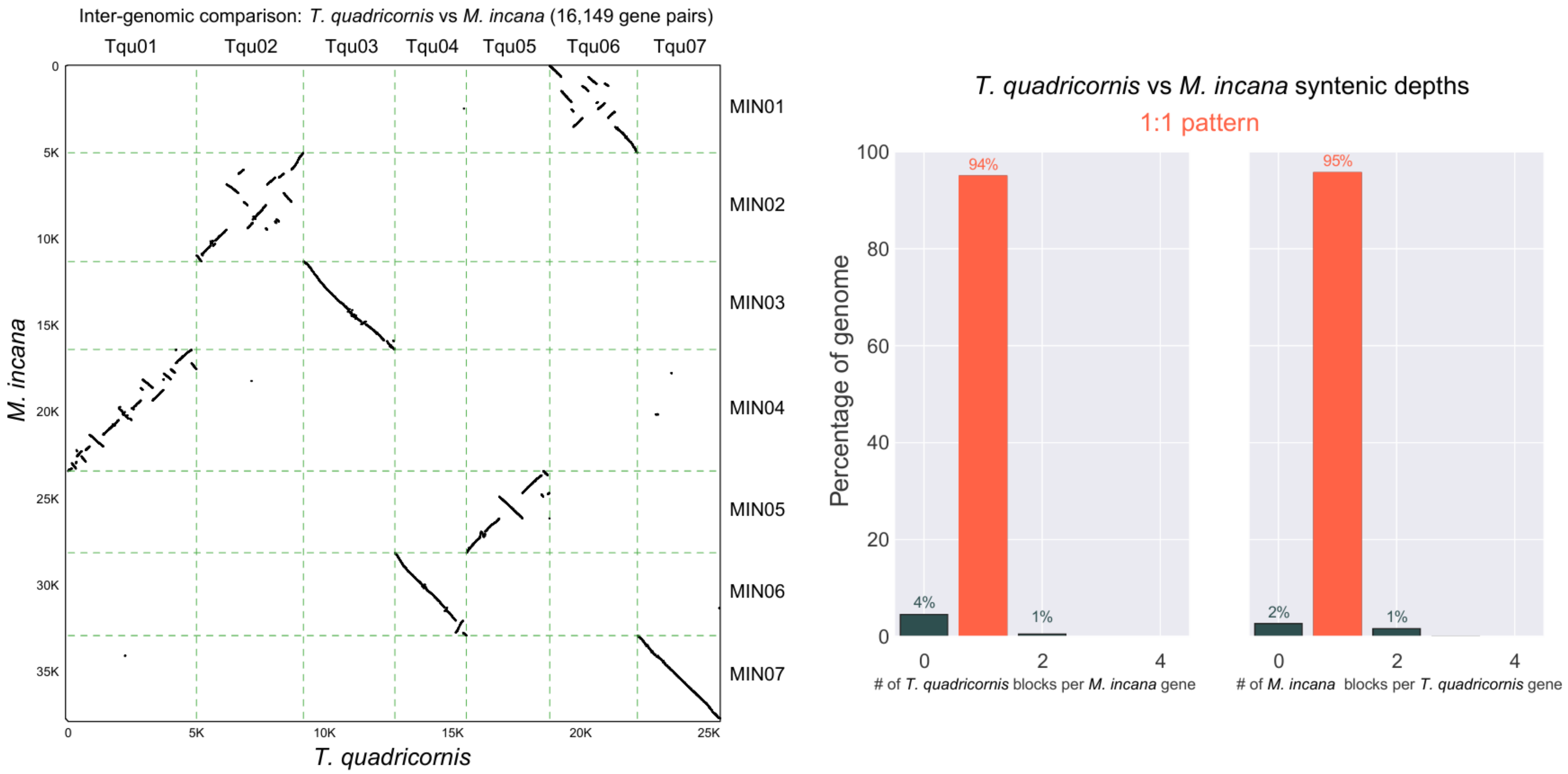

Supplementary Figure 14: The gene level synteny relationship between *T. quadricornis* and *M. incana* based on 16,149 gene pairs and 1:1 syntenic pattern.

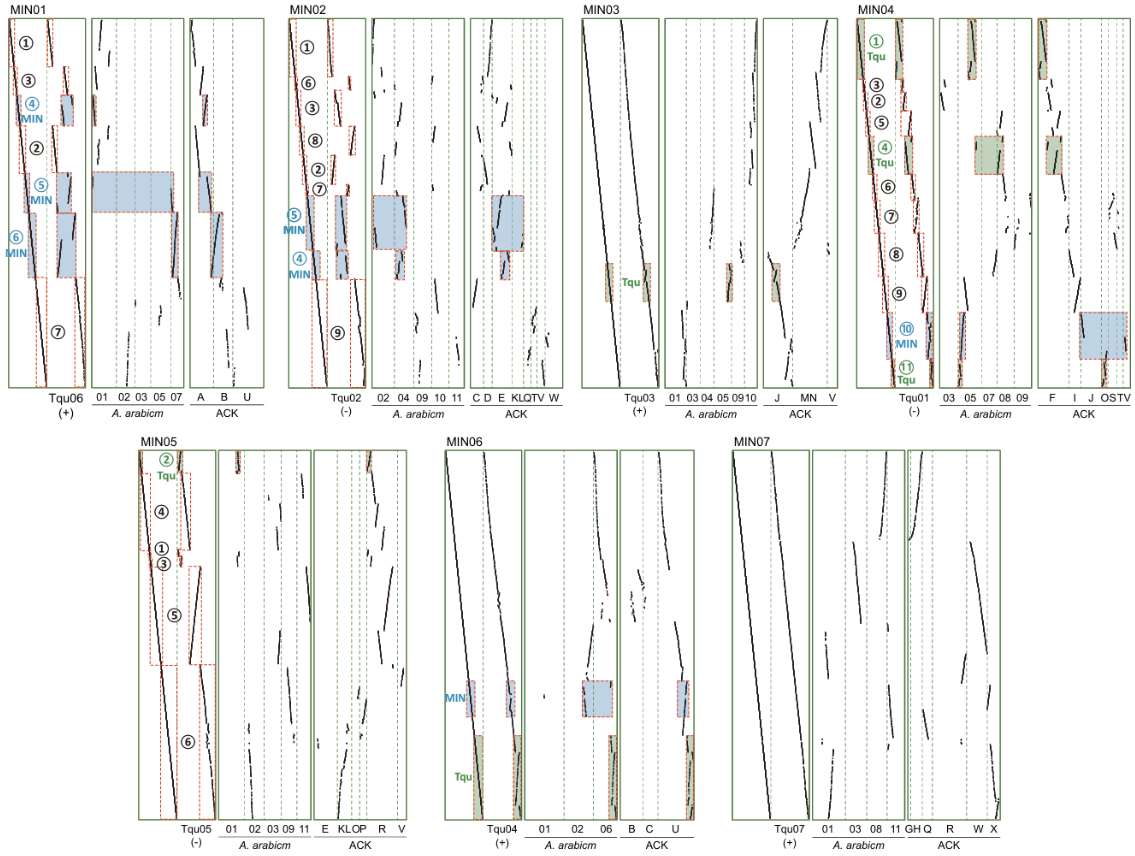

Supplementary Figure 15: The homologous dot-plots of all seven chromosome pairs (MIN01-Tqu06, MIN02-Tqu02, MIN03-Tqu03, MIN04-Tqu01, MIN05-Tqu05, MIN06-Tqu04 and MIN07-Tqu07) with major differences in genomic structure detected by collinearity analysis between *M. incana*, *T. quadricornis*, *Ae. arabicum* and ACK. The chromosome fragments (red dashed boxes) are truncated following the collinearity breaks in the *M. incana* and *T. quadricornis* genomes. The chromosome fragments were then reordered to infer an Ancestral Hesperodae Karyotype as the order in the chromosome of the AHK. The colored fragments (blue and green boxes) indicate that the fragment is more likely to get the original fragment structure.

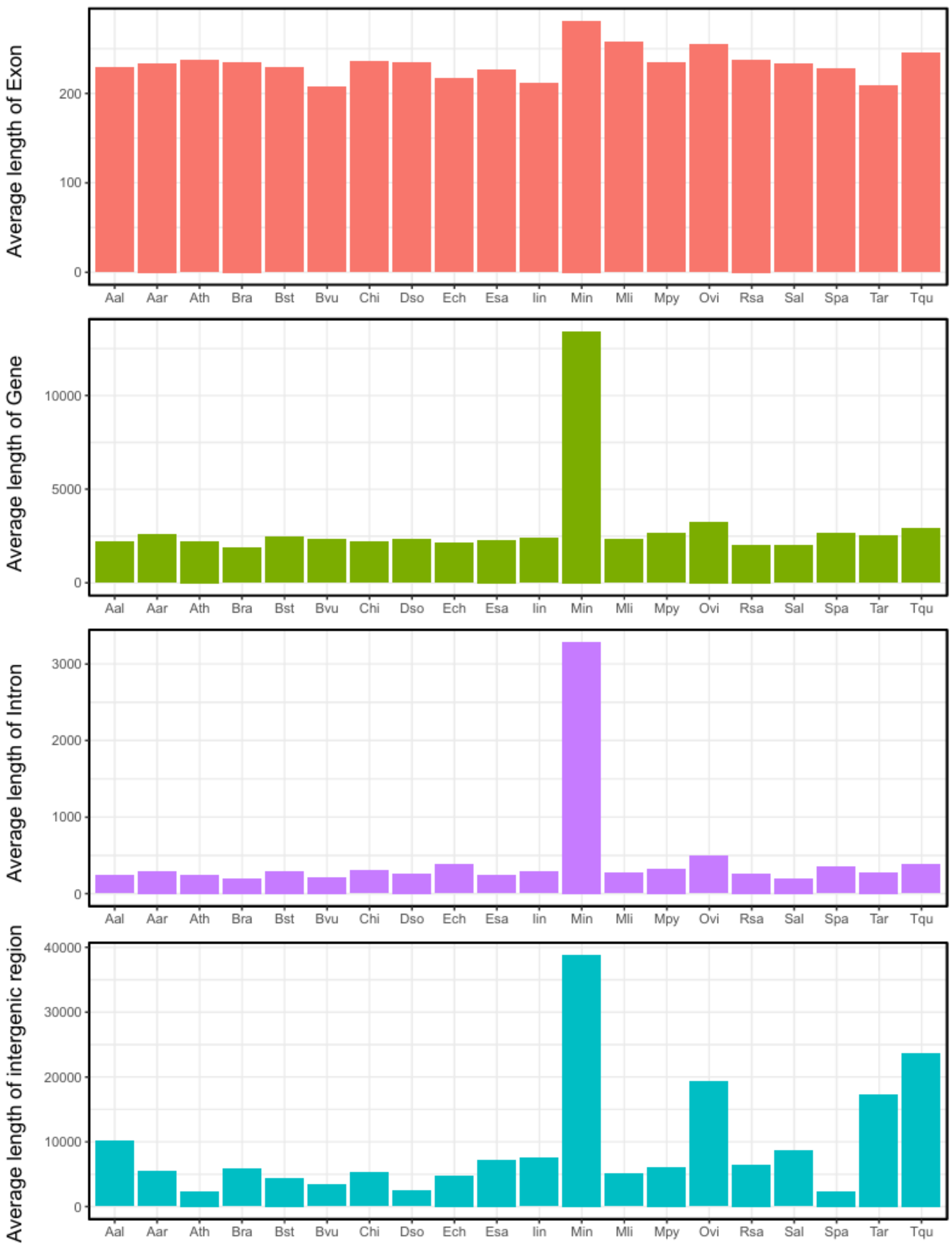

Supplementary Figure 16: Average length of exons, genes, introns and intergenic regions in *M. incana* and other 19 crucifer species. Nodes: Aal: *Arabis alpina*, Aar: *Aethionema arabicum*, Ath: *Arabidopsis thaliana*, Bra: *Brassica rapa*, Bst: *Boechera stricta*, Bvu: *Barbarea vulgaris*, Chi: *Cardamine hirsuta*, Dso: *Descurainia sophia*, Ech: *Erysimum cheiranthoides*, Esa: *Eutrema salsugineum*, Iin: *Isatis indigotica*, Min: *Matthiola incana*, Mli: *Meniocus linifolius*, Mpy: *Megadenia pygmaea*, Ovi: *Orychophragmus violaceus*, Rsa: *Raphanus sativus*, Sal: *Sinapis alba*, Tar: *Thlaspi arvense,* Spa: *Schrenkiella parvula*, Tqu: *Tetracme quadricornis*.

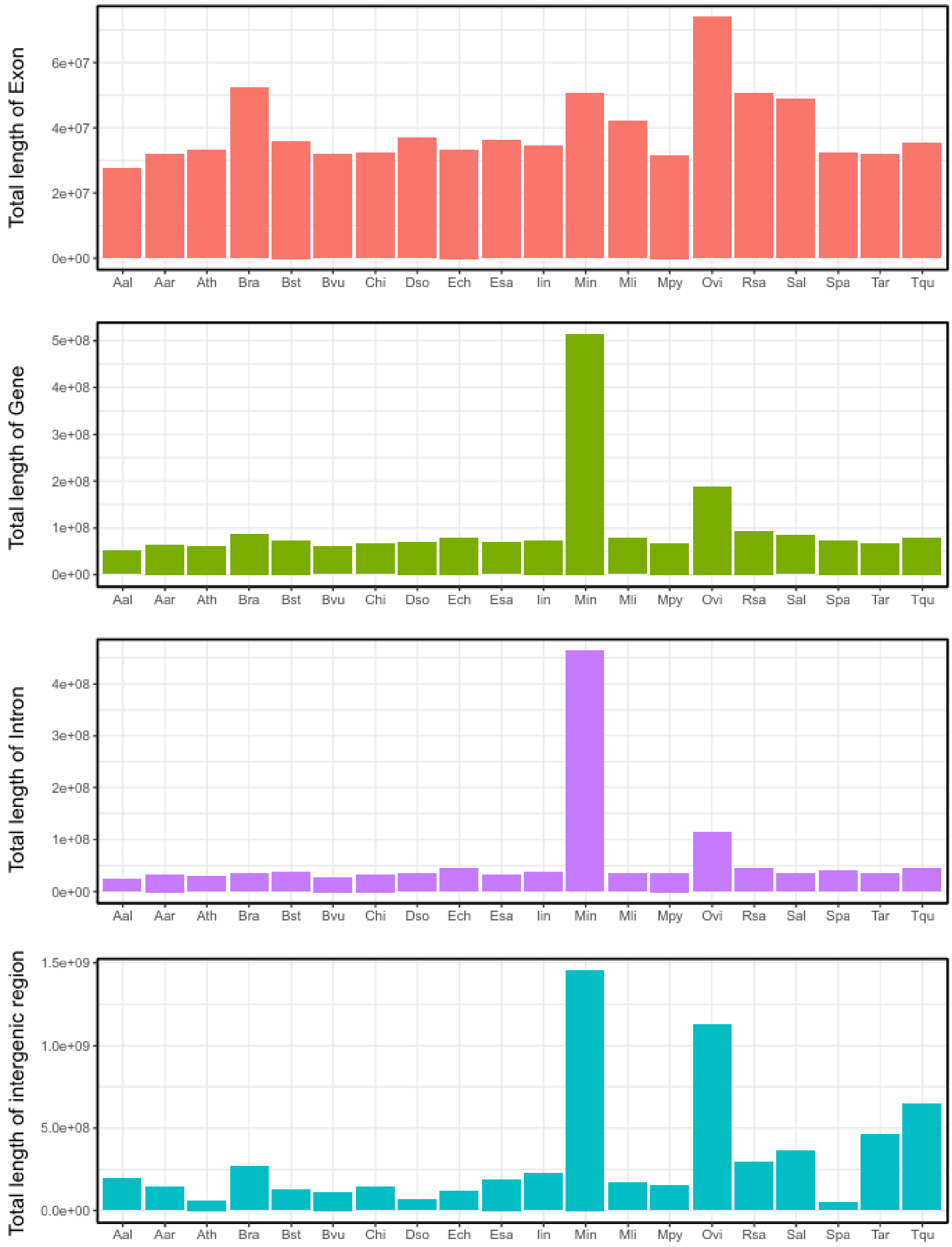

Supplementary Figure 17: Total length of exons, genes, introns and intergenic regions in *M. incana* and other 19 crucifer species. Nodes: Aal: *Arabis alpina*, Aar: *Aethionema arabicum*, Ath: *Arabidopsis thaliana*, Bra: *Brassica rapa*, Bst: *Boechera stricta*, Bvu: *Barbarea vulgaris*, Chi: *Cardamine hirsuta*, Dso: *Descurainia sophia*, Ech: *Erysimum cheiranthoides*, Esa: *Eutrema salsugineum*, Iin: *Isatis indigotica*, Min: *Matthiola incana*, Mli: *Meniocus linifolius*, Mpy: *Megadenia pygmaea*, Ovi: *Orychophragmus violaceus*, Rsa: *Raphanus sativus*, Sal: *Sinapis alba*, Tar: *Thlaspi arvense,* Spa: *Schrenkiella parvula*, Tqu: *Tetracme quadricornis*.

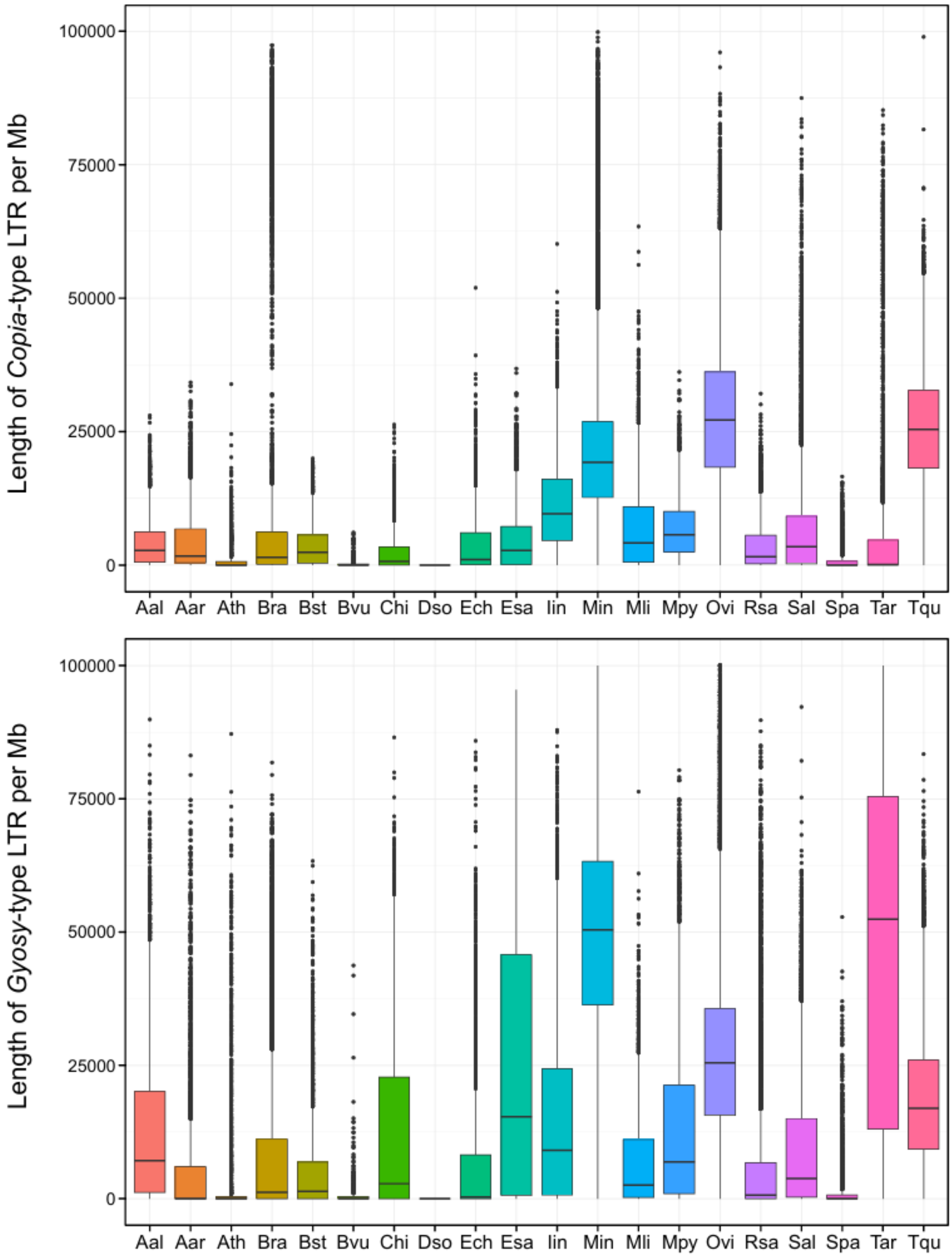

Supplementary Figure 18: The length of *Copia*- and *Gypsy*-type LTR-RTs per Mb in *M. incana* and other 19 species. Nodes: Aal: *Arabis alpina*, Aar: *Aethionema arabicum*, Ath: *Arabidopsis thaliana*, Bra: *Brassica rapa*, Bst: *Boechera stricta*, Bvu: *Barbarea vulgaris*, Chi: *Cardamine hirsuta*, Dso: *Descurainia sophia*, Ech: *Erysimum cheiranthoides*, Esa: *Eutrema salsugineum*, Iin: *Isatis indigotica*, Min: *Matthiola incana*, Mli: *Meniocus linifolius*, Mpy: *Megadenia pygmaea*, Ovi: *Orychophragmus violaceus*, Rsa: *Raphanus sativus*, Sal: *Sinapis alba*, Tar: *Thlaspi arvense,* Spa: *Schrenkiella parvula*, Tqu: *Tetracme quadricornis*.

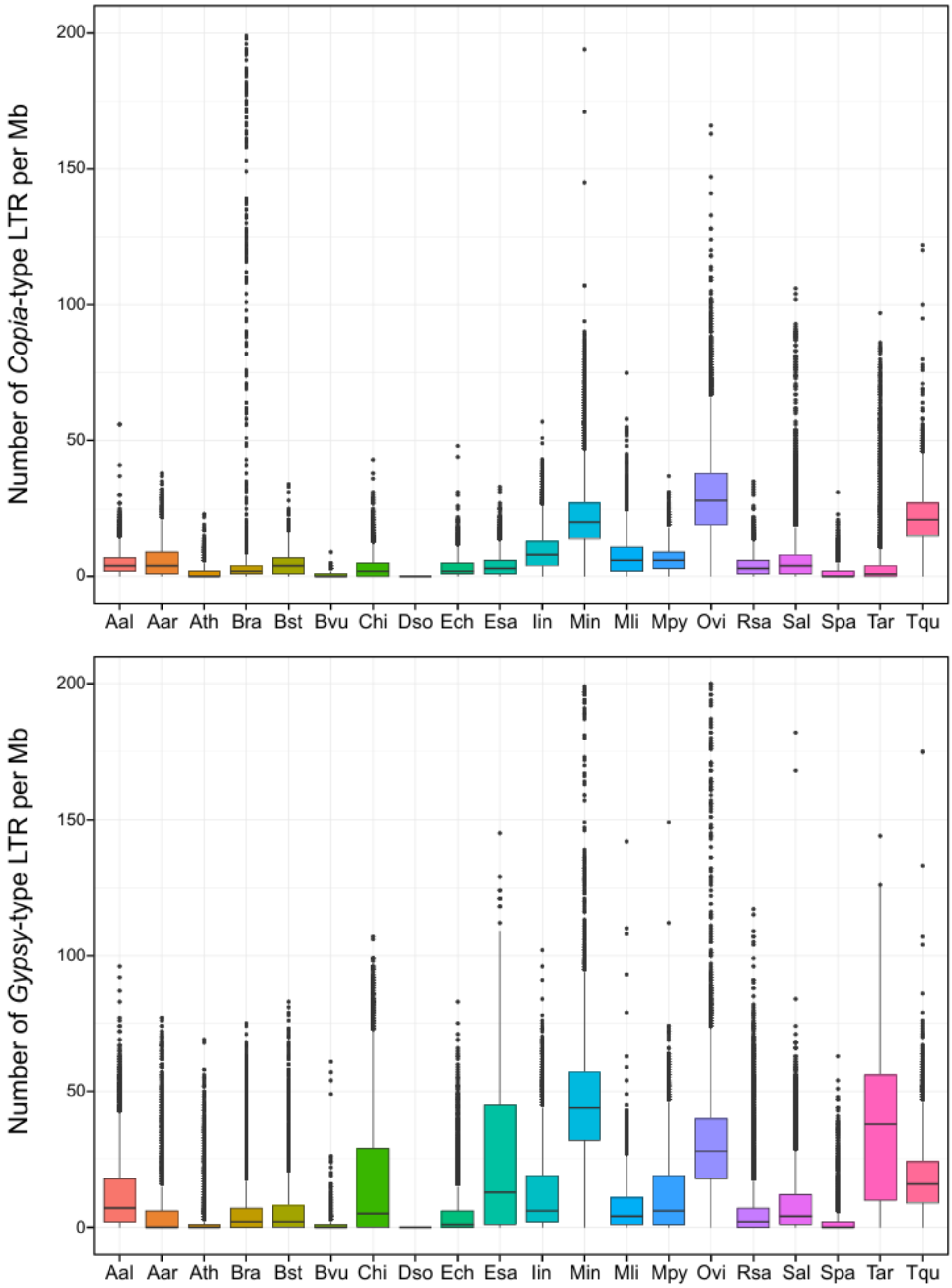

Supplementary Figure 19: The number of *Copia*- and *Gypsy*-type LTR-RTs per Mb in *M. incana* and other 19 species. Nodes: Aal: *Arabis alpina*, Aar: *Aethionema arabicum*, Ath: *Arabidopsis thaliana*, Bra: *Brassica rapa*, Bst: *Boechera stricta*, Bvu: *Barbarea vulgaris*, Chi: *Cardamine hirsuta*, Dso: *Descurainia sophia*, Ech: *Erysimum cheiranthoides*, Esa: *Eutrema salsugineum*, Iin: *Isatis indigotica*, Min: *Matthiola incana*, Mli: *Meniocus linifolius*, Mpy: *Megadenia pygmaea*, Ovi: *Orychophragmus violaceus*, Rsa: *Raphanus sativus*, Sal: *Sinapis alba*, Tar: *Thlaspi arvense,* Spa: *Schrenkiella parvula*, Tqu: *Tetracme quadricornis*.

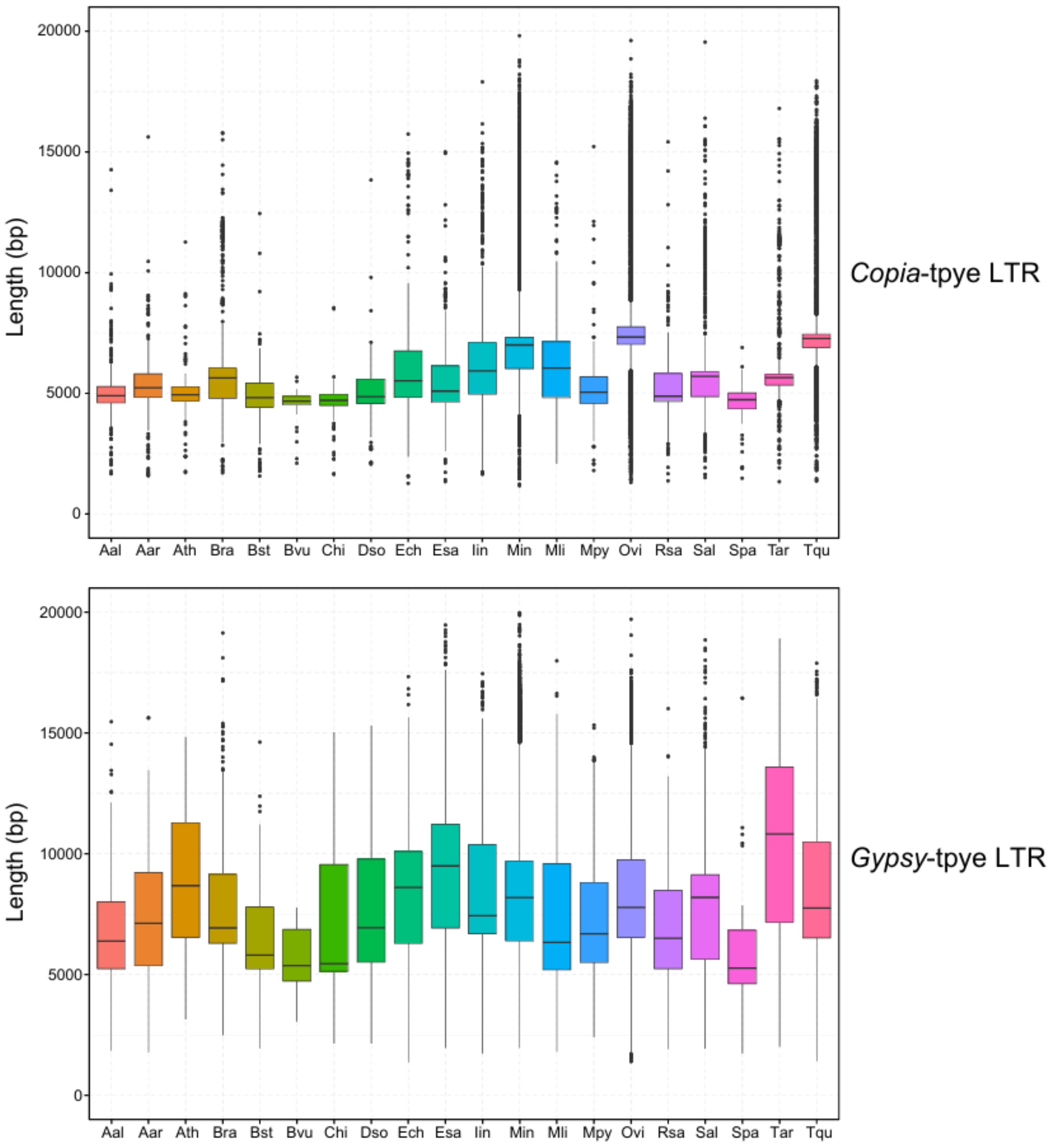

Supplementary Figure 20: The length of intact *Copia*- and *Gypsy*-type LTR-RTs in *M. incana* and other 19 species. Nodes: Aal: *Arabis alpina*, Aar: *Aethionema arabicum*, Ath: *Arabidopsis thaliana*, Bra: *Brassica rapa*, Bst: *Boechera stricta*, Bvu: *Barbarea vulgaris*, Chi: *Cardamine hirsuta*, Dso: *Descurainia sophia*, Ech: *Erysimum cheiranthoides*, Esa: *Eutrema salsugineum*, Iin: *Isatis indigotica*, Min: *Matthiola incana*, Mli: *Meniocus linifolius*, Mpy: *Megadenia pygmaea*, Ovi: *Orychophragmus violaceus*, Rsa: *Raphanus sativus*, Sal: *Sinapis alba*, Tar: *Thlaspi arvense,* Spa: *Schrenkiella parvula*, Tqu: *Tetracme quadricornis*.

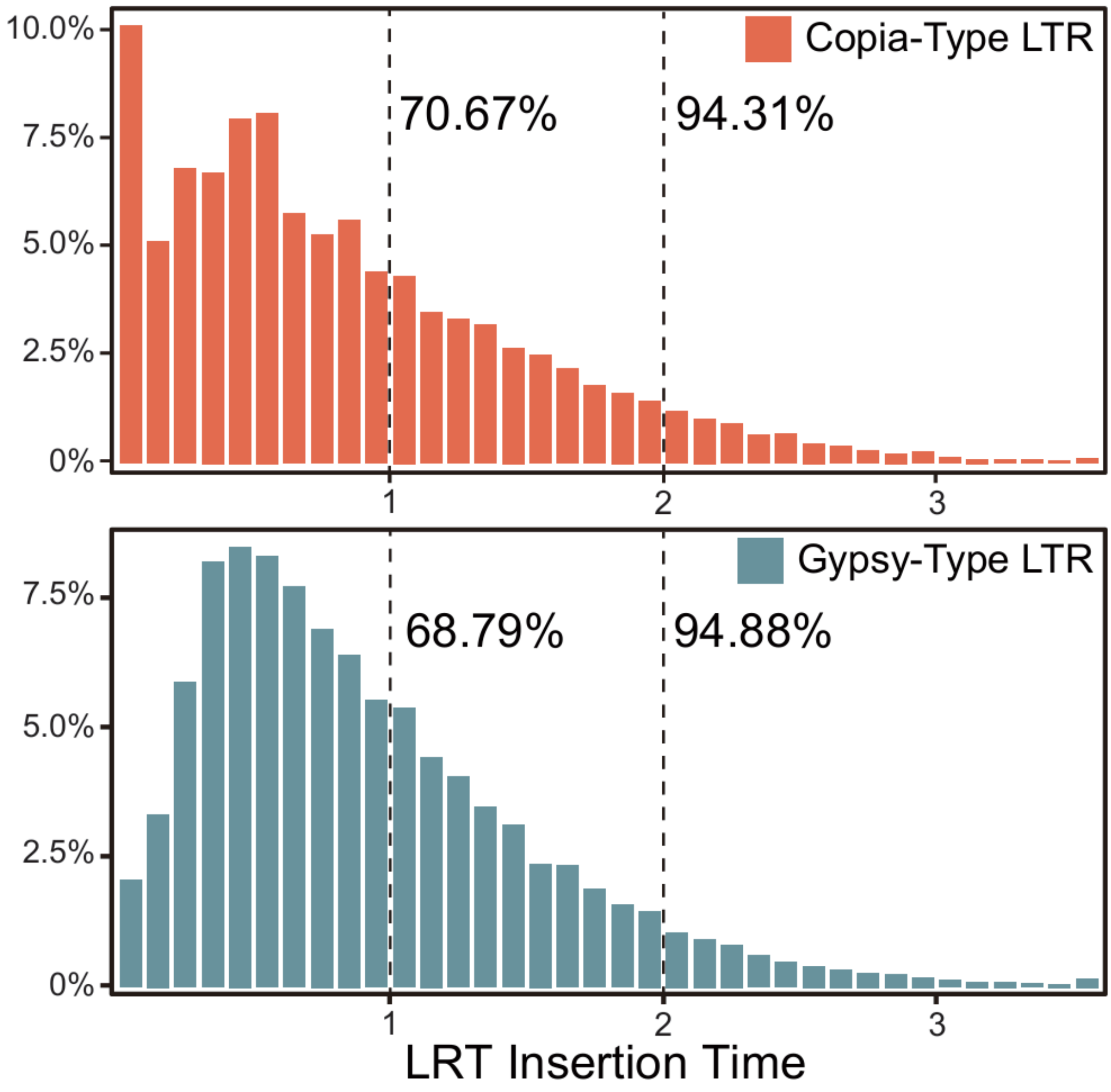

Supplementary Figure 21: The insertion time of *Copia*- and *Gypsy*-type LTR-RTs.

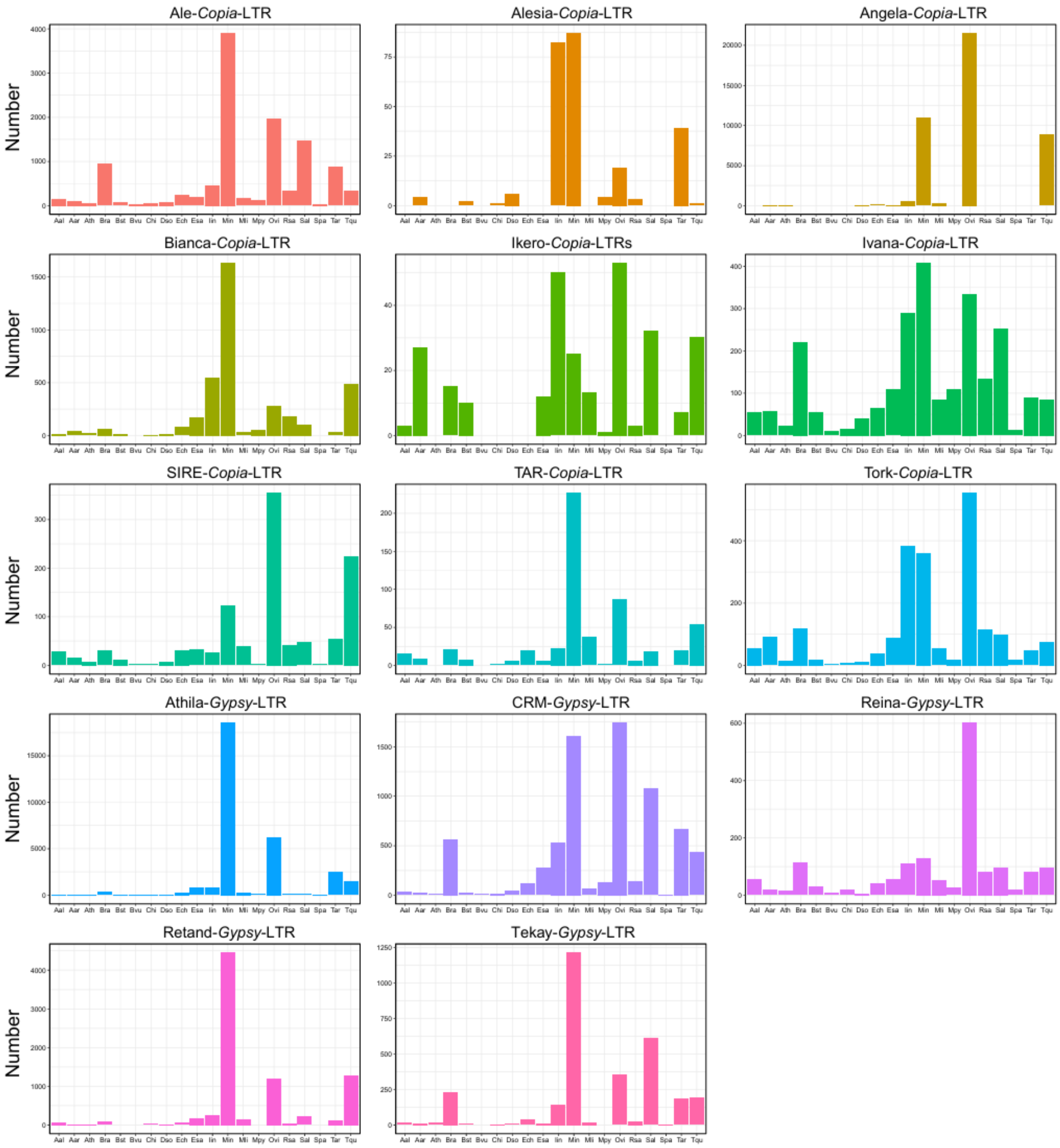

Supplementary Figure 22: The number of clades of *Copia*- and *Gypsy*-type LTR-RTs in *M. incana* and other 19 species. Nodes: Aal: *Arabis alpina*, Aar: *Aethionema arabicum*, Ath: *Arabidopsis thaliana*, Bra: *Brassica rapa*, Bst: *Boechera stricta*, Bvu: *Barbarea vulgaris*, Chi: *Cardamine hirsuta*, Dso: *Descurainia sophia*, Ech: *Erysimum cheiranthoides*, Esa: *Eutrema salsugineum*, Iin: *Isatis indigotica*, Min: *Matthiola incana*, Mli: *Meniocus linifolius*, Mpy: *Megadenia pygmaea*, Ovi: *Orychophragmus violaceus*, Rsa: *Raphanus sativus*, Sal: *Sinapis alba*, Tar: *Thlaspi arvense,* Spa: *Schrenkiella parvula*, Tqu: *Tetracme quadricornis*.

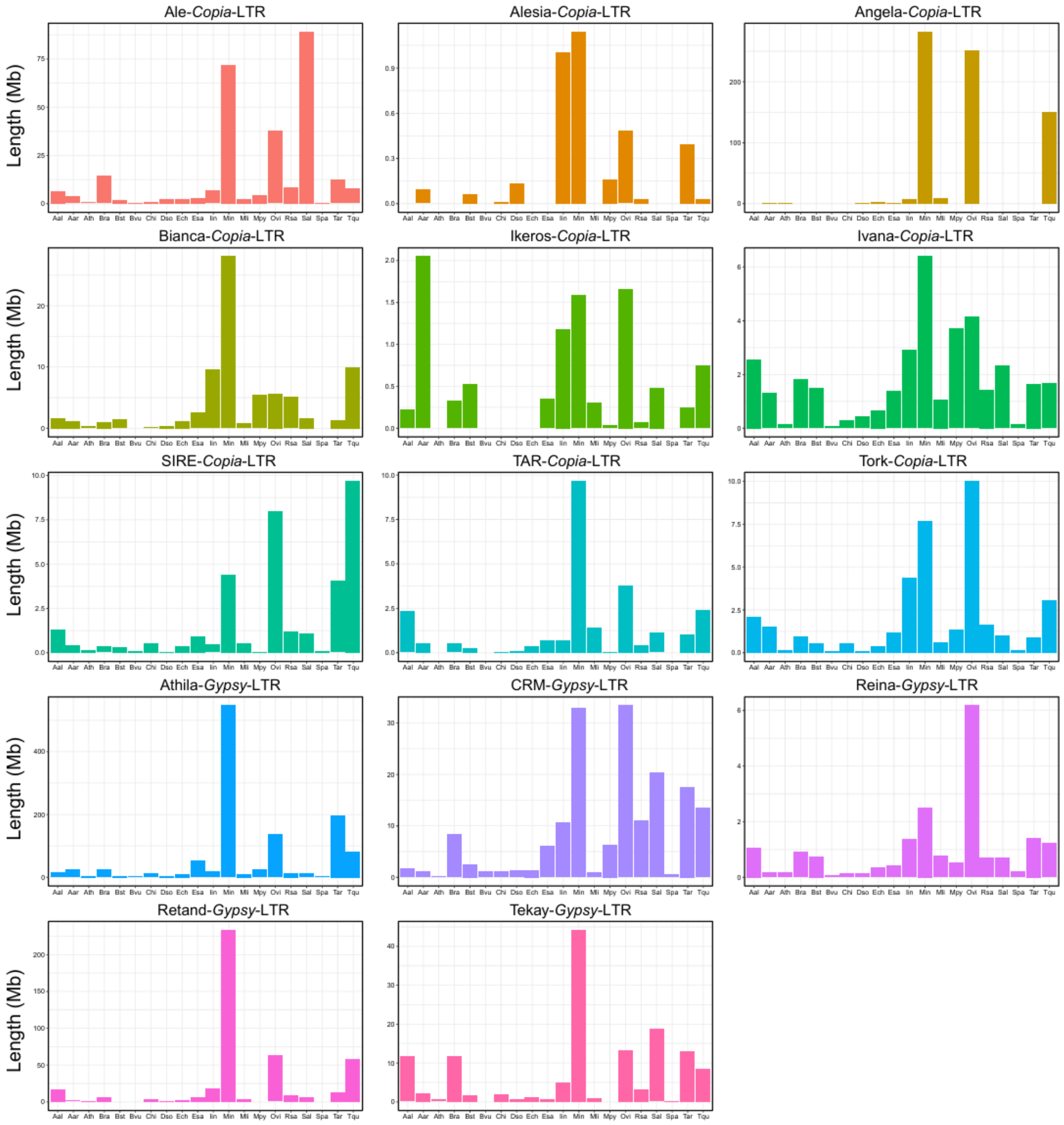

Supplementary Figure 23: The length of clades of *Copia*- and *Gypsy*-type LTR-RTs in *M. incana* and other 19 species. Nodes: Aal: *Arabis alpina*, Aar: *Aethionema arabicum*, Ath: *Arabidopsis thaliana*, Bra: *Brassica rapa*, Bst: *Boechera stricta*, Bvu: *Barbarea vulgaris*, Chi: *Cardamine hirsuta*, Dso: *Descurainia sophia*, Ech: *Erysimum cheiranthoides*, Esa: *Eutrema salsugineum*, Iin: *Isatis indigotica*, Min: *Matthiola incana*, Mli: *Meniocus linifolius*, Mpy: *Megadenia pygmaea*, Ovi: *Orychophragmus violaceus*, Rsa: *Raphanus sativus*, Sal: *Sinapis alba*, Tar: *Thlaspi arvense,* Spa: *Schrenkiella parvula*, Tqu: *Tetracme quadricornis*.

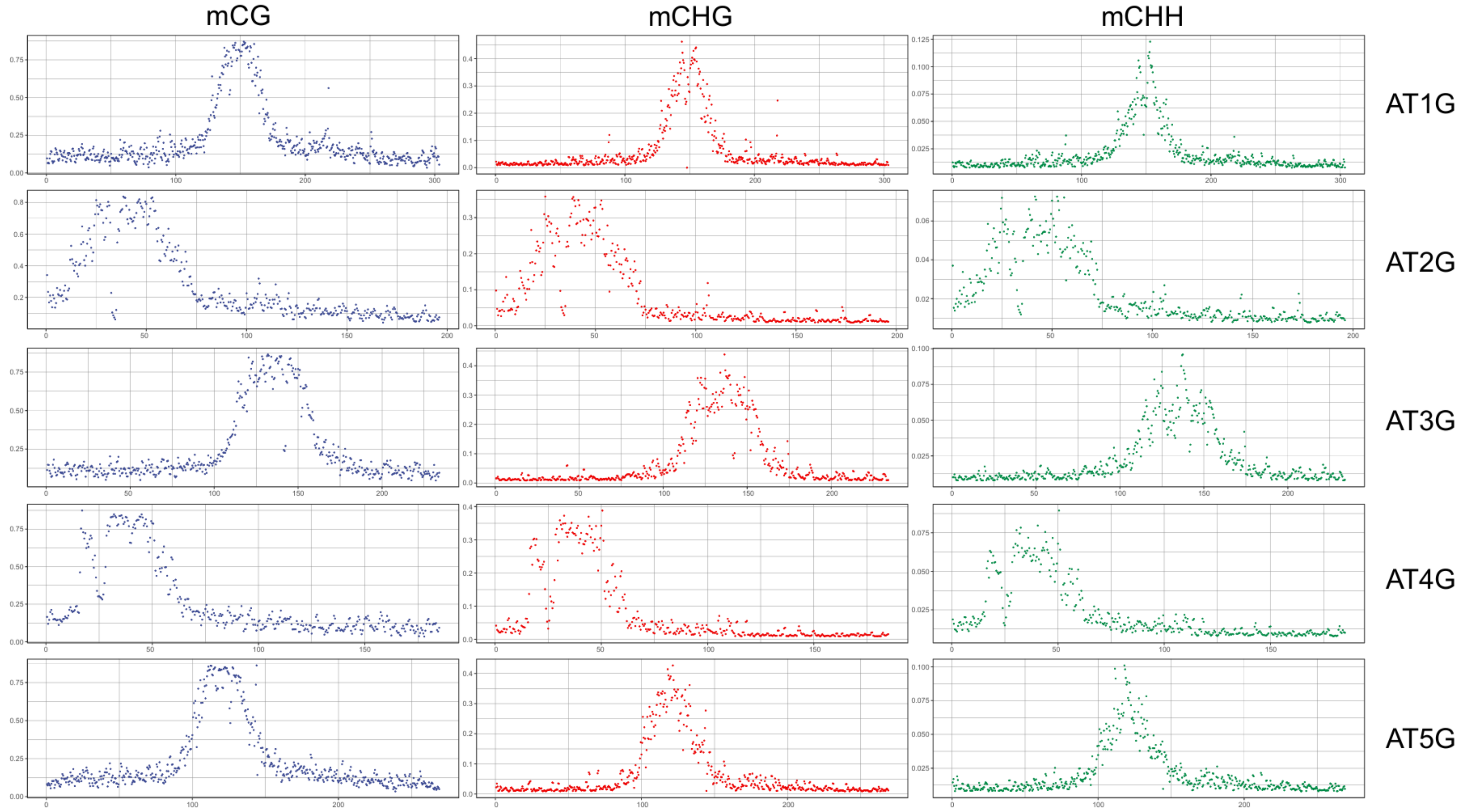

Supplementary Figure 24: Whole genome DNA methylation (CG, CHG and CHH) in *A. thaliana*.

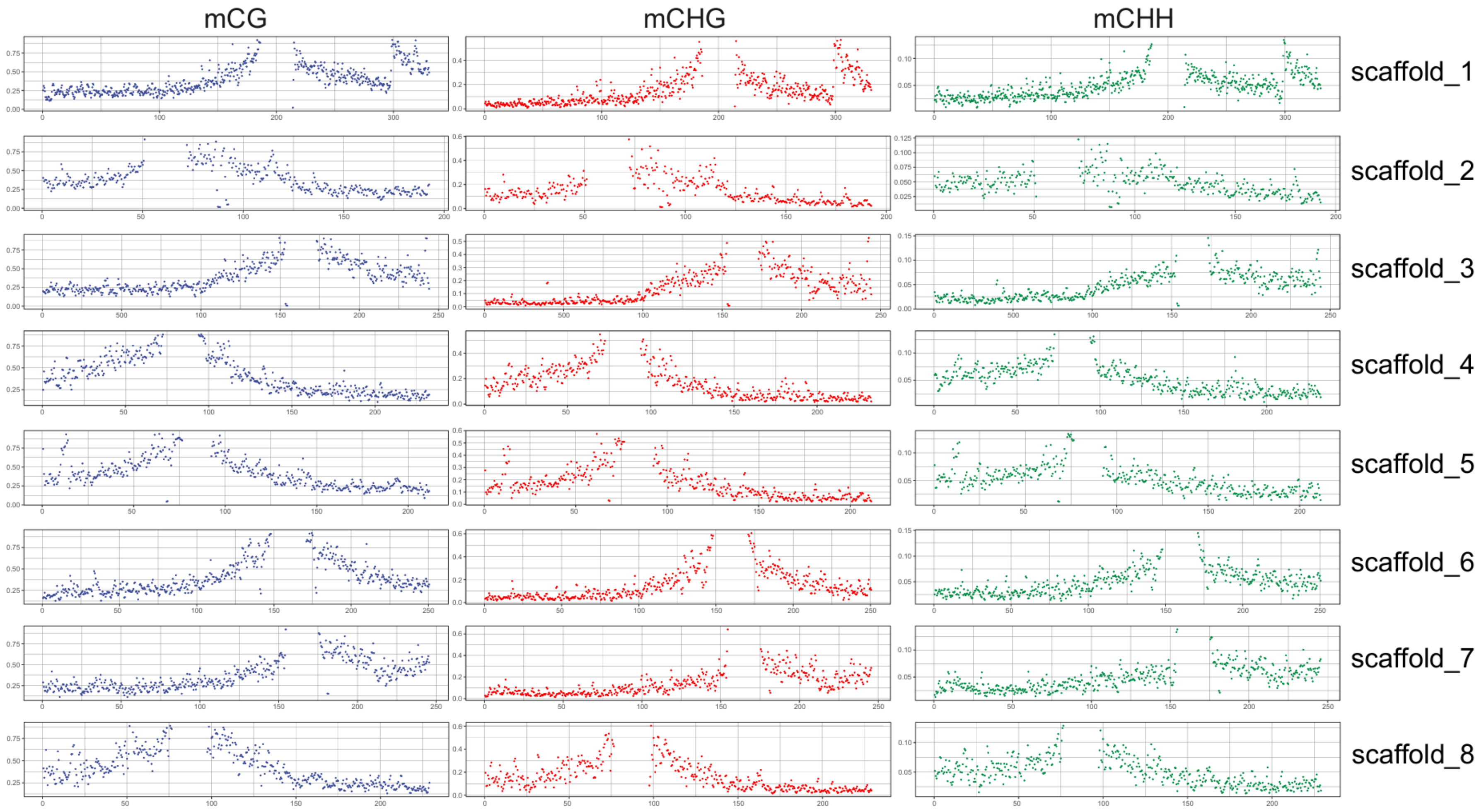

Supplementary Figure 25: Whole genome DNA methylation (CG, CHG and CHH) in *A. lyrata*.

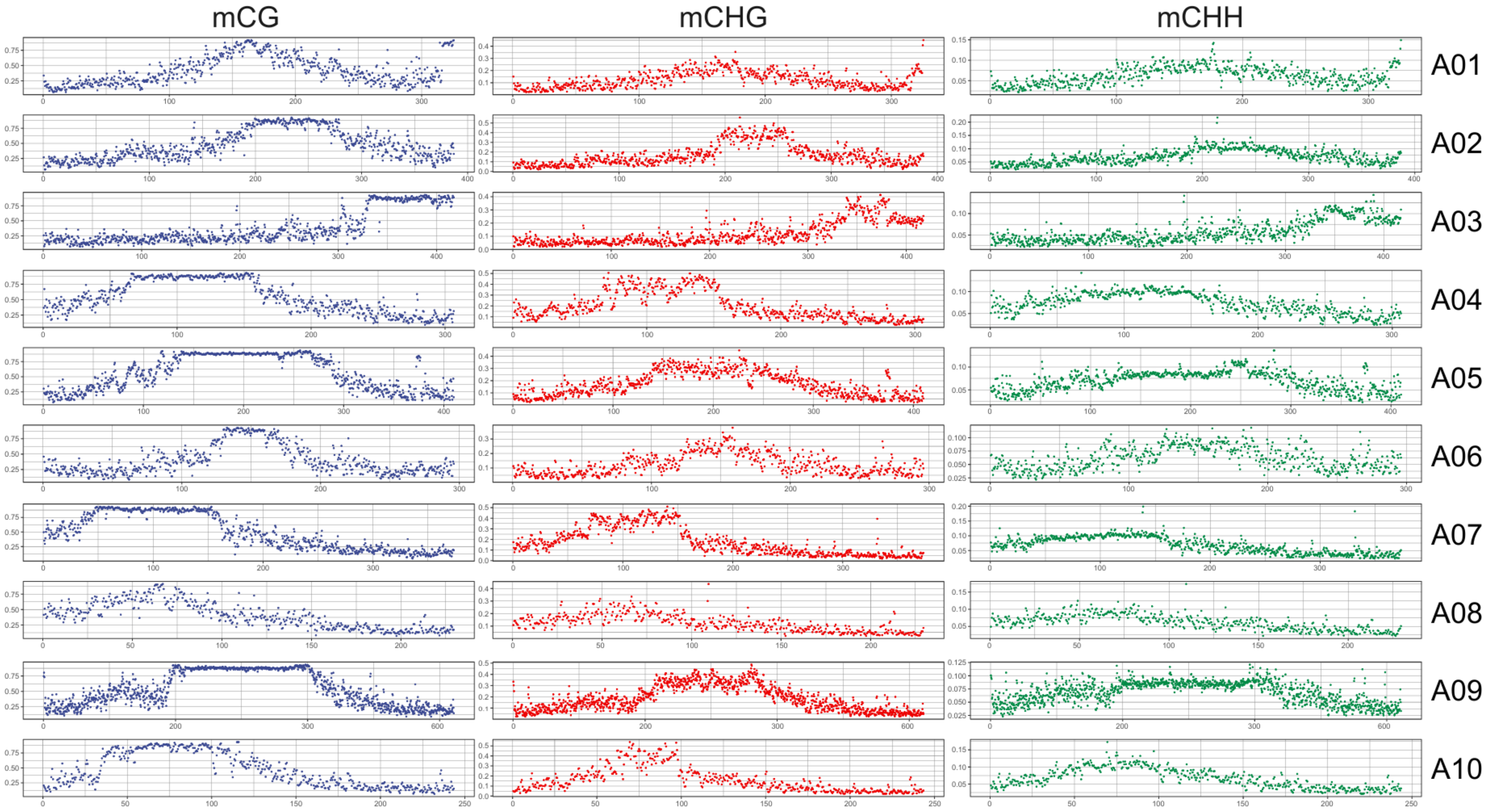

Supplementary Figure 26: Whole genome DNA methylation (CG, CHG and CHH) in *B. rapa*.

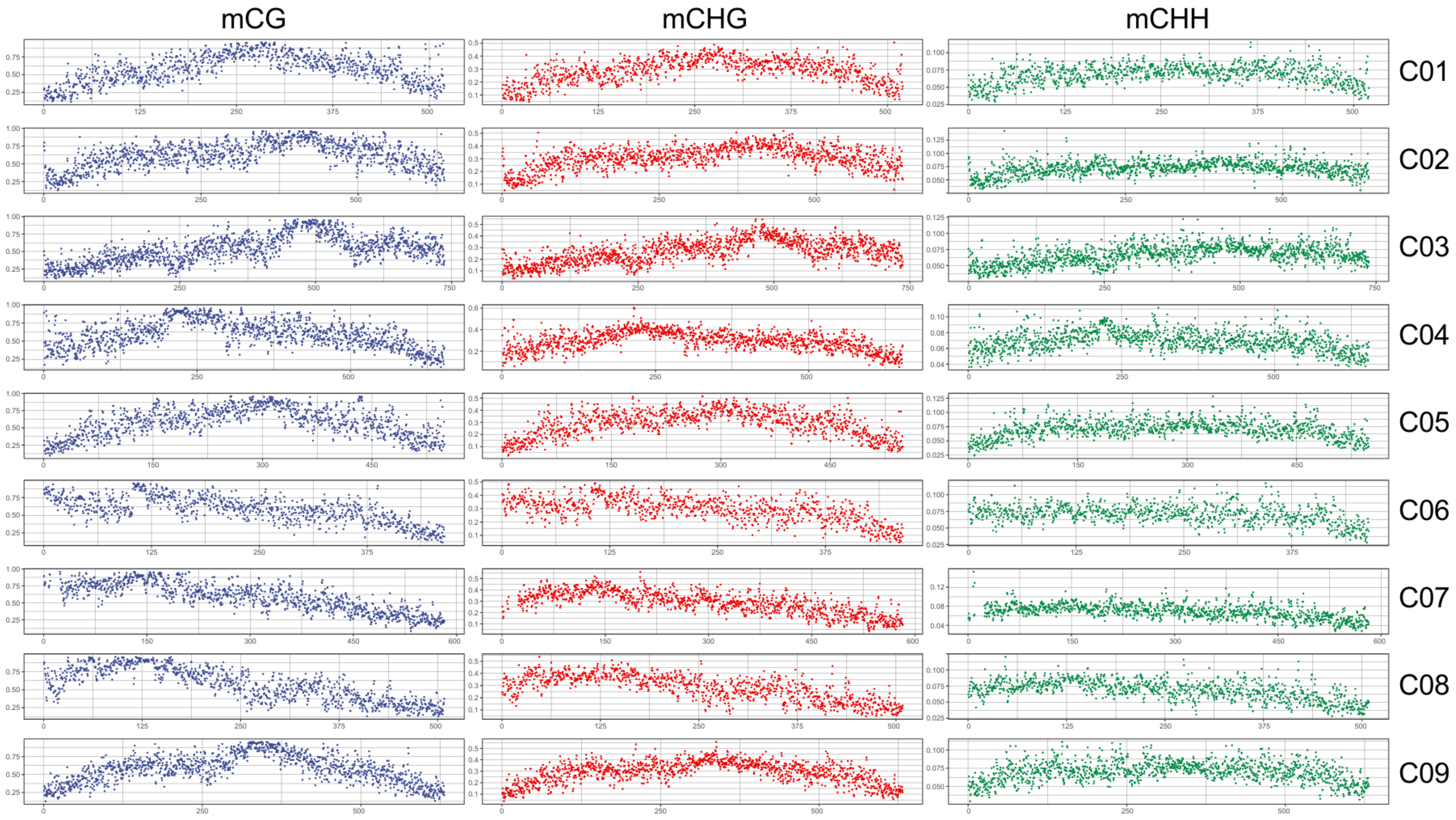

Supplementary Figure 27: Whole genome DNA methylation (CG, CHG and CHH) in *B. oleracea*.

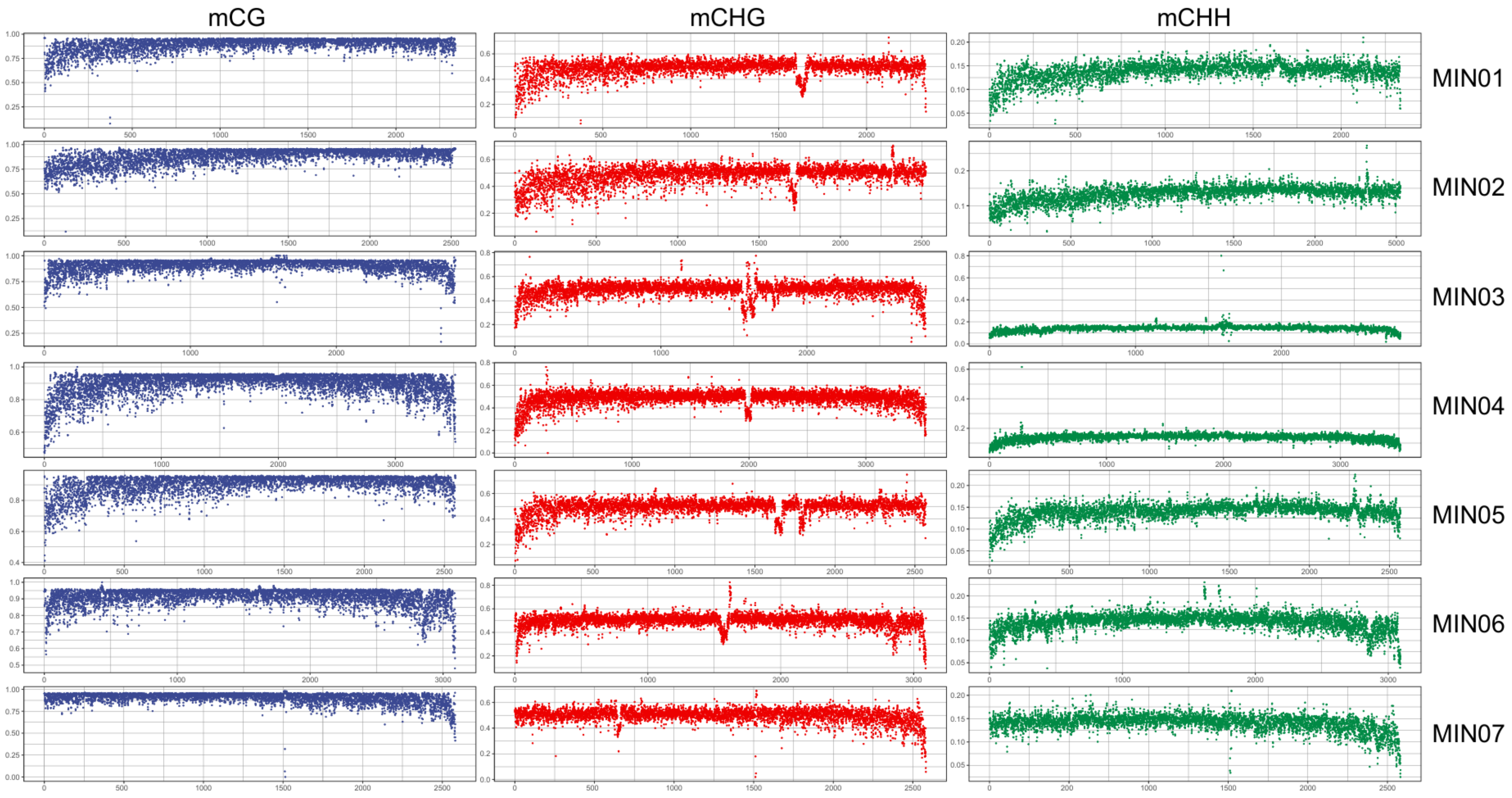

Supplementary Figure 28: Whole genome DNA methylation (CG, CHG and CHH) in *M. incana*.

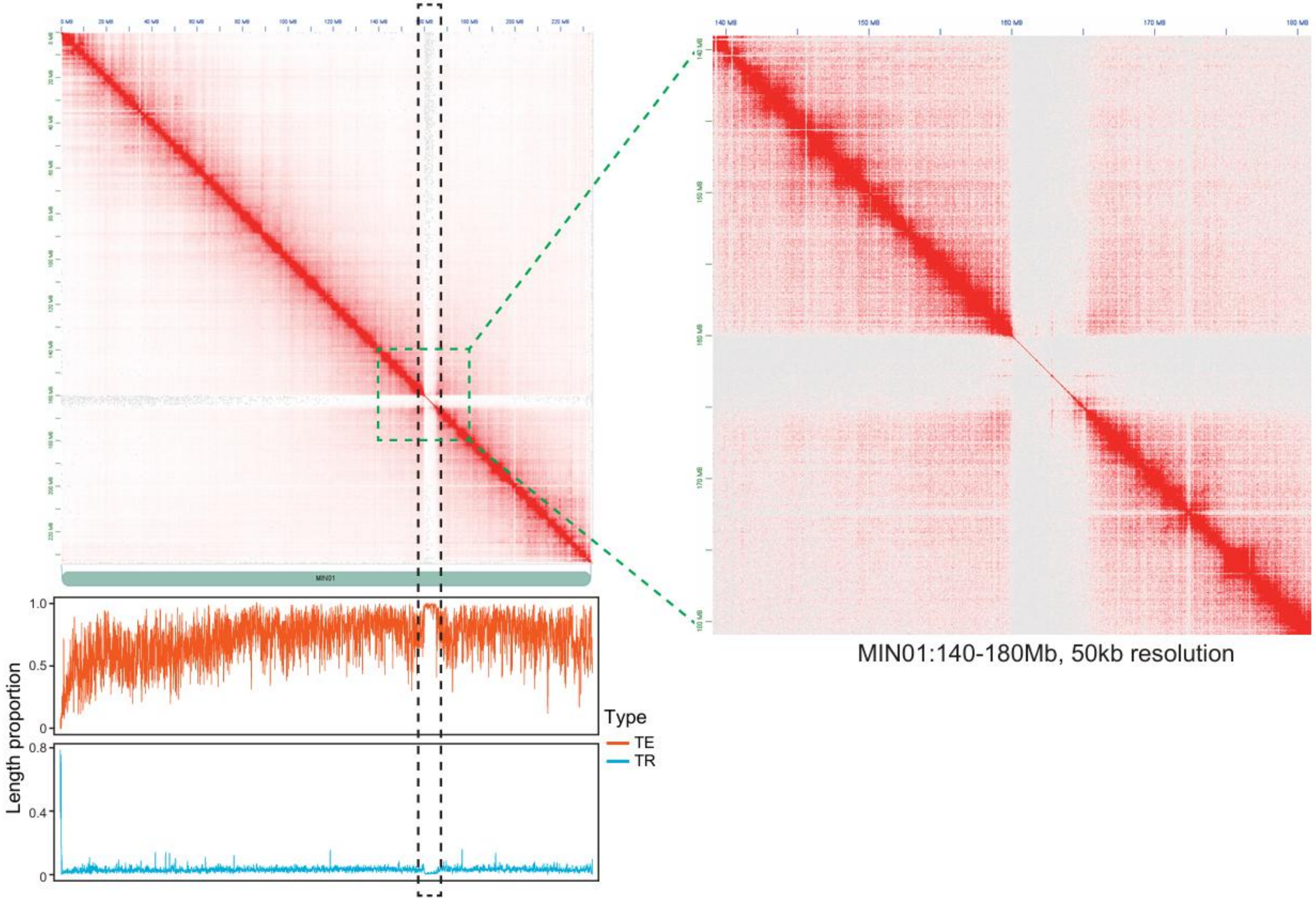

Supplementary Figure 29: The chromatin interactions in the Hi-C contact map and the distribution of TEs and tandem repeats (TR) on chromosome MIN01.

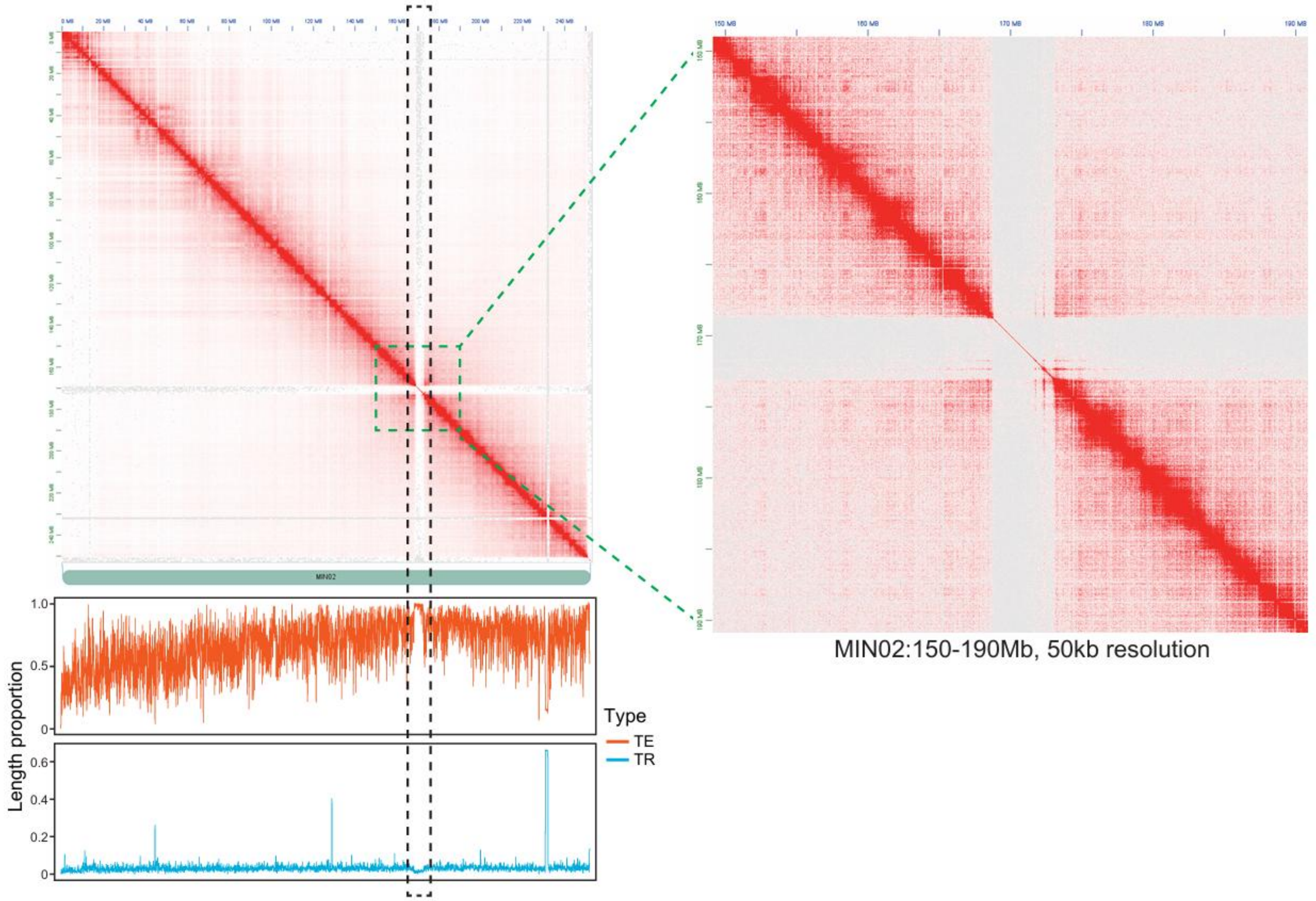

Supplementary Figure 30: The chromatin interactions in the Hi-C contact map and the distribution of TEs and tandem repeats (TR) on chromosome MIN02.

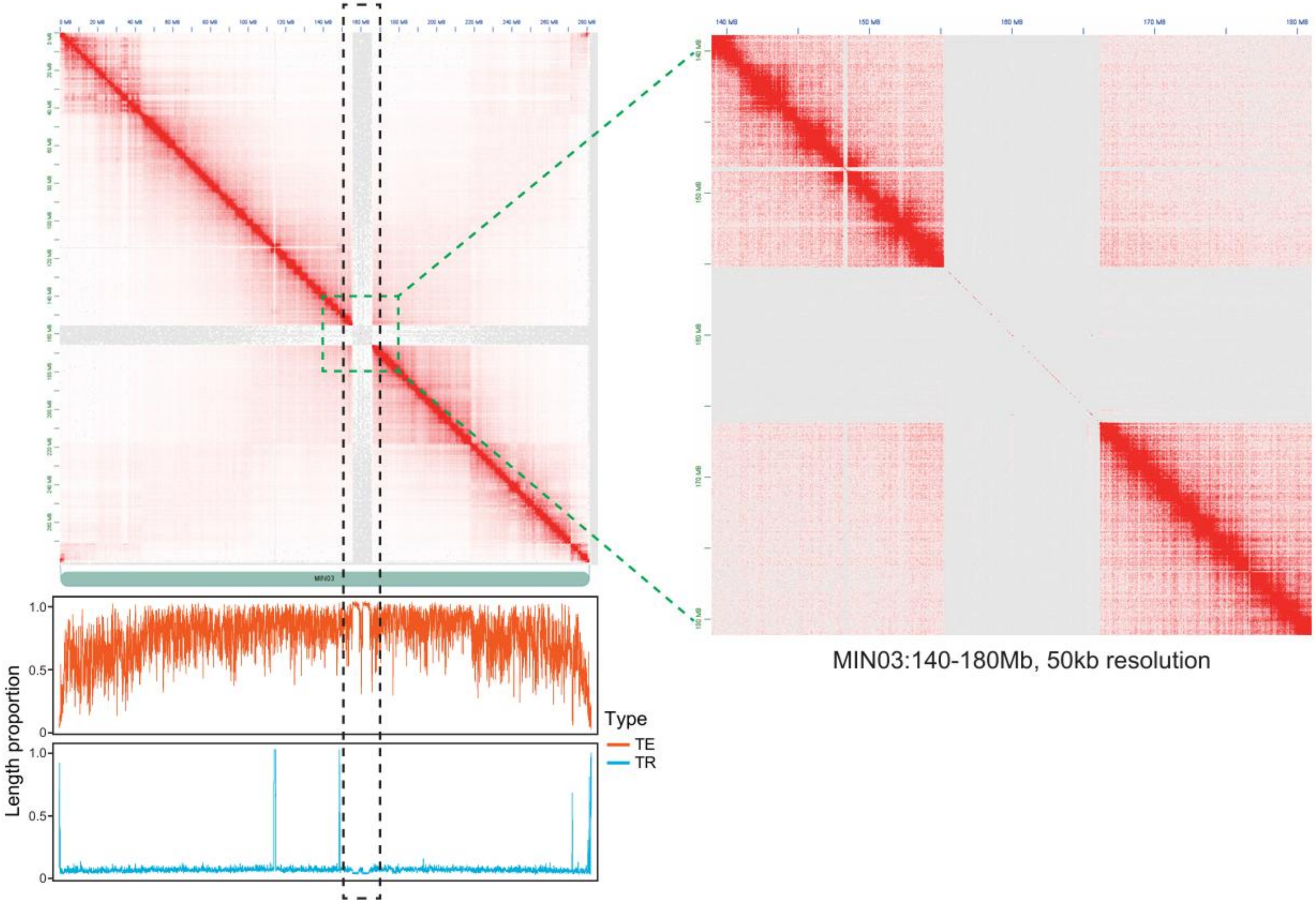

Supplementary Figure 31: The chromatin interactions in the Hi-C contact map and the distribution of TEs and tandem repeats (TR) on chromosome MIN03.

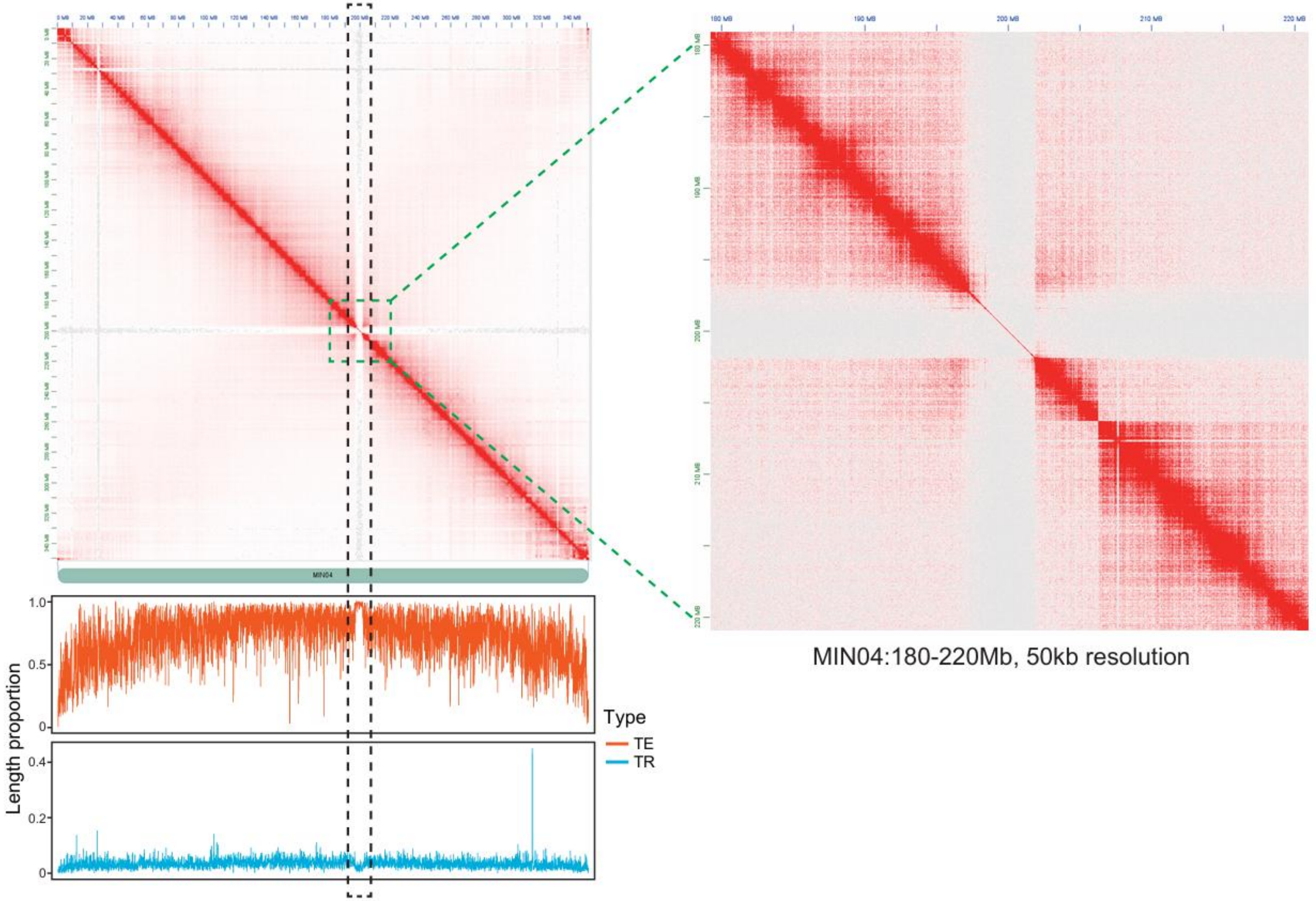

Supplementary Figure 32: The chromatin interactions in the Hi-C contact map and the distribution of TEs and tandem repeats (TR) on chromosome MIN04.

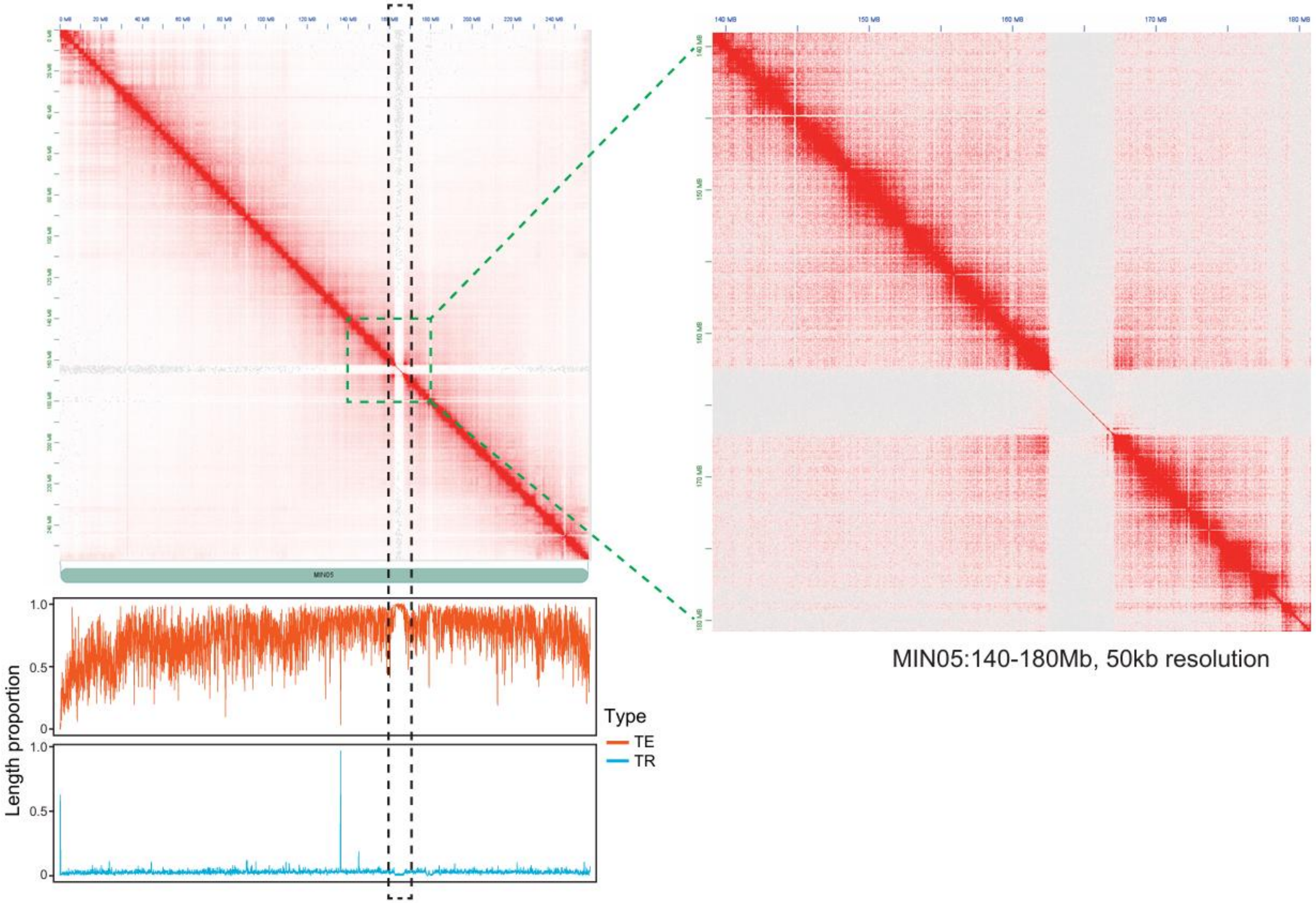

Supplementary Figure 33: The chromatin interactions in the Hi-C contact map and the distribution of TEs and tandem repeats (TR) on chromosome MIN05.

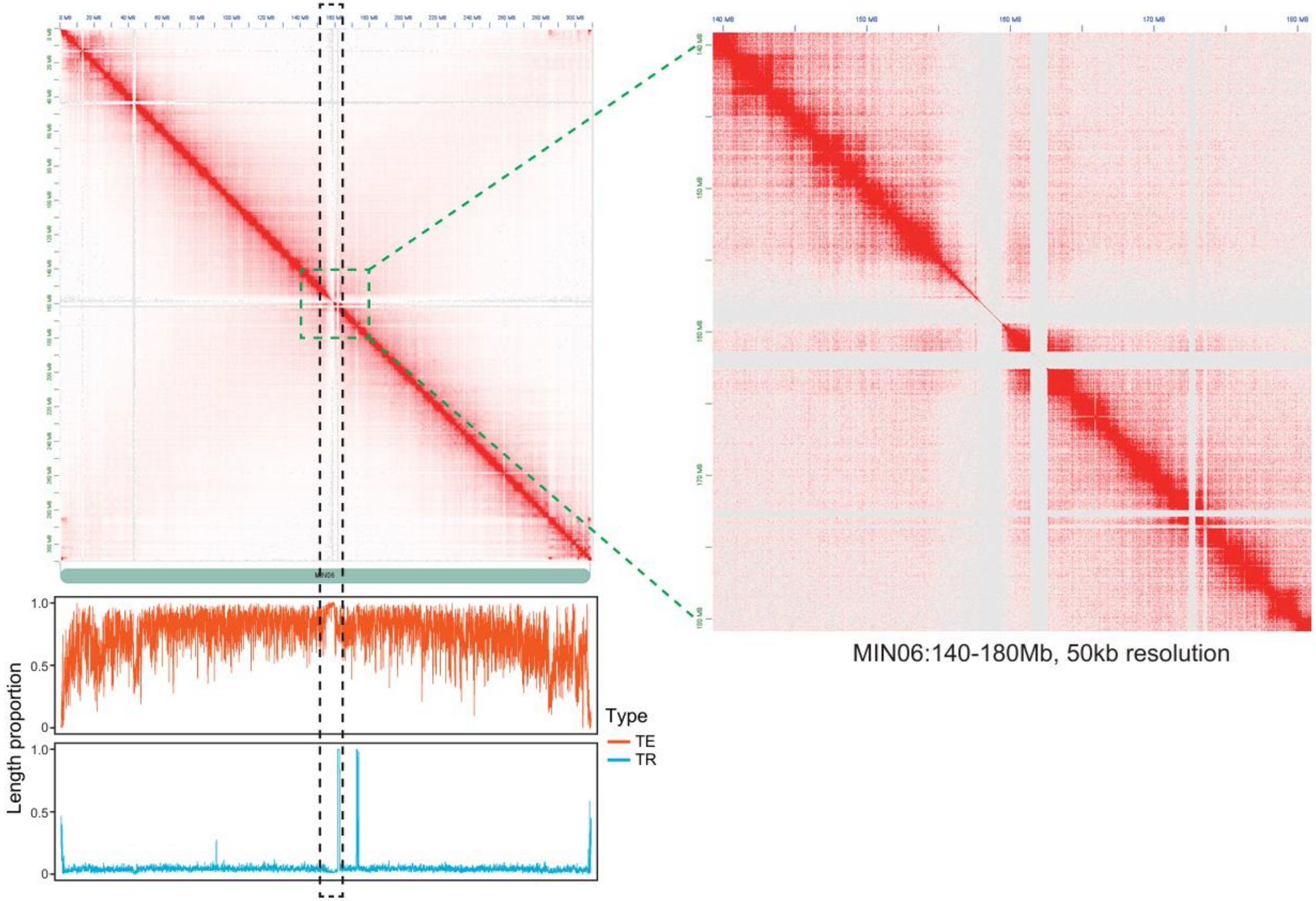

Supplementary Figure 34: The chromatin interactions in the Hi-C contact map and the distribution of TEs and tandem repeats (TR) on chromosome MIN06.

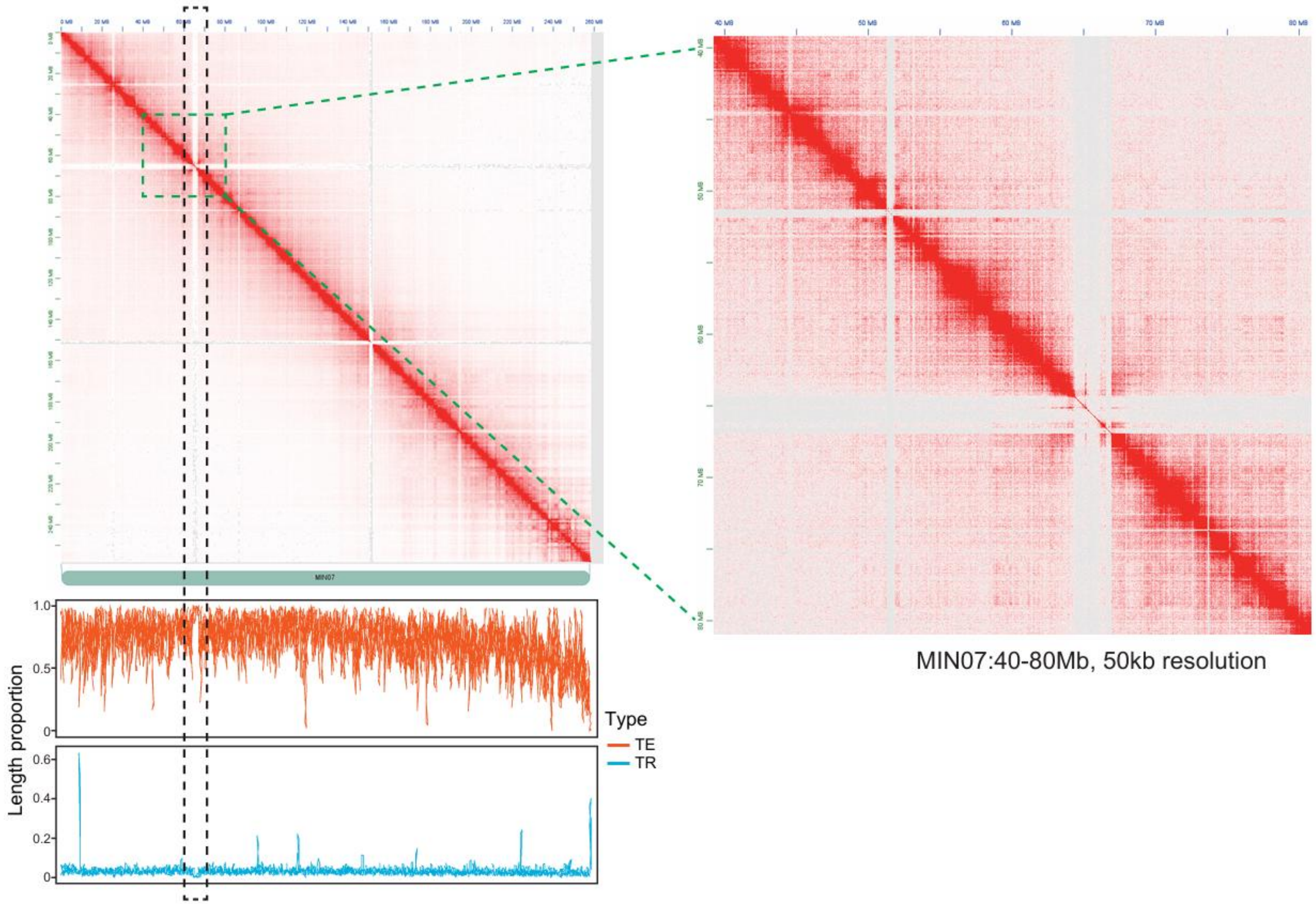

Supplementary Figure 35: The chromatin interactions in the Hi-C contact map and the distribution of TEs and tandem repeats (TR) on chromosome MIN07.

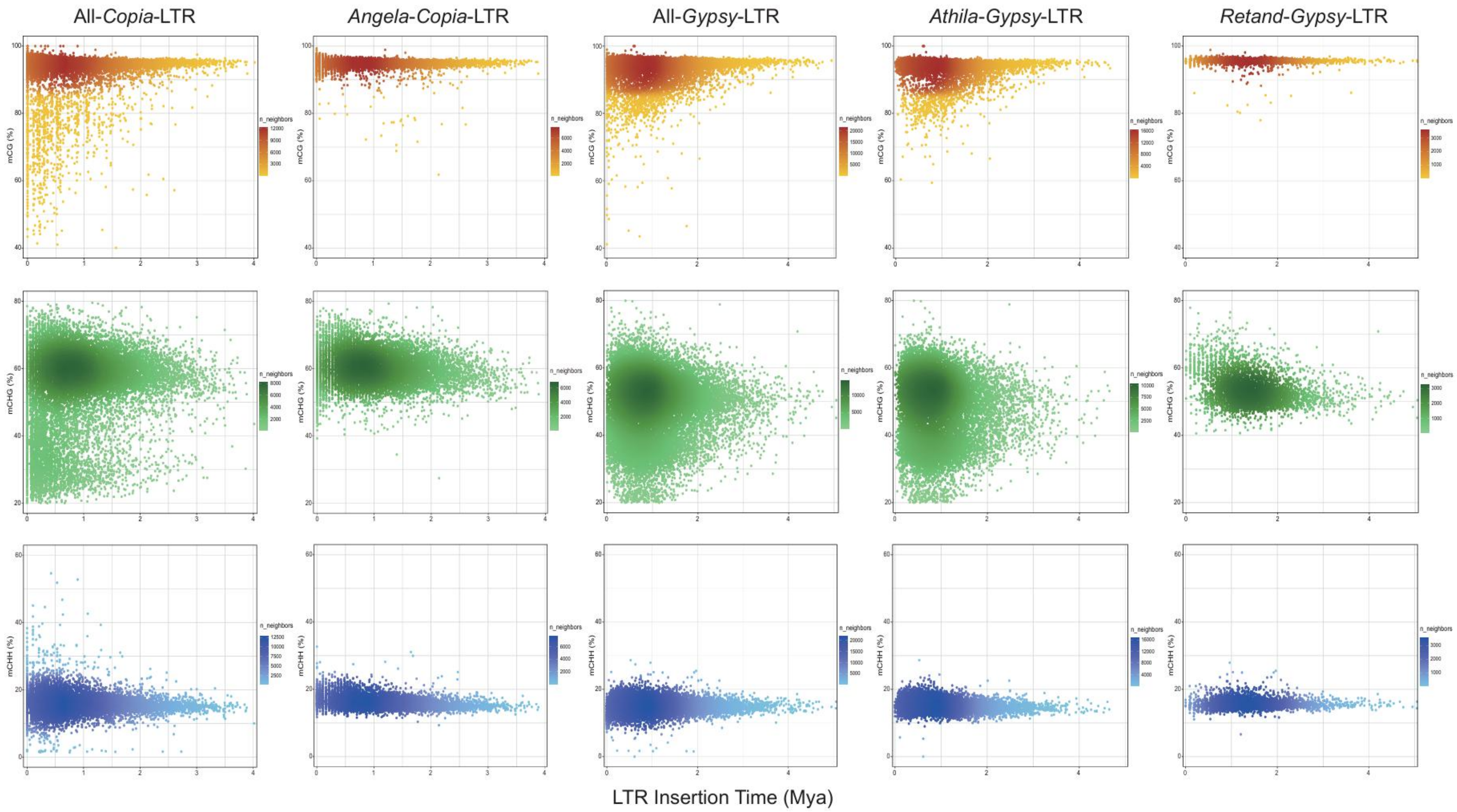

Supplementary Figure 36: The relationship between the LTR-RT insertion time and methylation modifications (mCG, mCHG, mCHH) in *M. incana*

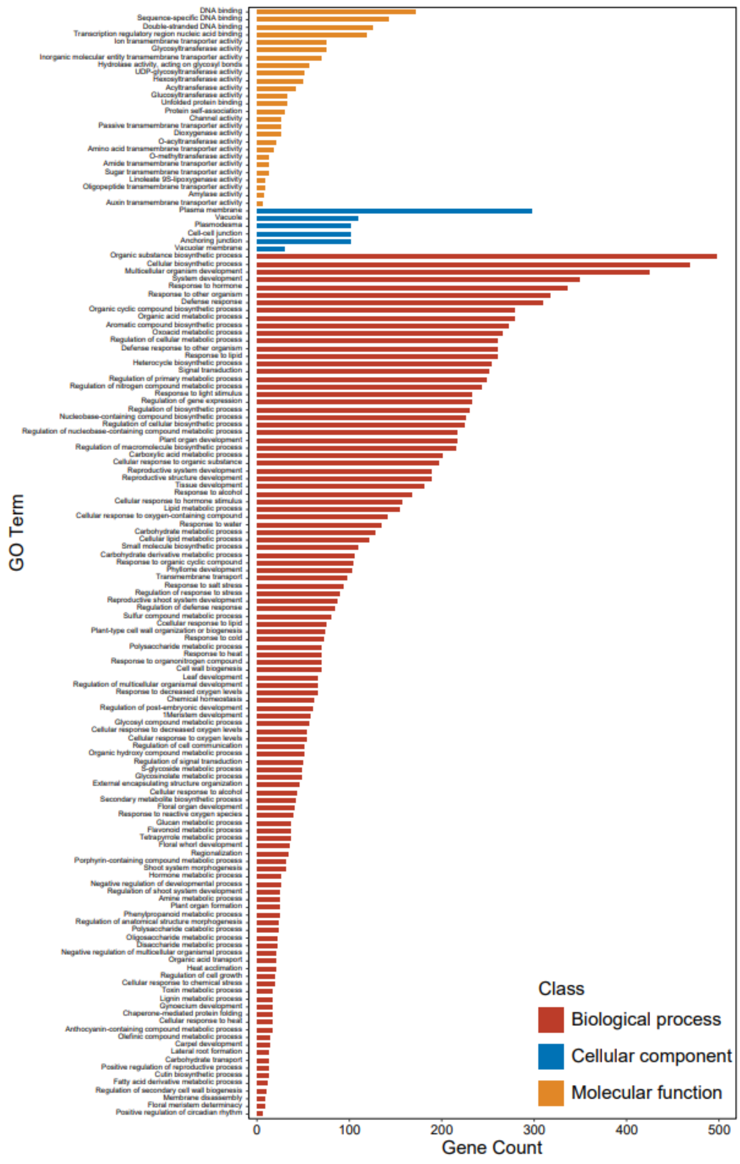

Supplementary Figure 37: GO enrichments results of co-DEGs.

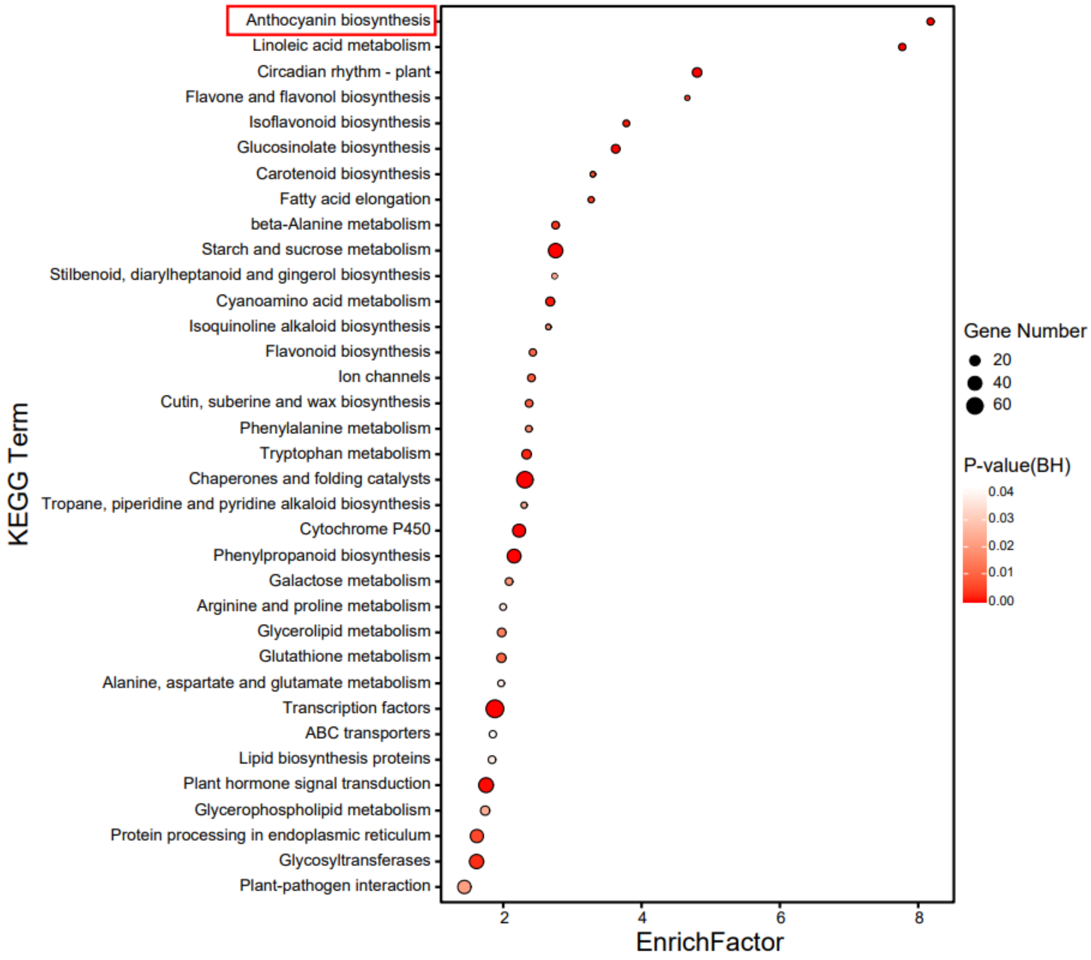

Supplementary Figure 38: KEGG enrichment results of co-DEGs.
